# Heteropolyaromatic Covalent Organic Frameworks via One-Pot Multicomponent Reactions

**DOI:** 10.1021/jacs.4c02551

**Published:** 2024-06-14

**Authors:** Prasenjit Das, Gouri Chakraborty, Nico Friese, Jérôme Roeser, Carsten Prinz, Franziska Emmerling, Johannes Schmidt, Arne Thomas

**Affiliations:** † Department of Chemistry/Functional Materials, Technische Universität Berlin, 10623 Berlin, Germany; ‡ BAM Federal Institute for Materials Research and Testing, Richard-Willstätter-Str. 11, 12489 Berlin, Germany

## Abstract

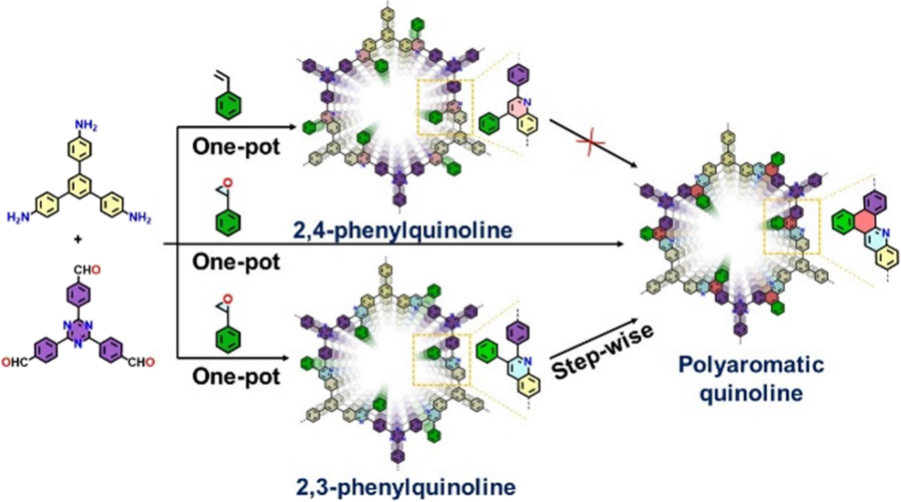

Multicomponent reactions (MCRs) offer a platform to create different chemical structures and linkages for highly stable covalent organic frameworks (COFs). As an illustrative example, the multicomponent Povarov reaction generates 2,4-phenylquinoline from aldehydes and amines in the presence of electron-rich alkenes. In this study, we introduce a new domino reaction to generate unprecedented 2,3-phenylquinoline COFs in the presence of epoxystyrene. This work thus presents, for the first time, structural isomeric COFs produced by multicomponent domino and Povarov reactions. Furthermore, 2,3-phenylquinolines can undergo a Scholl reaction to form extended aromatic linkages. With this approach, we synthesize two thermally and chemically stable MCR-COFs and two heteropolyaromatic COFs using both domino and in situ domino and Scholl reactions. The structure and properties of these COFs are compared with the corresponding 2,4-phenylquinoline-linked COF and imine-COF, and their activity toward benzene and cyclohexane sorption and separation is investigated. The position of the pendant phenyl groups within the COF pore plays a crucial role in facilitating the industrially important sorption and separation of benzene over cyclohexane. This study opens a new avenue to construct heteropolyaromatic COFs via MCR reactions.

## Introduction

Multicomponent reactions (MCRs) have emerged as a versatile synthetic strategy in the field of organic and medicinal chemistry.^1,2^ MCRs are generally regarded as one-pot reactions that involve the sequential transformation of at least three starting components into a desired product, thereby avoiding the need for multiple intermediate separation steps and laborious purification processes.^3^ In contrast to stepwise synthetic approaches, MCRs offer several advantages, including enhanced synthetic feasibility, greater structural diversity, expedient functionality, environmental friendliness and atom economy, all of which contribute to their sustainability.^3,4^ These unique features position MCRs as a crucial method for generating a wide range of new and diverse molecular products.

The recent progress in porous organic materials, especially, covalent organic frameworks (COFs) has witnessed remarkable advancements, driven by the development of innovative strategies to generate structurally diverse functional frameworks.^5^ Recently, the integration of MCRs into COF synthesis has emerged as a powerful tool to assemble intricate multiple building units, which is a challenging task using conventional approaches.^1,2,6^ This has opened exciting possibilities to form fused ring structures within COFs to generate highly stable and robust interlinked aromatic architectures, strengthening the entire framework. Consequently, the surface area, pore size, and functionality of the framework can be specifically controlled, which offers tailorable properties useful for a range of applications.

**Scheme 1:**
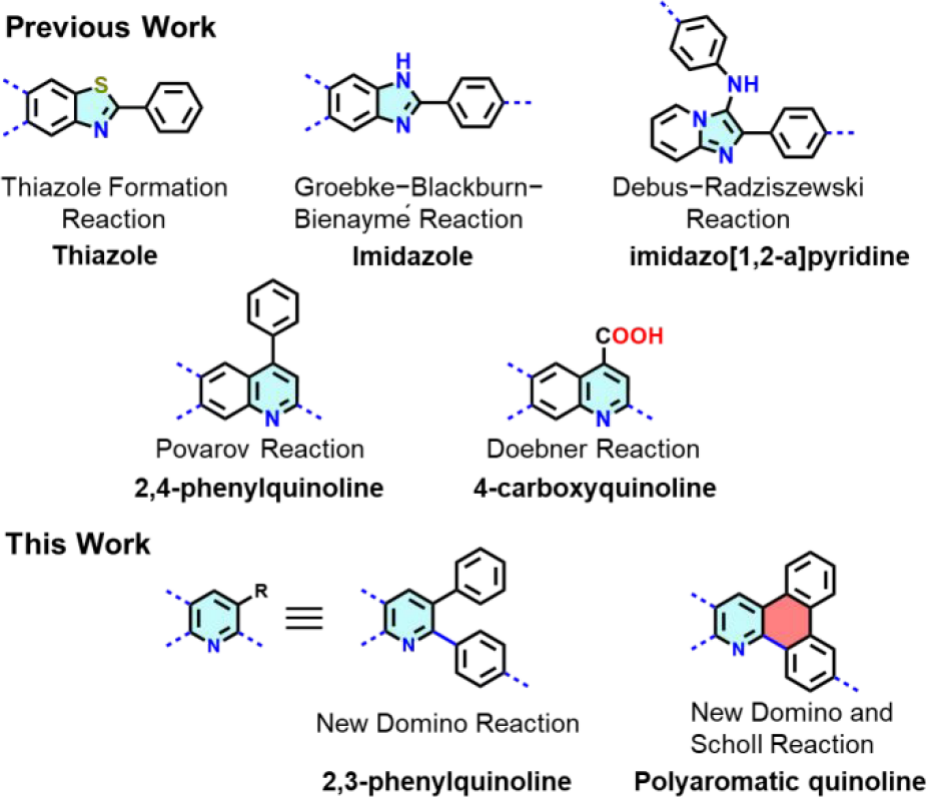
Development of Different Cyclic Linkages in MCR-COFs

So far, a couple of MCRs enabling ring-closing bond formation, namely, the Debus–Radziszewski reaction,^6^ Povarov reaction,^7,8^ thiazole formation reaction,^9^ Groebke–Blackburn–Bienaymé reaction,^10^ and Doebner reaction,^11^ have been employed for COF synthesis (see Scheme 1). Recently, our group has also contributed to report MCR-COFs based on Doebner and Povarov reactions which allow the formation of 2,4-substituted quinolines.^8,11a^ Quinoline and its derivatives have been of great interest due to their applicability in bioactive compounds,^12,13^ as well as in materials science application.^14^

While 2,4-diarylquinolines have been described as linkers in COFs formed via the Povarov reaction,^7,8^ the synthesis of 2,3-diarylquinoline has not been attempted in COF chemistry, setting the stage for our investigation. Unlike their 2,4-diarylquinoline counterparts, 2,3-diaryl derivatives exhibit a unique propensity for forming new bonds via Scholl-type reactions (Scheme 1). This formation process enables the creation of polycyclic aromatic fused ring compounds, characterized by distinct optoelectronic properties along with self-assembly behavior.^15^

To achieve our goal, we have designed a synthetic method for the synthesis of 2,3-diarylquinoline substituted COFs via a three-component domino reaction. Such COFs can in principle undergo a Scholl reaction by postsynthetic treatment or by a direct domino and Scholl reaction cascade to yield stable, crystalline, porous heteropolyaromatic COFs. For comparison, the analogous imine and 2,4-phenyl quinoline-linked COF were prepared. Also, the one-pot formation of the novel MCR-COF was compared with a stepwise postsynthetic modification of the corresponding imine COF. The MCR-COFs were tested for sorption and separation of industrially important solvents benzene (Bz) and cyclohexane (Cy).

## Results and Discussion

**Figure 1:**
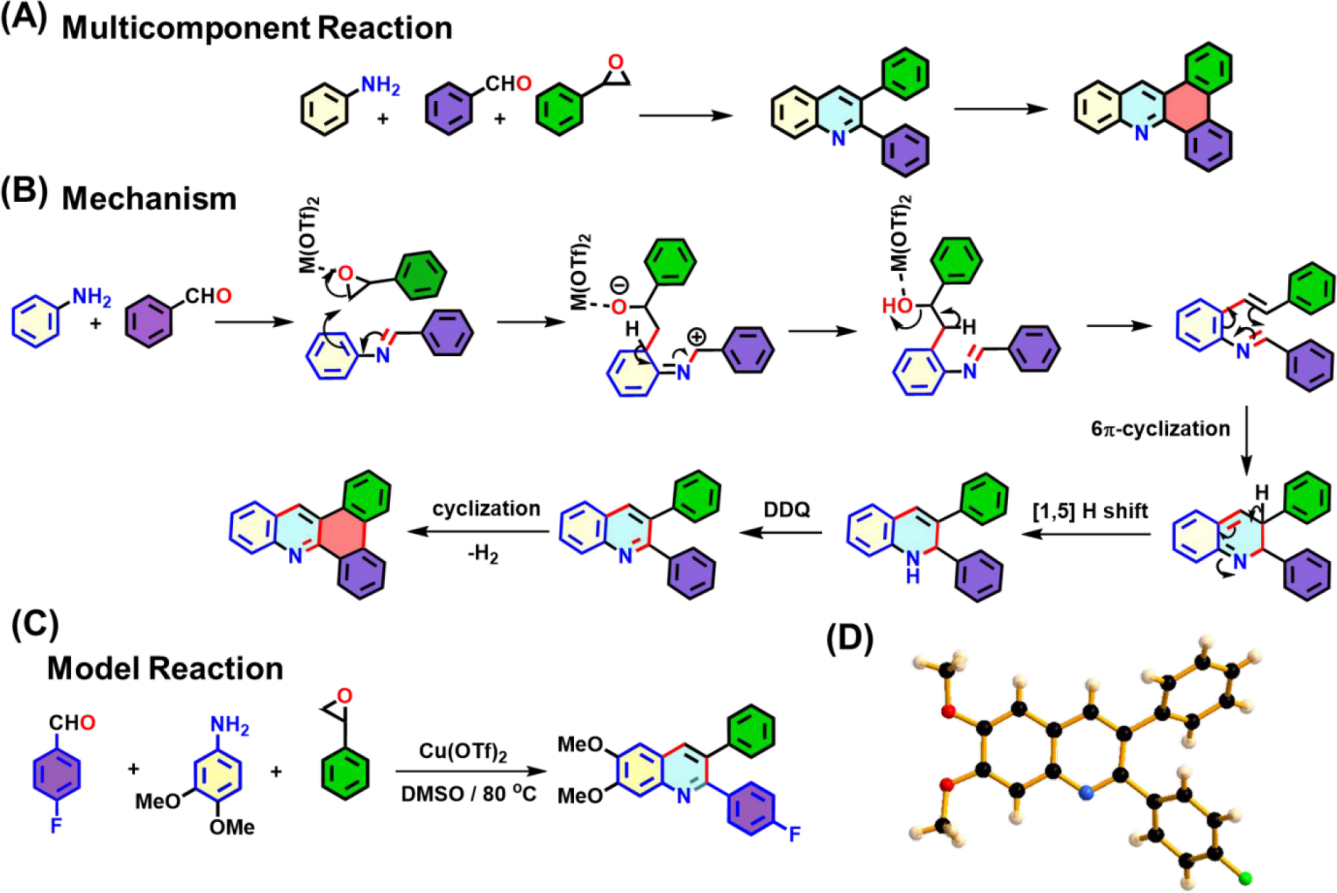
(A, B) Mechanism of the domino and in situ Scholl reaction; (C) model reaction; (D) crystal structure of the model compound.

**Figure 2:**
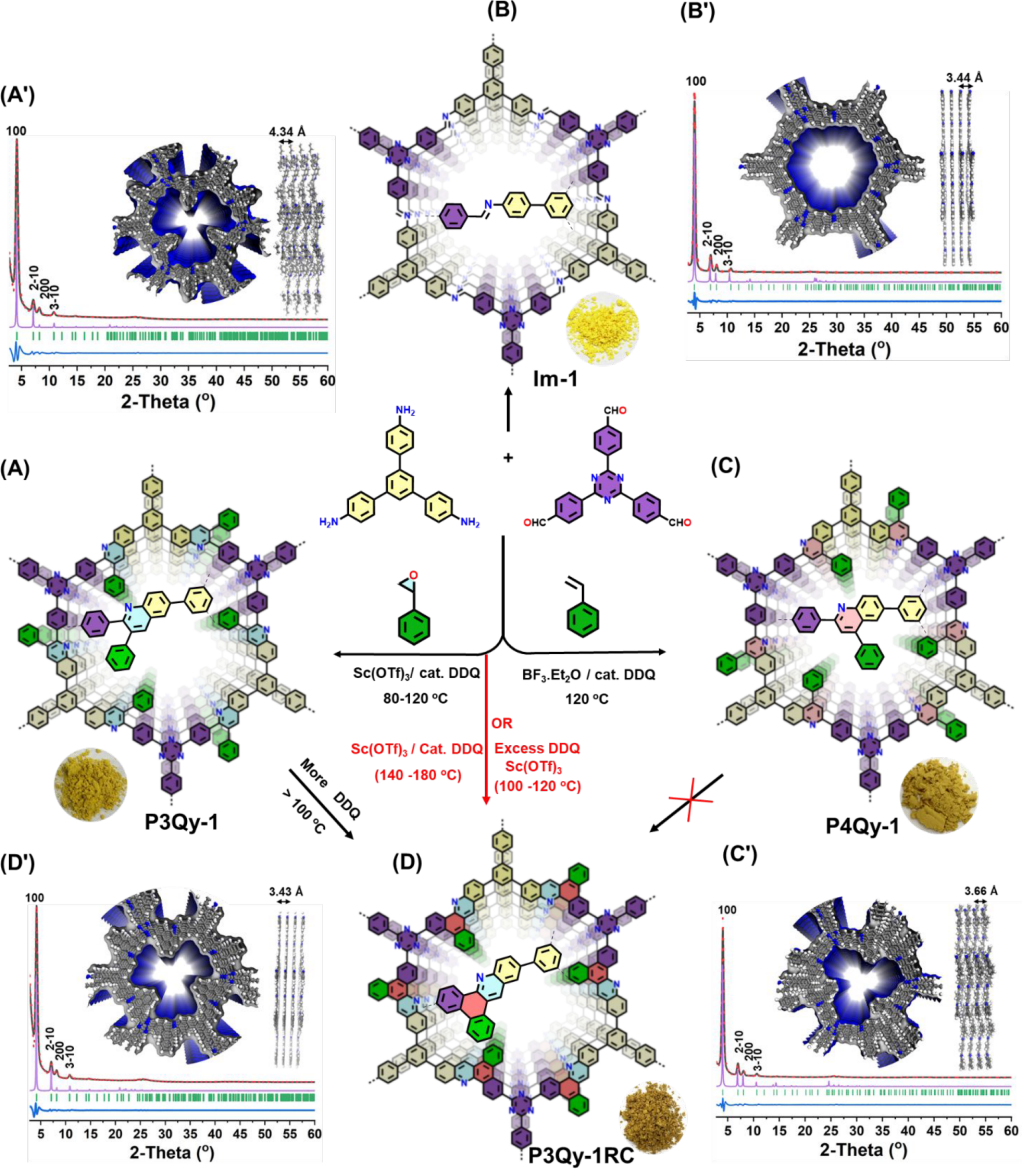
Synthesis of (A) **P3Qy-1**, (B) **Im-1**, and (C) **P4Qy-1**; (D) synthesis of fused-ring COFs via in situ and stepwise domino and Scholl reaction; Pawley refined (red dotted line) and experimental (black line) PXRD pattern with a minimum difference (blue line) for hexagonal AA stacking (pink line) of (A′) **P3Qy-1**, (B′) **Im-1**, (C′) **P4Qy-1**, and (D′) **P3Qy-1RC** (color code: C, gray spheres; N, blue spheres; H, white spheres).

The present study describes a novel COF from a three-component one-pot domino reaction involving an aldehyde and amine-functionalized building unit as well as an epoxide, resulting in the formation of 2,3-disubstituted quinoline which can undergo further Scholl reaction to form polyaromatic quinoline (Figure 1A). Figure 1B shows the proposed mechanism for the formation of a 2,3-disubstituted quinoline and polyaromatic quinoline moiety from this reaction.^16^ To verify this, a model MCR was performed using 4-fluorobenzaldehyde, 3,4-dimethoxyaniline, and styrene epoxide in DMSO, with Cu(OTf)_2_ as the catalyst at 80 °C (Figure 1C and Scheme S1). The desired model compound 2,3-disubstituted quinoline and in situ polyaromatic quinoline were synthesized successfully (synthetic details provided in the Supporting Information) and characterized using various analytical techniques, including single crystal X-ray diffraction, nuclear magnetic resonance (NMR) spectroscopy, and high-resolution mass spectrometry (HRMS) (Figures 1D and S1 and S2 and Table S1). The crystal structure of the model compound proves the formation of 2,3-diphenyl-substituted quinoline (Figure 1D). Based on this reaction, a novel MCR-based COF (**P3Qy**) was synthesized by employing the three-component domino reaction. The new family of MCR-COFs was compared with that of the Povarov reaction, which forms the 2,4-diphenyl-substituted quinoline (**P4Qy**) and the respective imine-based COF (**Im**) (Figure 2).

We have optimized the domino reaction to obtain highly crystalline COFs with a broad scope of Bro̷nsted or Lewis acid catalysts, different temperatures, solvents, and DDQ amounts. In the final reaction, 2,4,6-tris(4-aminophenyl)-1,3,5-benzene (TAB), 4,4′,4″-(1,3,5-triazine-2,4,6-triyl)tribenzaldehyde (TTA), and epoxy styrene (ES) were reacted in a solvent mixture of *o*-dichlorobenzene/*n*-butanol (1:1), in the presence of Sc(OTf)_3_ and DDQ at 120 °C for 72 h (Figure 2A, Scheme S3). The reaction yielded a lemon-yellow-colored COF, linked by 2,3-phenyl-substituted quinoline units (**P3Qy-1**), in 75% yield. It is observed that using the same reactants and solvents but varying the temperature from 80 to 120 °C or changing the reaction time or the Lewis acid catalyst from Sc(OTf)_3_ to Cu(OTf)_2_, **P3Qy-1** is formed exclusively (Figure S3, Table S2). Increasing the reaction time to 3 days and elevating the temperature to 120 °C enhances the crystallinity and long-range order of the desired COF. Using the same starting reactants and similar reaction conditions in the absence of ES, M(OTf)_*x*_, and DDQ, the analogous imine-COF (**Im-1**, yellow color) was formed with 86% yield (Scheme S4, Figure 2B). Additionally, we prepared the corresponding Povarov reaction product (**P4Qy-1**, yellow-brown color) by generating 2,4-phenylquinoline in the presence of styrene, with a yield of 73% (Scheme S5, Figure 2C).

2,3-Phenylquinolines can undergo aryl–aryl oxidative coupling to form the geometrically favored fused heteropolyaromatic compound. Therefore, the same reaction was conducted at 140 °C using a catalytic amount of DDQ. A cascade domino and Scholl reaction would result in the formation of novel heteropolyaromatic MCR-COF (**P3Qy-1RC**) (RC = ring closing; Scheme S6, Figure 2D). Elevated temperatures promote the activation of the aromatic substrates, making them more reactive and facilitating the formation of carbon–carbon bonds. We conducted experiments to investigate the effect of different amounts of DDQ and observed that a catalytic amount is sufficient for the formation of the aromatic quinoline bridge. However, increasing the amount of DDQ further enhances the cyclo-aromatization in the adjacent phenyl group (in situ Scholl reaction) even at a lower temperature (120 °C). It should be noted that higher amounts of DDQ with high temperatures (>150 °C) led to a decrease in the crystallinity of the system, likely due to the rapid formation of the aromatic quinoline bridge, which forms irreversible strong bonds (Figure S4, Table S3).

Based on this insight, we proceeded to synthesize an additional series of heteropolyaromatic MCR-COFs. In these new systems, we replaced TAB with 2,4,6-tris(4-aminophenyl)-1,3,5-triazine (TAT), which was subjected to a temperature of 120 °C for 72 h to form **P3Qy-2**, a golden yellow COF, with 75% yield (Scheme S7, Table S2). Furthermore, by employing either 150 or 120 °C along with an increased amount of DDQ, we prepared heteropolyaromatic **P3Qy-2RC** (olive yellow-brown color, 70% yield) (Scheme S8, vide supra, Table S3).

The determination of the structure and crystallinity of all COFs was accomplished through analysis of their powder X-ray diffraction (PXRD) patterns. All COFs exhibited well-defined diffraction peaks, indicative of high crystallinity. The most intense peak is observed at approximately 4.1° 2θ, with less intense peaks appearing at approximately 7.1°, 8.1°, and 10.7° 2θ. These peaks correspond to reflections from the (100), (21̅0), (200), and (31̅0) facets, respectively (Figure 2A′–D′). The simulated model structures are consistent with an eclipsed AA-stacking, and the Pawley refinement demonstrated good agreement with negligible error for all COFs (Figures S5–S10). The unit cell parameters were determined for **P3Qy-1** (*a* = *b* = 25.29 Å, *c* = 4.34 Å, trigonal P3), **P3Qy-1RC** (*a* = *b* = 25.31 Å, *c* = 3.43 Å, hexagonal *P*6̅), **P3Qy-2** (*a* = *b* = 25.19 Å, *c* = 4.39 Å, trigonal *P3*), **P3Qy-2RC** (*a* = *b* = 25.18 Å, *c* = 3.43 Å, hexagonal *P*6̅), **P4Qy-1** (*a* = *b* = 25.45 Å, *c* = 3.66), and **Im-1** (*a* = *b* = 25.33 Å, *c* = 3.44 Å, hexagonal *P*6̅), with α = β = 90°, γ = 120° from Pawley refinement (Table S4). A broad and weak reflection at ∼25 to 26.5 2-theta (deg) indicating a distribution of interlayer distances in the range of 3.5 Å confirmed the presence of π–π stacking to form 2D layered structures for both MCR- and imine-based COFs. For the quinoline COFs **P3Qy-1** and **P3Qy-2**, a larger interlayer distance would be assumed from calculations and confirmed by HR-TEM (Figure 4A, vide infra). However, also for these COFs, multiple broad reflections are found in the higher 2-theta region, indicating the presence of a significant amount of stacking disorder with a smaller interplanar distance compared to the distance expected from the ideal crystal lattice. The stacking of the COF sheets changes from 4.34 to 3.43 Å during the last reaction step, which is most probably due to the flattening of the linkage by the ring closure of the Scholl reaction. Indeed, the dangling phenyl ring in **P3Qy-COFs** must rotate out of the plane of the COF layer due to steric hindrance, which might prevent space-efficient layer stacking. This steric hindrance is less pronounced for the phenyl groups in **P4Qy-1** seen in the shorter stacking distance. The formation of the heteropolyaromatic motif from the Scholl reaction should yield very rigid and easily packable aromatic COF layers, which could explain the stacking distance of **P3Qy-COFs** very close to graphite. The simulated AB and ABC structures and their corresponding PXRD patterns did not align with the experimental PXRD patterns of both MCR and imine-based COFs.

**Figure 3:**
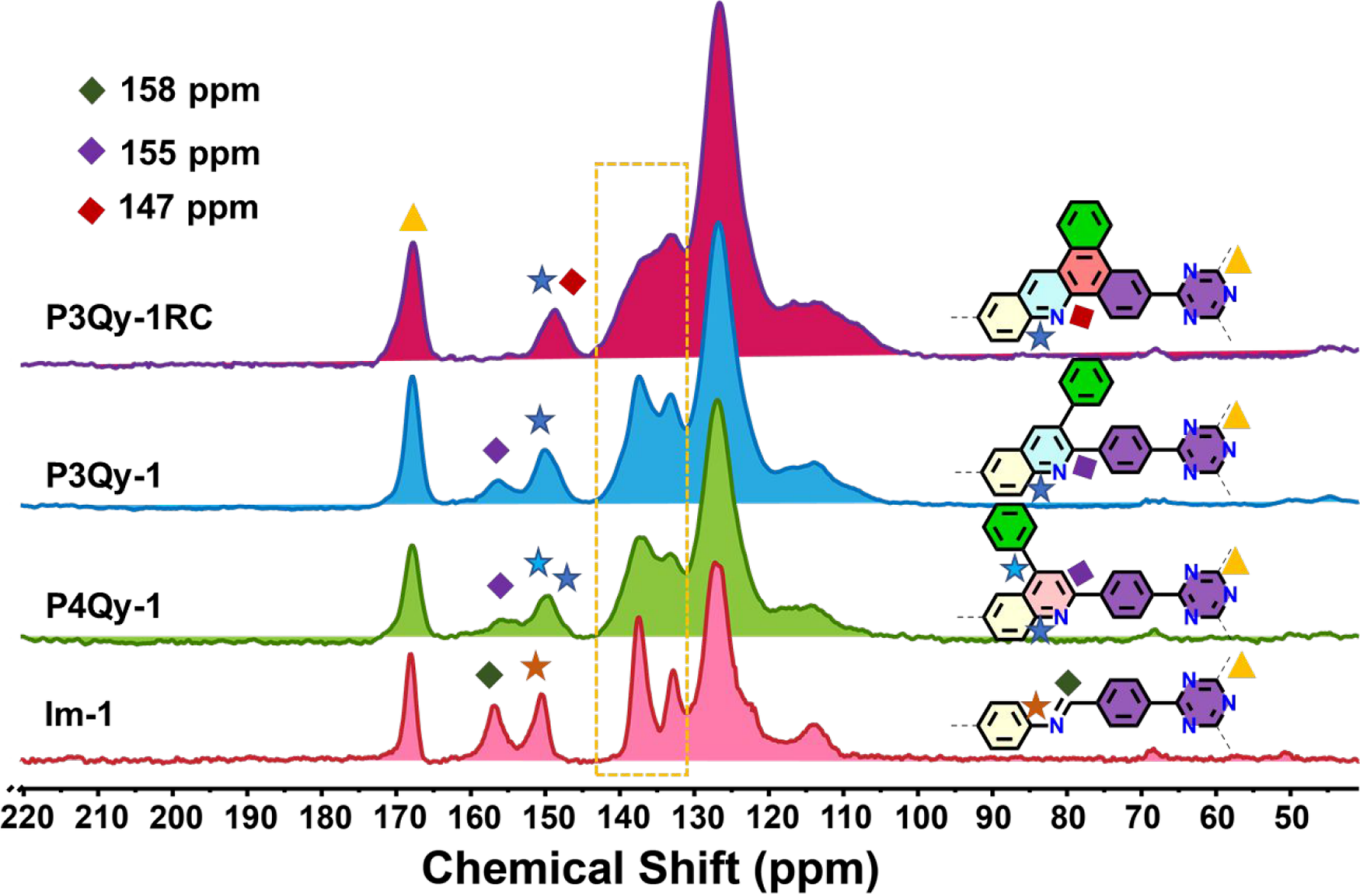
^13^C (CP/MAS) NMR spectra measured at 10 kHz. The brown dotted box reflects the broadening of the NMR spectra in **P3Qy-1RC** compared to **P3Qy-1**, attributed to the restricted rotation after ring formation.

The formation of the new aromatic core in the COF-linkage was investigated by using elemental analysis and solid-state cross-polarization magic-angle-spinning (CP-MAS) ^13^C NMR, Fourier transform infrared (FTIR), and X-ray photoelectron (XPS) spectroscopy. The FTIR spectra of **P3Qy**-**1**, **P3Qy-1RC**, **P3Qy**-**2**, and **P3Qy-2RC** exhibited strong distinct peaks near 1597 cm^–1^, corresponding to stretching frequencies from the quinoline functional groups (Figure S11). In contrast, this peak was absent in the spectrum of **Im-1**, which instead displayed a −C=N stretching frequency at 1577 cm^–1^ (Figure S11). The solid-state ^13^C NMR spectrum of **Im-1** exhibited a characteristic peak at 158 ppm, indicative of the presence of an imine group. This peak is shifted for **P3Qy-1**, **P3Qy-2**, and **P4Qy-1** to 155 ppm indicates the generation of quinoline groups. The presence of a characteristic peak at 168 ppm in the NMR spectra of all COFs confirms the presence of a triazine group (Figures 3 and S12). In the case of **P3Qy-1RC** and **P3Qy-2RC**, the in situ Scholl reaction results in a significant reduction of the quinoline peak at 155 ppm, which is shifted to 147 ppm (Figures 3 and S12). The broad peak around 147 ppm depicts the formation of new bonds and generation of quaternary carbon due to the Scholl reaction in **P3Qy-1RC** and **P3Qy-2RC** (Figures 3 and S12). The peak region between 143 and 130 ppm in the NMR spectra is broadened when comparing **P3Qy-1** to **P3Qy-1RC**. This suggests a restriction in the rotation of the pendant phenyl group after the formation of fused rings. The formation of **P3Qy-1** and **P3Qy-1RC** under different conditions yielded similar results in solid-state ^13^C NMR. However, the stepwise conversion from **P3Qy-1** to **P3Qy-1RC** did not show a complete conversion (>80% completion) presumably due to dense stacking of COF layers (Figure S12). However, recent research achieved the postsynthetic Scholl reaction on a 3D COF with exposed imine groups.^17^ Elemental analysis of both imine and MCR-COFs demonstrated a close agreement between the theoretical and experimental values (see the synthesis part of SI). XPS spectra further validated the formation of quinoline linkages in MCR-COFs compared to imine COF. The N 1s spectra exhibited a characteristic peak at 400.1 eV corresponding to the quinoline nitrogen, which is absent in **Im-1**, where only one peak for the triazine and imine nitrogen was observed due to their similar binding energies (398.5 eV) (Figure S13).^7^ The N 1s signal in the spectra of MCR-COFs is much broader compared to **Im-1**, indicating the presence of more than one nitrogen species—in this case, triazine and quinoline, where **P3Qy-RC** is slightly broader than those of both **P3Qy** and **P4Qy**. The variation of the binding energies of quinolines in the MCR-COF can originate from the different chemical environments due to the ring closure.

**Figure 4:**
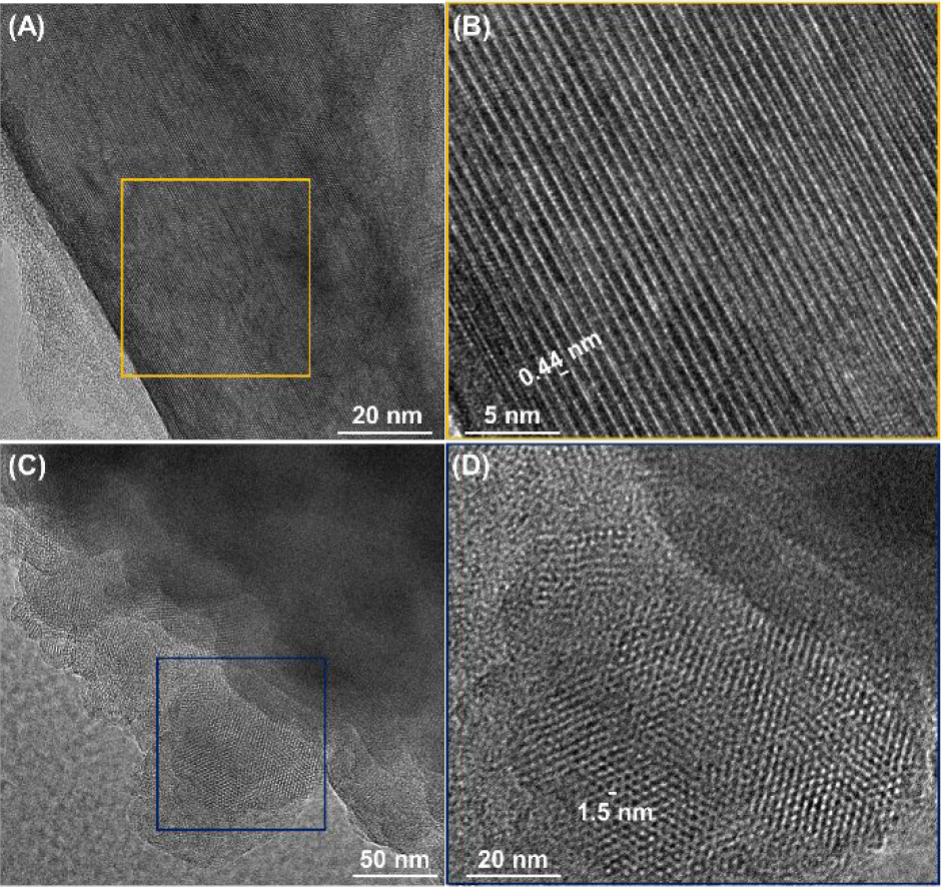
HRTEM images of (A) **P3Qy-1** and (B) its magnified view and (C) **P3Qy-1RC** and (D) its magnified view.

Field emission scanning electron microscopy (FESEM) and high-resolution transmission electron microscopy (HRTEM) display uniform morphologies and ordered networks for imine and MCR-COFs. The FESEM image of **Im-1** shows a porous spherical morphology (Figure S14). FESEM images of the MCR-COFs show different morphologies from porous spherical to microflower-like structures (Figures S15–S19). The HRTEM images revealed ordered crystalline domains in all MCR and imine COFs, which is coherent with the FFT pattern and interlayer spacing of the simulated pattern (Figures 4 and S20–S23). Using low-dose HRTEM, **P3Qy-1** shows ordered lattice fringes with an interplanar *d*-spacing of 0.44 ± 0.01 nm, which correlates well to the interlayer spacing of the simulated pattern (Figure 4A,B). Low-dose HRTEM of **P3Qy-1RC** shows hexagonal pores with a distance of 1.5 ± 0.01 nm, which correlates with the pore size of **P3Qy-1RC** (Figure 4C,D).

**Figure 5:**
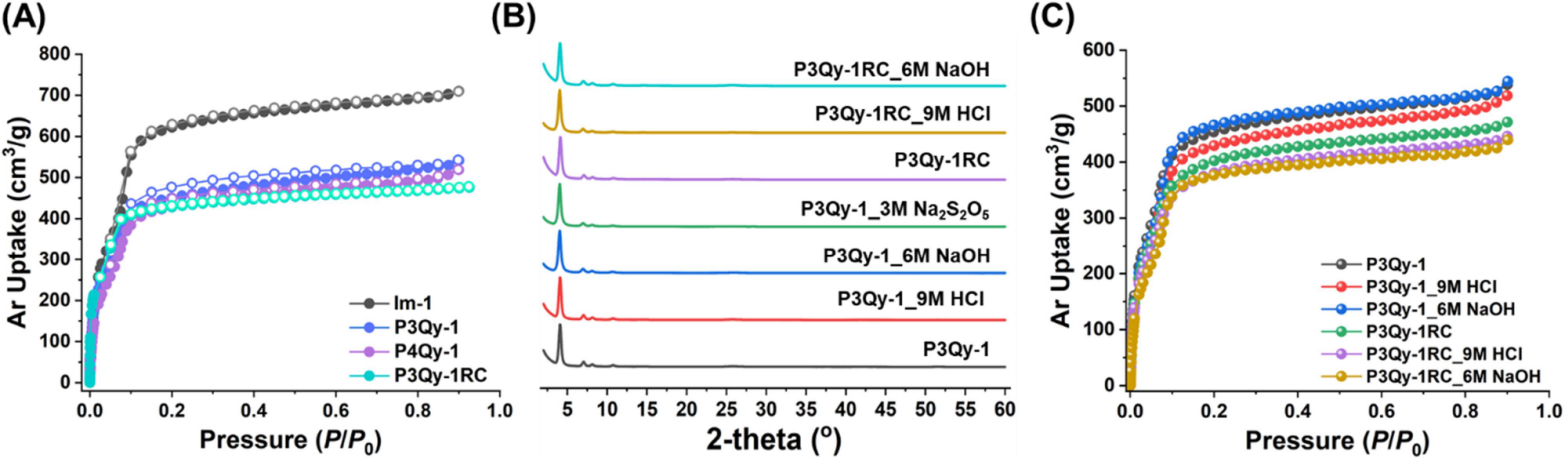
Surface area and permanent stability of MCR-COFs: (A) Ar sorption isotherm of **Im-1**, **P3Qy-1**, **P3Qy-1RC**, **P4Qy-1** at 87 K. Chemical stability of **P3Qy-1** and **1RC** in 9 M HCl, 6 M NaOH, and 3 M Na_2_S_2_O_5_ for 7 days monitored by (B) PXRD and (C) Ar sorption at 87 K.

All MCR-COFs exhibit characteristic microporous type-I sorption isotherms (Figure 5A) from which the Brunauer–Emmett–Teller (BET) surface area and pore volume of **Im-1**, **P3Qy-1**, **P3Qy-1RC**, and **P4Qy-1** can be determined to be 2033, 1590, 1570, and 1590 m^2^ g^–1^, and 0.89, 0.76, 0.75, and 0.74 cm^3^ g^–1^, respectively. Similarly, the BET surface area for **P3Qy-2** and **P3Qy-2RC** was found to be 1650 and 1560 m^2^ g^–1^, respectively (Figure S24). The pore size was calculated by nonlinear density functional theory (NLDFT). **Im-1** shows a higher gas uptake compared to the MCR-COF and a pore size of 21 Å, which is in good agreement with the simulated one (Figure S25). For all MCR-COFs smaller and bimodal pore size distributions are found with maxima around 16.0 and 20.5 Å which might be explained by the pendant phenyl groups leading to an irregular pore geometry. All MCR-COFs exhibit high thermal and chemical stability due to the formation of quinoline and fused heteropolyaromatic linkages. Thermogravimetric analysis (TGA) revealed that all imine and MCR-COFs display no weight loss up to ∼500 and ∼400 °C under N_2_ and air, respectively (Figure S26). Furthermore, the chemical stability of the COFs was investigated by treating them with 9 M HCl (aq), 6 M NaOH, and a reducing agent (Na_2_S_2_O_5_) for 7 days. PXRD measurements showed identical patterns after these treatments, and no weight loss was observed (Figures 5B and S27–S29). To assess the influence of these treatments on porosity, Ar sorption measurements at 87 K were carried out for **P3Qy-1** and **P3Qy-1RC** after all treatments (Figure 5C). The Ar sorption isotherms are very similar showing just a very low decrease in BET surface areas, confirming the robustness of the substituted quinoline and heteropolyaromatic-quinoline systems. While **P4Qy-1** showed comparable thermal and chemical stability, **Im-1** exhibited a lower stability (Figure S29).

The synthesis of the 3-phenyl substituted quinoline linkage via postsynthetic modification (PSM) of **Im-1**, involving the reaction of the imine COF with ES, DDQ, and Sc(OTf)_3_ in a solvent mixture of *o*-dichlorobenzene/*n*-butanol (1:1) at 120 °C for 48 h, resulted in a COF with relatively low crystallinity and surface area (see Supporting Information Figures S30 and S32). Furthermore, the solid-state ^13^C NMR spectrum of PSM-COF shows a small peak at 158 ppm, indicative of the presence of an imine group, showing that full conversion was not achieved presumably due to dense stacking of COF layers. When the same reaction was conducted at 150 °C for 48 h to achieve an in situ PSM and Scholl reaction in **Im-1**, a significant decrease in crystallinity to nearly amorphous materials was observed (Figure S32). The reason why a one-pot process provides materials with higher structural quality in comparison to the two-step process has been discussed before.^9^ In the two-step process, the epoxystyrene has to react with the imine groups in the preformed imine COF with predetermined crystallinity, which might be hindered by slow mass diffusion through the pores and the added compound needs a certain orientation to the imine bond to achieve the cyclization, which might be complicated within small pores. In contrast, for the one-pot reaction, it is assumed, that aldehyde and amine undergo reversible reactions to form first small oligomers, on which cyclization with epoxy styrene can occur to form reversible dihydroquinoline before slow irreversible oxidation occurs to form quinoline-derived extended frameworks via π–π stacking, resulting in highly crystalline and porous structures. This emphasizes that the one-pot reaction not only offers the advantage of reduced processing steps but also yields more defined materials.^9^

The optoelectronic properties of all of the COFs were analyzed by solid-state UV–vis diffuse reflection spectra (UV–vis DRS). UV–vis DRS showed a red-shift from **Im-1** to **P3Qy-1** to **P3Qy-1RC** due to the increasing conjugation of the quinoline center, which is in good agreement with the observed color of the COFs (Figure S33). Using the Tauc plot method, the corresponding optical band gap was calculated to be 2.67, 2.60, and 2.42 eV for **Im-1**, **P3Qy-1**, and **P3Qy-1RC**, respectively (Figure S34). Similarly, a red-shift was observed from **P3Qy-2** to **P3Qy-2RC** with optical band gaps of 2.67 and 2.28 eV (Figures S35 and S36).

**Figure 6:**
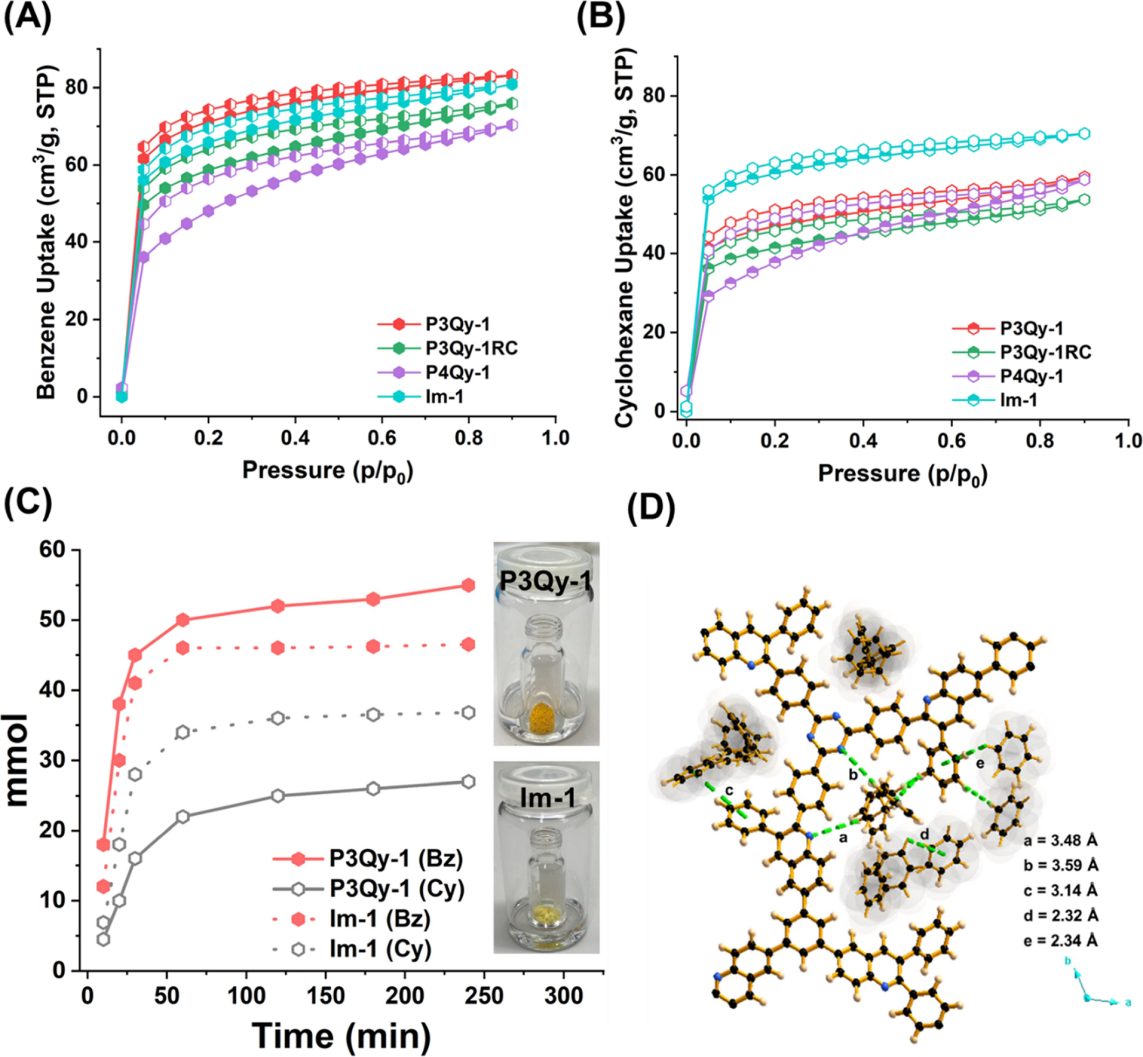
(A) Benzene (Bz) and (B) cyclohexane (Cy) sorption isotherms of **P3Qy-1**, **P3Qy-1RC, P4Qy-1**, and **Im-1** at 298 K. (C) Uptake selectivity of benzene over cyclohexane monitored over time (inset: experimental setup). (D) Interaction of Bz with **P3Qy-1** from CBMC simulation (C: black, H: off yellow, and N: blue).

Benzene (Bz) is the pivotal chemical feedstock for the synthesis of cyclohexane (Cy).^18^ The separation of Bz and Cy presents a significant challenge in petrochemical engineering, owing to their closely matched thermodynamic properties like nearly identical boiling points (Bz, 353.3 K; Cy, 353.9 K), closely resembling molecular volumes and geometries, comparable Lennard-Jones collision diameters, and minimal differences in relative volatilities.^18^ This convergence of characteristics necessitates advanced separation techniques beyond conventional methods. Various strategies involving the incorporation of open metal sites in metal organic frameworks (MOFs), presence of anions or cations, suitable pore space in MOFs or macromolecules, and electron-rich and electron-deficient moieties in COFs have been utilized to adsorb Bz over cyclohexane efficiently.^19^ However, the presence of pendant benzene pore-functionality in porous systems such as MCR-COFs remained unexplored, prompting us to investigate aromatic solvent sorption, primarily benzene (Bz), and understand its sorption and separation in relation to cyclohexane (Cy). To test the Bz/Cy sorption behavior, single-component Bz and Cy sorption was performed at 298 K. All isotherms exhibit a characteristic type I profile, and the uptake of benzene is generally higher than that of cyclohexane in all COFs. The uptake of Bz and Cy at *p*/*p*_0_ = 0.9 were found to be 83.1 and 59.4; 75.9 and 53.6; 70.3 and 58.7; and 80.4 and 70.4 cm^3^ g^–1^ for **P3Qy-1**, **P3Qy-1RC**, **P4Qy-1**, and **Im-1**, respectively (Figures 6A,B and S37). Among all COFs, **P3Qy-1** shows the largest difference in Bz sorption compared to Cy and thus the highest Bz selectivity. The uptakes of Bz and Cy at *p*/*p*_0_ = 0.9 were found to be 94.9 and 67.7; 76.3 and 53.4 cm^3^ g^–1^ in **P3Qy-2** and **P3Qy-2RC**, respectively, that is, showing a further improvement probably due to the presence of extra triazine moieties. In the literature, few COF examples are reported for benzene or cyclohexane adsorption, and we compared those with our MCR-COFs (Table S5). It was observed that the overall uptake is lower and comparable with lower pressure (*p*/*p*_0_ ∼ 0.1).^19^ However, the overall uptake is higher than macromolecules or some metal organic framework systems.^19^

Furthermore, the interaction of Bz/Cy was monitored by fluorescence spectroscopy by measuring the emission wavelength and intensity. A red shift and lowering of the emission intensity were observed for Bz compared to Cy, attributed to the interaction of benzene with the pendant phenyl group in the pore of **P3Qy-1** and **P3Qy-1RC** (Figure S38).^19^ In the case of **P3Qy-1RC** a characteristic shoulder peak was observed, indicating the nature of the fuse ring structure. However, in the case of **Im-1,** the overall change of the emission for Bz/Cy is not significant.

Based on sorption ability and efficient interaction with Bz, we further monitor the selective uptake of the Bz/Cy mixture by a time-dependent solid–vapor sorption experiment and Monte Carlo simulation. **P3Qy-1** and **Im-1** were taken for selective vapor sorption from an equimolar Bz/Cy mixture (1:1) over time (Figures 6C and S39). A higher selectivity of Bz over Cy was observed for **P3Qy-1** compared to **Im-1**. The uptake of Bz was almost saturated after 1 h thus following the trend seen in the single-component sorption experiment. In practical applications, the recyclability of the adsorbent is important. Therefore, both **P3Qy-1** and **Im-1** were washed with acetone after 3 h of sorption and then dried in an oven at 80 °C to reactivate the COFs. We carried out the process for 10 cycles, and after that, we measured ^1^H NMR, PXRD, and Ar sorption at 87 K (Figure S40). Obviously, **P3Qy-1** maintains its sorption capability and structural integrity; however, in the case of **Im-1** both deteriorated.

The presence of pendant phenyl groups within the pores of MCR-COF leads to smaller pore sizes compared to **Im-1**. This structural feature enables selective diffusion of Bz over Cy (larger size compared to Bz). On the other hand, the uptake of Bz in **P3Qy-1** is higher than that in **P4Qy-1** due to the orientation of the pendant phenyl ring within the pore. In **P3Qy-1**, the pendant phenyl ring is more tilted, leading to a greater interlayer distance. This orientation facilitates additional uptake through both edge-to-face T-shaped and parallel-displaced π–π stacking of Bz, as well as H-bonding interactions with the triazine nitrogen. In contrast, the 4-positioned benzene in **P4Qy-1**, which is less tilted, primarily engages in edge-to-face T-shaped π–π stacking Monte Carlo simulations shed light on such an observation and showed selective Bz uptake over Cy for all COFs, and among them all MCR-COFs show a better separation of Bz over **Im-1** due to smaller pore size (Figure S41). The simulated results were comparable with the above time-dependent solid–vapor sorption experiment. From the simulation, both C–H(COF)•••π (Bz) [with a distance of 2.32–3.0 Å], C–H(Bz)•••N (triazine and quinoline) [distance of 3.48–3.6 Å] with COF and Bz, and Bz-Bz interactions were observed for all COFs (Figures 6D and S42). In **P3Qy-1** and **P3Qy-2**, the pendant phenyl ring is more tilted with a greater interlayer distance allowing for an additional parallel-displaced π–π stacking of benzene (Bz) at a distance of 3.14–3.21 Å. This additional characteristic enables **P3Qy** to adsorb more benzene compared with analogous COFs.

## Conclusions

In summary, we have prepared a novel family of multicomponent COFs via one-pot domino and in situ domino Scholl reactions and applied them for industrially important chemical separations. The domino reaction yielded a novel 2,3-phenyl-quinoline-linked COF (**P3Qy**) in contrast to the known Povarov reaction, which yields 2,4-phenyl-quinoline-linked COFs (**P4Qy**). This is achieved by changing the third component in the multicomponent reaction from styrene to epoxystyrene. The special feature of 2,3-phenyl-quinoline is that it can undergo an in situ Scholl reaction to create heteropolyaromatic COFs. These COFs were furthermore compared to their imine-linked counterpart.

This new family of MCR-COFs showed high crystallinity and stability in strong acids and bases in contrast to the respective imine-COF. The pendant phenyl groups within the COF pore play a crucial role in the diffusion of Bz and allow strong interaction which facilitates efficient sorption and separation over cyclohexane which is a challenge of petrochemical engineering. This work shows the development of several new stable and functional MCR-COFs achieved through meticulous control of their structural and compositional parameters. These advancements hold significant promise for diverse applications in various fields.

## Supporting Information Available

Experimental details and additional characterization (ScXRD, PXRD, Powley refinement, NMR, FTIR, XPS, TGA, FESEM, HRTEM, solid-state UV/vis spectra, and detailed solvent sorption experiment and simulation) (PDF)
