# Supplementary material for: Heteropolyaromatic Covalent Organic Frameworks via One-Pot Multicomponent Reactions
Source: J Am Chem Soc. 2024 Jun 14;146(25):17131–9. doi: 10.1021/jacs.4c02551 (PMC11212053; doi:10.1021/jacs.4c02551)
Supplement: Supplementary file 1 — ja4c02551_si_001.pdf [file ja4c02551_si_001.pdf]

Supporting Information

Heteropolyaromatic Covalent Organic Frameworks via One-Pot Multicomponent Reactions

Prasenjit Das,*† Gouri Chakraborty,# Nico Friese,† Jérôme Roeser,† Carsten Prinz# Franziska Emmerling#, Johannes Schmidt,† and Arne Thomas*,†

†Department of Chemistry/Functional Materials, Technische Universität Berlin, 10623 Berlin, Germany

#BAM Federal Institute for Materials Research and Testing, Richard-Willstätter-Str. 11, 12489 Berlin, Germany

E-mail: arne.thomas@tu-berlin.de

E-mail: prasenjitsepistles@gmail.com

Table of Contents

Section	Description	Page No
S1	General Materials and Methods	S-3 to S-5
S2	Synthesis of Model compound and COFs (Scheme S1-S7, Figures S1-S4, Table S1)	S-6 to S-15
S3	Structural Modelling and Atomic Coordinates of COFs (Figures S5-S10 and Tables S2-S4)	S-16 to S-25
S4	Structure Characterization of MCR-COFs (Figures S11-S13)	S-26 to S-28
S5	FESEM and HRTEM Images of COFs (Figures S14-S23)	S-29 to S-34
S6	Chemical and Thermal Stability of MCR-COFs (Figures S24-S29)	S-35 to S-38
S7	Stepwise Synthesis of Novel MCR-COF (Figures S30-S32)	S-39 to S-40
S8	UV-vis Spectra and Band Structure of MCR-COFs (Figures S33-S36)	S-41 to S-42
S9	Benzene (Bz) and Cyclohexane (Cy) Sorption and Separation by COFs (Figures S37-S42, Table S5)	S-43 to S-51

Section S1. General Materials and Methods

Materials: All purchased chemicals were used without further purification except where otherwise noted. *o*-DCB (1,2-Dichlorobenzene, 99%) anhydrous *n*-BuOH (*n*-Butanol, 99%), epoxy styrene, styrene, 2,3-Dichloro-5,6-dicyano-1,4-benzoquinone (DDQ), boron trifluoride diethyl etherate ($BF_3 \cdot Et_2O$), scandium trifluoromethanesulfonate ($Sc(OTf)_3$) and copper trifluoromethanesulfonate ($Cu(OTf)_2$) was purchased from Sigma Aldrich Chemicals. 2,4,6-Tris(4-aminophenyl)-1,3,5-triazine, (95%), 2,4,6-Tris(4-formylphenyl)-1,3,5-triazine, (>96.0%), and 1,3,5-Tris(4-aminophenyl)benzene were all supplied by BLDPharm Germany. The synthetic Grade Solvents were purchased from Carl Roth.

^{1}H-NMR and ^{13}C-NMR spectra were recorded on a Bruker Avance II 200 MHz spectrometer in the given solvent. Data are reported in the following order: chemical shift (δ) in ppm; multiplicities (br for broadened singlet, s for singlet, d for doublet, t for triplet, m for multiplet); coupling constants (J) in Hertz (Hz); number of protons. ^{13}C Solid-state NMR (cross polarization magic-angle spinning (CP/MAS)) spectra were carried out on a Bruker Avance 400 MHz spectrometer operating at 100.6 MHz.

Thermogravimetric Analyses (TGA) were performed using a TGA Q500 thermal analysis system under a N_2 and air atmosphere from room temperature to 800 °C at a ramping rate of 2 °C /min.

Attenuated Total Reflectance Fourier-Transform Infrared Spectrometry (ATR-FT-IR) was conducted using a PerkinElmer Spectrum Two spectrometer with diamond/ZnSe ATR accessory. All spectra were collected using a $LiTaO_3$ MIR detector over a range of 450 to 4000 cm^{-1}. All spectra were processed using Spectrum 10 software.

Solid-State Diffuse Reflectance Ultraviolet–visible Spectroscopy (UV-vis) spectra of the pristine COF powders and starting monomers have been collected on Varian Cary 300 UV-vis Spectrophotometer.

Ar Sorption Measurement was performed at 87 K using an Autosorb-iQ-MP from Quantachrome. Prior to the analysis the sample was activated at 120 ^{o}C for 24 h. Using the Ar adsorption isotherm, the surface area was calculated over a pressure range 0.05-0.1 = p/p_0 using Brunauer-Emmett-Teller (BET) methods.

Field Emission Scanning Electron Microscopy (FESEM) was measured on a ZEISS GeminiSEM500. All COFs was observed directly without gold coating in nanoVP mode.

High Resolution Transmission Electron Microscopy (HRTEM) images were measured in JEOL G-ARM STEM (JEM ARM300F2). All the COF samples were prepared in carbon grid after suspension in EtOH-toluene mixture.

Section S2. Synthesis of Model Compound and COFs

Synthesis of Model Compound: 6,7-Dimethoxy-3-phenyl-2-*(4-fluorophenyl)*quinoline (PE-1)

A 25 mL round bottom flask was charged with 3,4-dimethoxyaniline (150 mg, 1 mmol), 4-fluorobenzaldehyde (107 μL, 1 mmol), styrene oxide (111 μL, 1 mmol) and $Cu(OTf)_2$ (32 mg, 1 mmol). DMSO (1 mL) was added and the mixture was stirred for 4 h at 80 ˚C. The flask was cooled to ambient temperature and ethyl acetate (5 mL) was added. The solution was transferred to a separation funnel and deionized water (30 mL) was added. The aqueous phase was extracted with ethyl acetate (3 × 20 mL), the combined organic layers were washed with brine, dried over magnesium sulfate, filtrated and all volatile compounds were removed under reduced pressure. The crude product was further purified with flash chromatography (*n*-hexane/ethyl acetate = 10:1, 20 mL) to yield light-yellow crystals (92 mg, 61%). Finally, the crystal was utilized for characterization. ^{1}H NMR (200 MHz, $CDCl_3$): δ 3.78 (s, 3H, OCH_3), 3.99 (s, 3H, OCH_3), 6.80 (m_c, 1H, Ar-H), 7.01 (dd, 1H, J = 8 Hz, Ar-H), 7.06 (s, 1H, Ar-H), 7.18 (s, 1H, Ar), 7.26 (m_c, 1H, Ar-H), 7.33 (s, 1H, Ar-H), 7.48 (m, 5H, Ar-H), 7.74 (s, 1H, Ar-H), 7.94 (s, 1H, Ar-H). ^{13}C NMR (50 MHz, $CDCl_3$): δ 56.0, 56.3, 103.7, 106.7, 116.0, 118.5, 123.2, 127.6, 128.7, 129.6, 131.8, 132.2, 136.5, 137.3, 140.1, 144.1, 150.2, 153.1, 155.5, 161.1. HRMS (ESI) *m*/*z* calculated for $[M + H]^+$: 360.1355, found 360.1393.

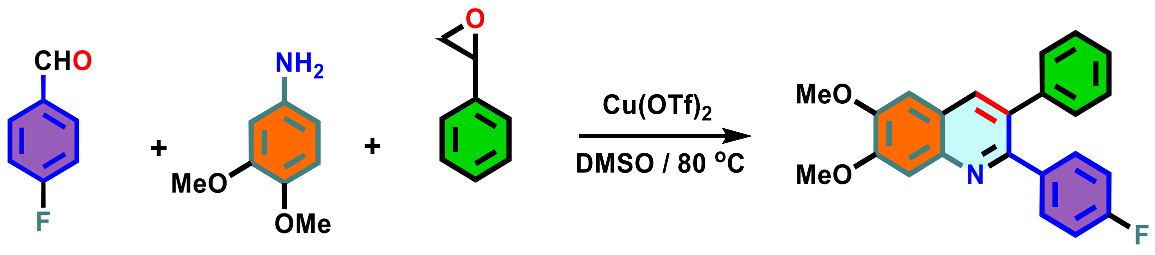

Scheme S1. Synthesis of the model compound (**PE-1**).

Synthesis of Model Compound: One Pot Scholl Reaction (PE-1RC)

A 25 mL round bottom flask was charged with 3,4-dimethoxyaniline (150 mg, 1 mmol), 4-fluorobenzaldehyde (107 μL, 1 mmol), styrene oxide (111 μL, 1 mmol), DDQ (20 mg), $Cu(OTf)_2$ (32 mg, 1 mmol), and DMSO (1 mL) was added and the mixture was stirred for 10 h at 100 ˚C. The flask was cooled to ambient temperature and ethyl acetate (5 mL) was added. The solution was transferred to a separation funnel and deionized water (30 mL) was added. The aqueous phase was extracted with ethyl acetate (3 × 20 mL), the combined organic layers were washed with brine, dried over magnesium sulfate, filtrated and all volatile compounds were removed under reduced pressure. The crude product was further purified by column chromatography (*n*-hexane/ethyl acetate = 10:1, 20 mL) to yield a light brown product. HRMS (ESI) *m*/*z* calculated for $[M + H]^+$: 358.1199, found 358.1230.

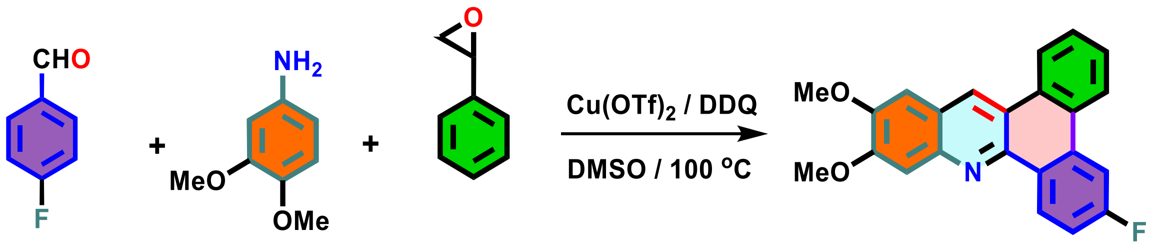

Scheme S2. Synthesis of the model compound (**PE-1RC**).

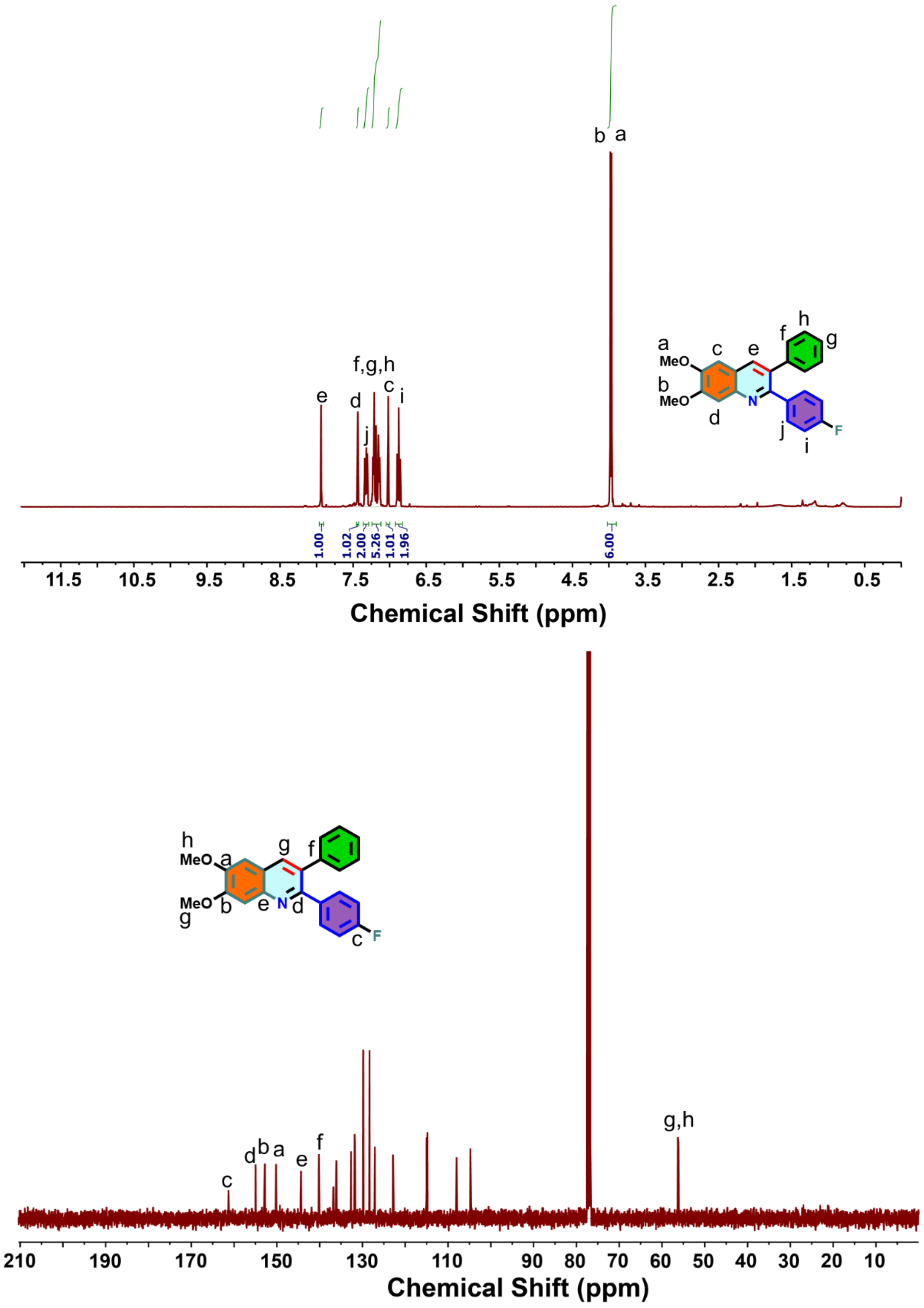

Figure S1. ^{1}H NMR (top) and ^{13}C NMR (bottom) spectra of the model compound in DMSO-d$_6$ (unmark peak belong to all benzene carbon).

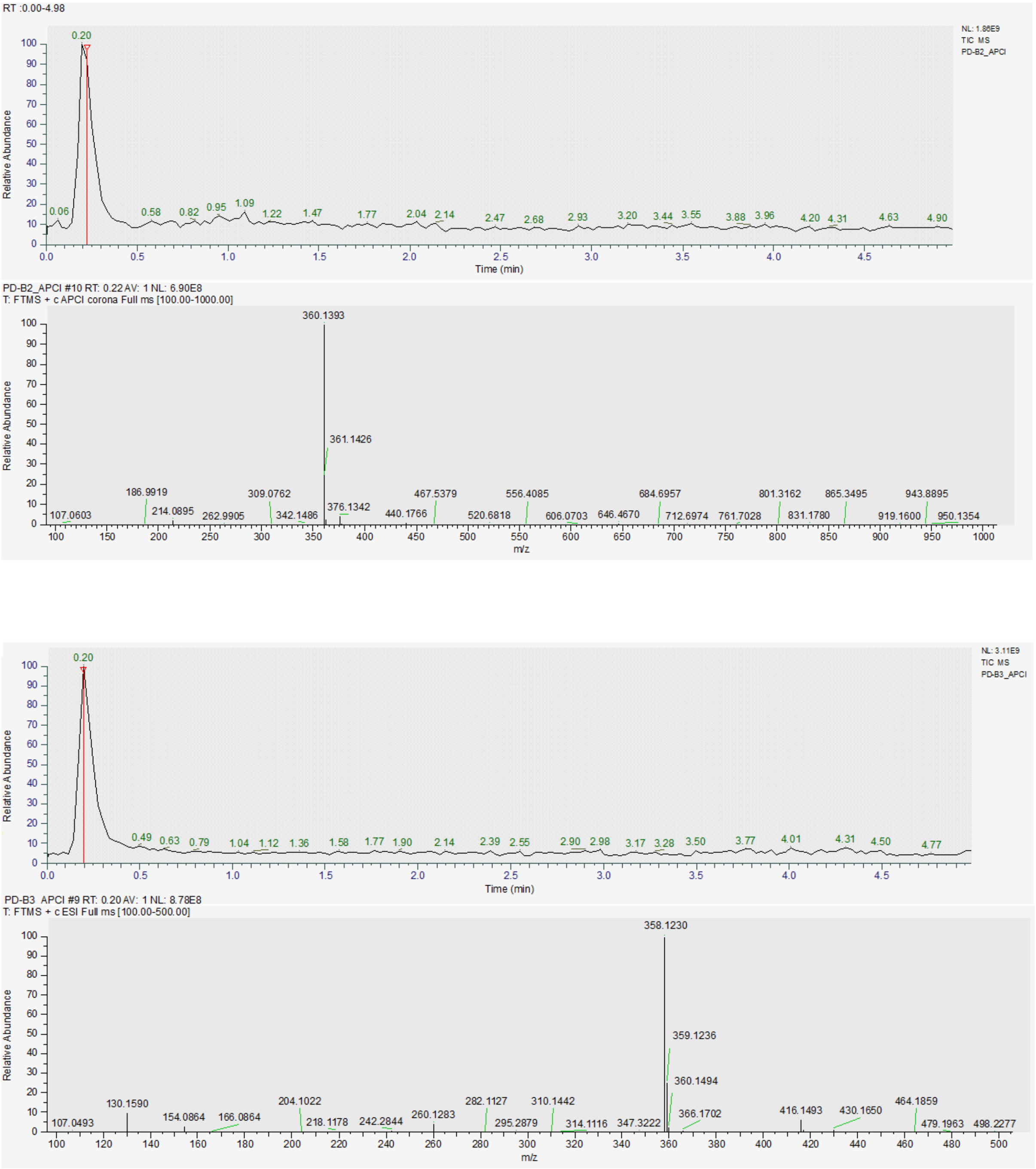

Figure S2. HRMS spectra of model compound PE-1[M+H: 360.1355] (top) and PE-1RC [M+H: 358.1198] (bottom).

Single crystal X-ray diffraction (SXRD) analysis of linker and model compound: Using an optical microscope, one suitable crystal of **PE-1** was put inside a nylon loop attached to a goniometer head, which was then placed under a cold stream of nitrogen gas for slow cooling to 150 K. On the basis of the crystal suitability from diffraction photographs and unit cell determination, data were collected on an Oxford Diffraction XCalibur (4 Circle Diffractometer, CCD Detector) from Rigaku oxford diffraction.

Table S1. Crystallographic data and structure refinement parameters for the **Model compound**.

compound	**PE-1**
CCDC No.	2308605
chemical formula	$C_{23}H_{18}FNO_2$
formula weight (g mol^{-1})	359.403
temperature (K)	150.00(10)
wavelength (Å)	0.71073
crystal system	triclinic
space group	*P-1*
a (Å)	9.1625(8)
b (Å)	17.4383(13)
c (Å)	18.0843(10)
α (°)	108.681(6)
β (°)	95.924(6)
γ (°)	93.425(7)
Z	6
V (Å^3)	2709.4(4)
density (g/cm^3)	1.475
μ (mm^{-1})	0.744
F(000)	1131.768
2θ (°) range for data collection	2.6480 to 71.1900
no. of reflections collected	2784
no. of independent reflections	2490
no. of reflections with $I > 2\sigma(I)$)	20481
$R_{\rm int}$	0.728
no. of parameters refined	731
GOF on F^2	1.0797
final $R_1{}^a$ / w$R_2{}^b$ (I >2σ(I))	0.0806/0.2223
$R_1{}^a$ / w$R_2{}^b$ (all data)	0.1412/0.2836
largest diff. peak and hole (e Å^{-3})	2.7069/-0.9708

$^aR_1 = \Sigma||F_o| - |F_c||/\Sigma|F_o|$. ^{b}w$R_2 = [\Sigma w(F_o{}^2 - F_c{}^2)^2/\Sigma w(F_o{}^2)^2]^{1/2}$, where $w = 1/[\sigma^2(F_o{}^2) + (aP)^2 + bP]$, $P = (F_o{}^2 + 2F_c{}^2)/3$.

Synthesis of P3Qy-1

A Pyrex glass tube (15 mL) was charged with 1,3,5-Tris(4-aminophenyl)benzene (TAB) (54 mg, 0.15 mmol), 4,4',4''-(1,3,5-triazine-2,4,6-triyl)tribenzaldehyde (TTA) (60 mg, 0.15 mmol), styrene oxide (75 μL, 0.6 mmol), DDQ (5 mg), $Sc(OTf)_3$ (20 mg, 0.06 mmol) and acetic acid (50 μL, 6 M) in *o*-dichlorobenzene (*o*-DCB)/*n*-BuOH (2/2 mL). The tube was first sonicated for 20 minutes to form bulk solid and then flash frozen at 77 K (liquid N_2 bath) and degassed by three times of freeze-pump-thaw cycles. The internal pressure was evacuated to 10^{-3} mbar. The tube was sealed and heated at 120 °C for 3 days. The bright golden yellow precipitate was washed with mixture of solvents (MeOH, acetone) several times and collected after Soxhlet extraction for 12 h. Finally, the powder was dried at 80 °C for 4h. Yield = 75% (112 mg). The elemental analysis of **P3Qy-1** found to be (%): C, 84.49; H, 4.65; N, 10.24, are close with calculated value (%): C, 87.25; H, 4.27; N, 8.48.

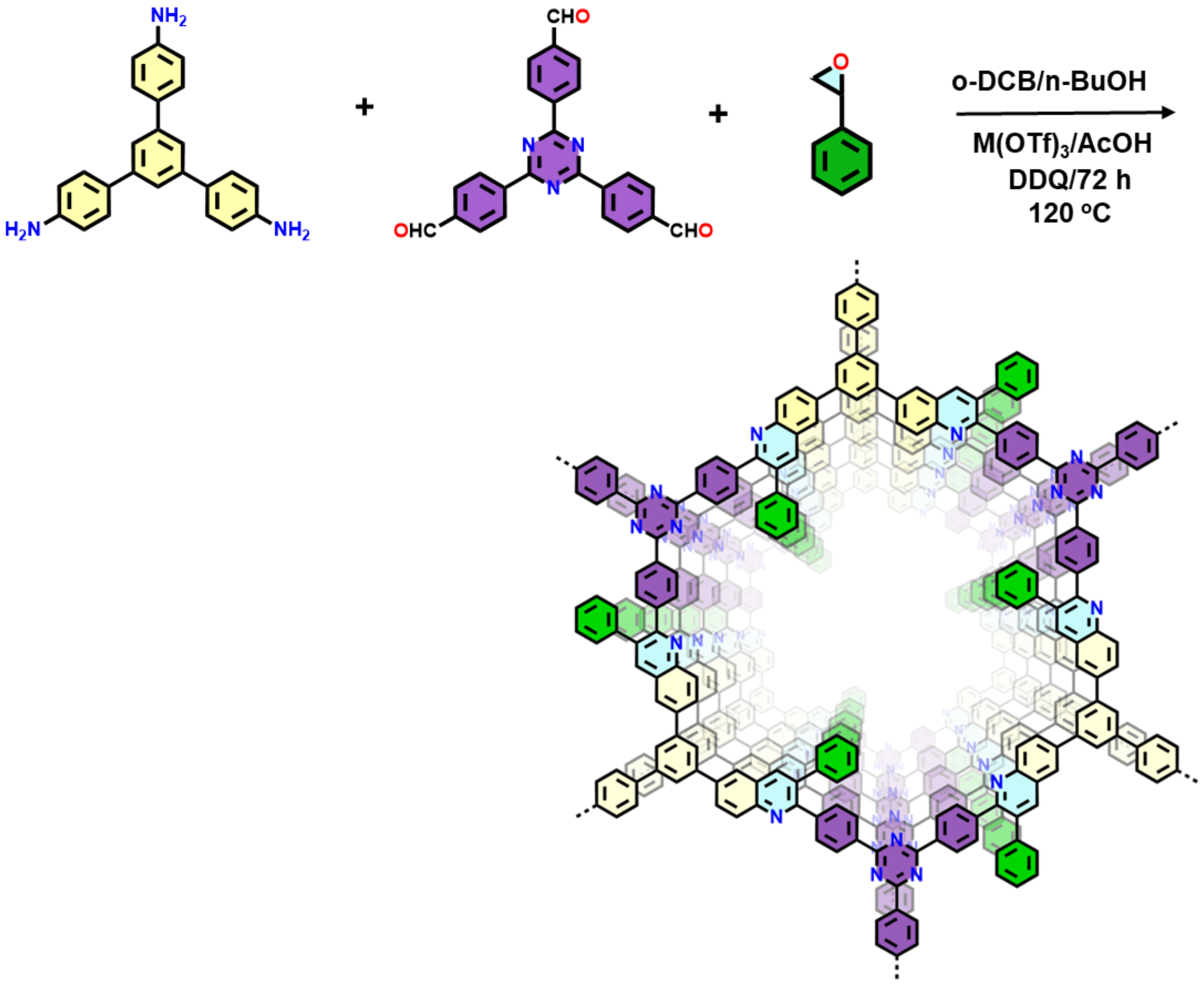

Scheme S3. Synthesis of **P3Qy-1**.

Synthesis of Im-1

A Pyrex glass tube (15 mL) was charged with TAB (54 mg, 0.15 mmol), TTA (60 mg, 0.17 mmol), (*o*-DCB)/*n*-BuOH (2/2 mL) and 0.2 mL 6 M acetic acid aqueous solution. The tube was first sonicated for 20 minutes and then flash frozen at 77 K (liquid N_2 bath) and degassed by three times of freeze-pump thaw cycles. The internal pressure was evacuated to 10^{-3} mbar. The tube was sealed and placed in a preheated oven at 120 °C for 3 days. After finishing heating, the tube was cooled down and cut. The formed yellow precipitate was filtered and washed with acetone/MeOH several times. Finally, the powder was dried at 80 °C for 4 h. Yield = 88 % (102 mg). Anal. Calcd (%):C, 76.57; H, 4.75; N, 17.86. Found (%): C, 75.55; H, 4.12; N, 17.02.

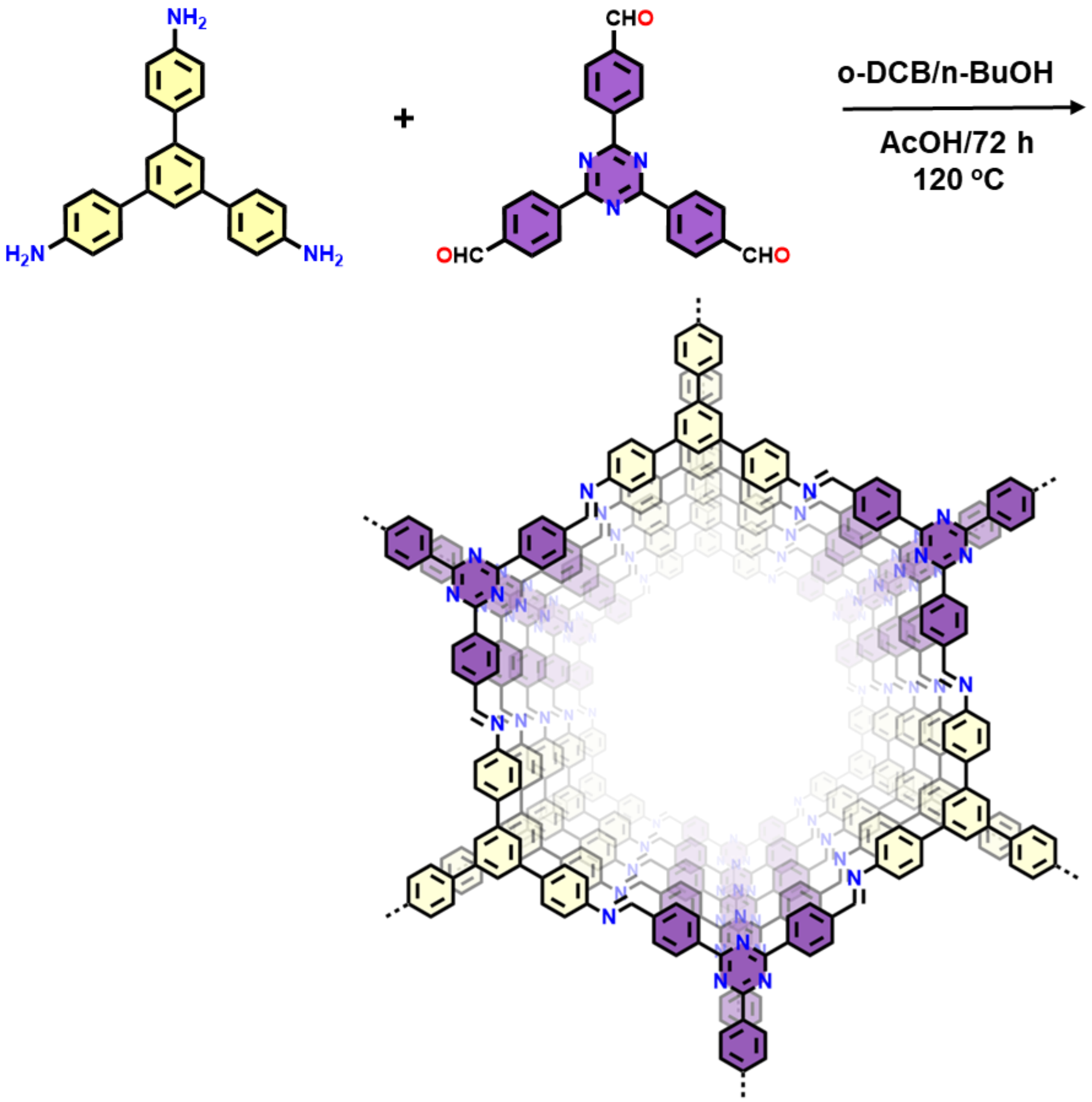

Scheme S4. Synthesis of **Im-1.**

Synthesis of P4Qy-1

A Pyrex glass tube (15 mL) was charged with TAB (54 mg, 0.15 mmol), TTA (60 mg, 0.15 mmol), styrene (60 μL, 0.6 mmol), DDQ (5 mg), $BF_3 \cdot Et_2O$ (1 drop) and acetic acid (100 μL, 6 M) in *o*-DCB/*n*-BuOH (2/2 mL). The tube was first sonicated for 20 minutes to form bulk solid and then flash frozen at 77 K (liquid N_2 bath) and degassed by three times of freeze-pump-thaw cycles. The internal pressure was evacuated to 10^{-3} mbar. The tube was sealed and heated at 120 °C for 3 days. The yellow precipitate was washed with mixture of solvents (MeOH, acetone) several times and collected after Soxhlet extraction for 12 h. Finally, the powder was dried at 80 °C for 4 h. Yield = 75% (108 mg). The elemental analysis of **P4Qy-1** found to be (%): C, 79.64; H, 4.52; N, 13.95, are close with calculated value (%): C, 83.36; H, 3.95; N, 12.68.

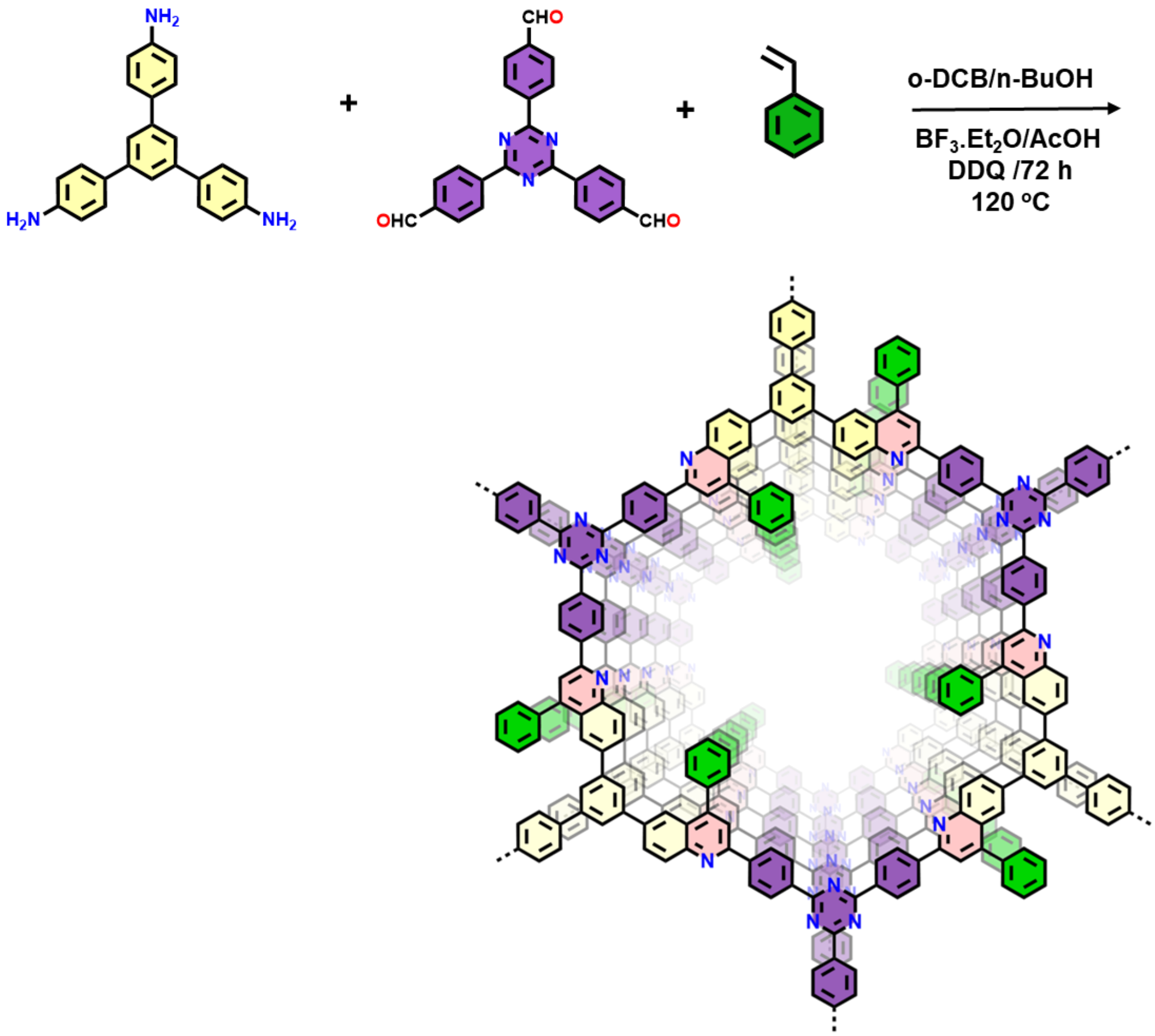

Scheme S5. Synthesis of **P4Qy-1.**

Synthesis of P3Qy-1RC

A Pyrex glass tube (15 mL) was charged with TAB (54 mg, 0.15 mmol), TTA (60 mg, 0.15 mmol), styrene oxide (75 µL, 0.6 mmol), DDQ (5 mg), $Sc(OTf)_3$ (20 mg, 0.06 mmol) and acetic acid (50 µL, 6 M) in *o*-DCB/*n*-BuOH (2/2 mL). The tube was first sonicated for 20 minutes to form bulk solid and then flash frozen at 77 K (liquid N_2 bath) and degassed by three times of freeze-pump-thaw cycles. The internal pressure was evacuated to 10^{-3} mbar. The tube was sealed and heated at 140 °C for 3 days. The greenish yellow precipitate was washed with mixture of solvent several times and collected after Soxhlet extraction. Finally, the powder was dried at 80 °C for 4h. Yield = 70% (98 mg). The elemental analysis of **P3Qy-1RC** found to be (%): C, 84.75; H, 4.62; N, 10.24, are close with calculated value (%): C, 87.79; H, 3.68; N, 8.53. N.B. if the same reaction was performed with an increase amount of DDQ (25 mg) at 120 °C the formation of **P3Qy-1RC** was achieved and the color remained consistent.

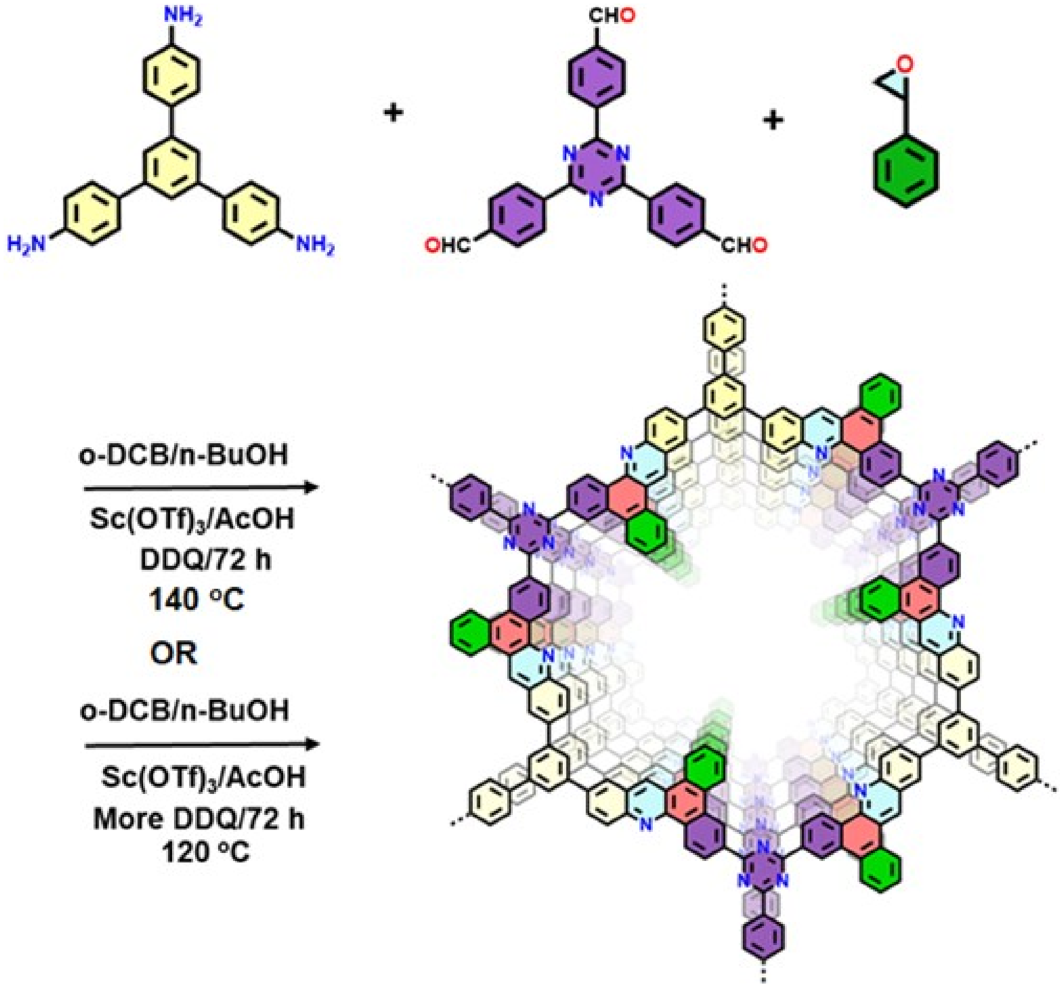

Scheme S6. Synthesis of **P3Qy-1RC.**

Synthesis of P3Qy-2

A Pyrex glass tube (15 mL) was charged with 2,4,6-Tris(4-aminophenyl)triazine (TAT) (56 mg, 0.16 mmol), TTA (60 mg, 0.16 mmol), styrene oxide (75 μL, 0.65 mmol), DDQ (6 mg), $Sc(OTf)_3$ (10 mg, 0.02 mmol) and acetic acid (50 μL, 6 M) in *o*-DCB/*n*-BuOH (2/2 mL). The tube was first sonicated for 20 minutes to form bulk solid and then flash frozen at 77 K (liquid N_2 bath) and degassed by three times of freeze-pump-thaw cycles. The internal pressure was evacuated to 10^{-3} mbar. The tube was sealed and heated at 100-120 °C for 3 days. The bright golden yellow precipitate was washed with mixture of solvent several times and collected after Soxhlet extraction. Finally, the powder was dried in a normal oven at 80 °C. Yield = 75% (116 mg). The elemental analysis of **P3Qy-2** found to be (%): C, 81.36; H, 4.16; N, 13.45, are close with calculated value (%): C, 83.36; H, 3.95; N, 12.68.

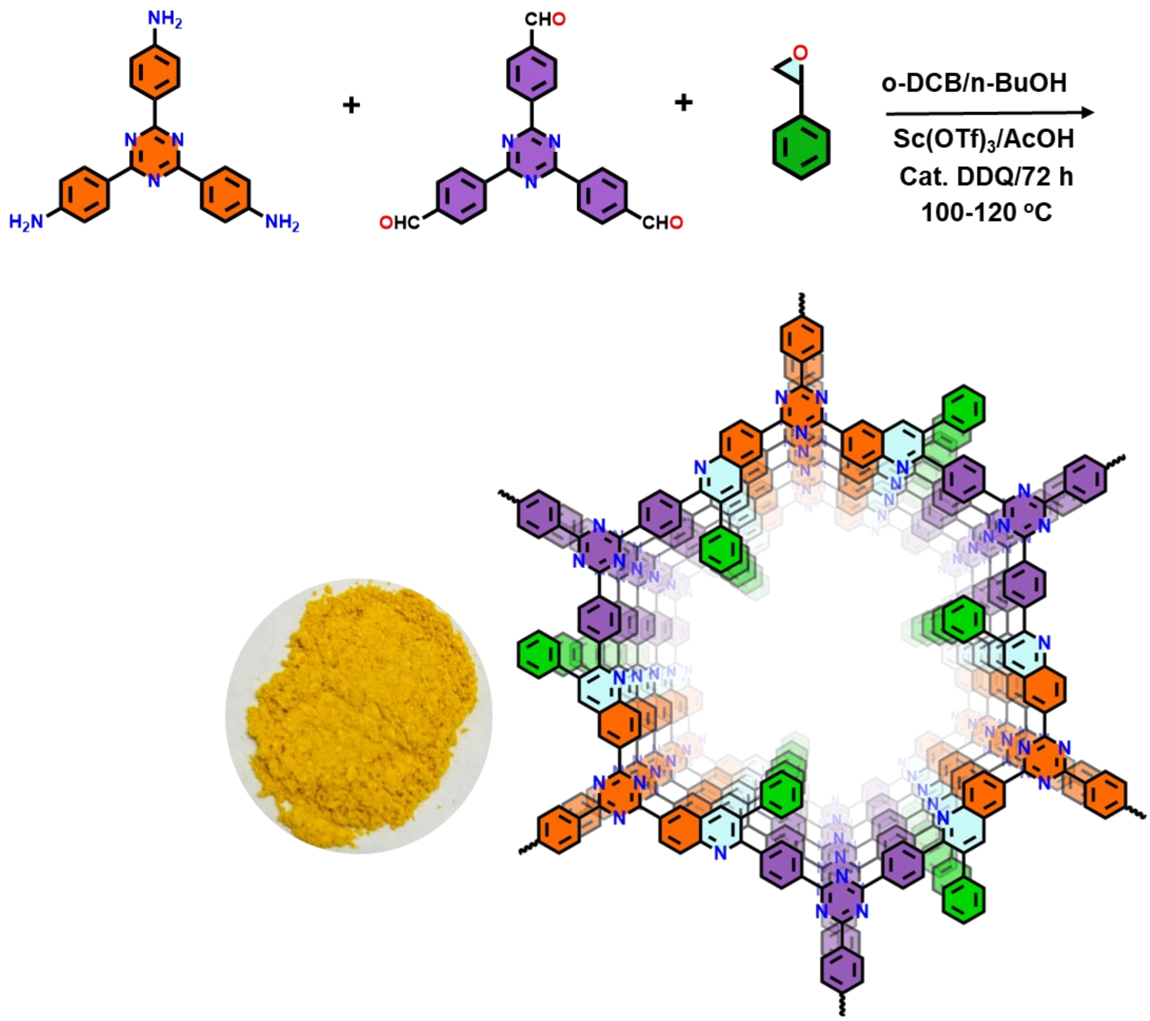

Scheme S7. Synthesis of **P3Qy-2.**

Synthesis of P3Qy-2RC

A Pyrex glass tube (15 mL) was charged with TAT (56 mg, 0.15 mmol), TTA (60 mg, 0.15 mmol), styrene oxide (75 µL, 0.6 mmol), DDQ (5 mg), $Sc(OTf)_3$ (10 mg, 0.06 mmol) and acetic acid (50 µL, 6 M) in *o*-DCB/*n*-BuOH (2/2 mL). The tube was first sonicated for 20 minutes to form bulk solid and then flash frozen at 77 K (liquid N_2 bath) and degassed by three times of freeze-pump-thaw cycles. The internal pressure was evacuated to 10^{-3} mbar. The tube was sealed and heated at 150 °C for 3 days. The green yellow precipitate was washed with mixture of solvent several times and collected after Soxhlet extraction. Finally, the powder was dried in a normal oven at 80 °C. Yield = 70% (102 mg). The elemental analysis of **P3Qy-2RC** found to be (%): C, 81.94; H, 4.13; N, 13.77, are close with calculated value (%): C, 83.88; H, 3.37; N, 12.76. N.B. if the same reaction was performed with an increase amount of DDQ (25 mg) at 120 °C the formation of **P3Qy-2RC** was achieved and the color remained consistent.

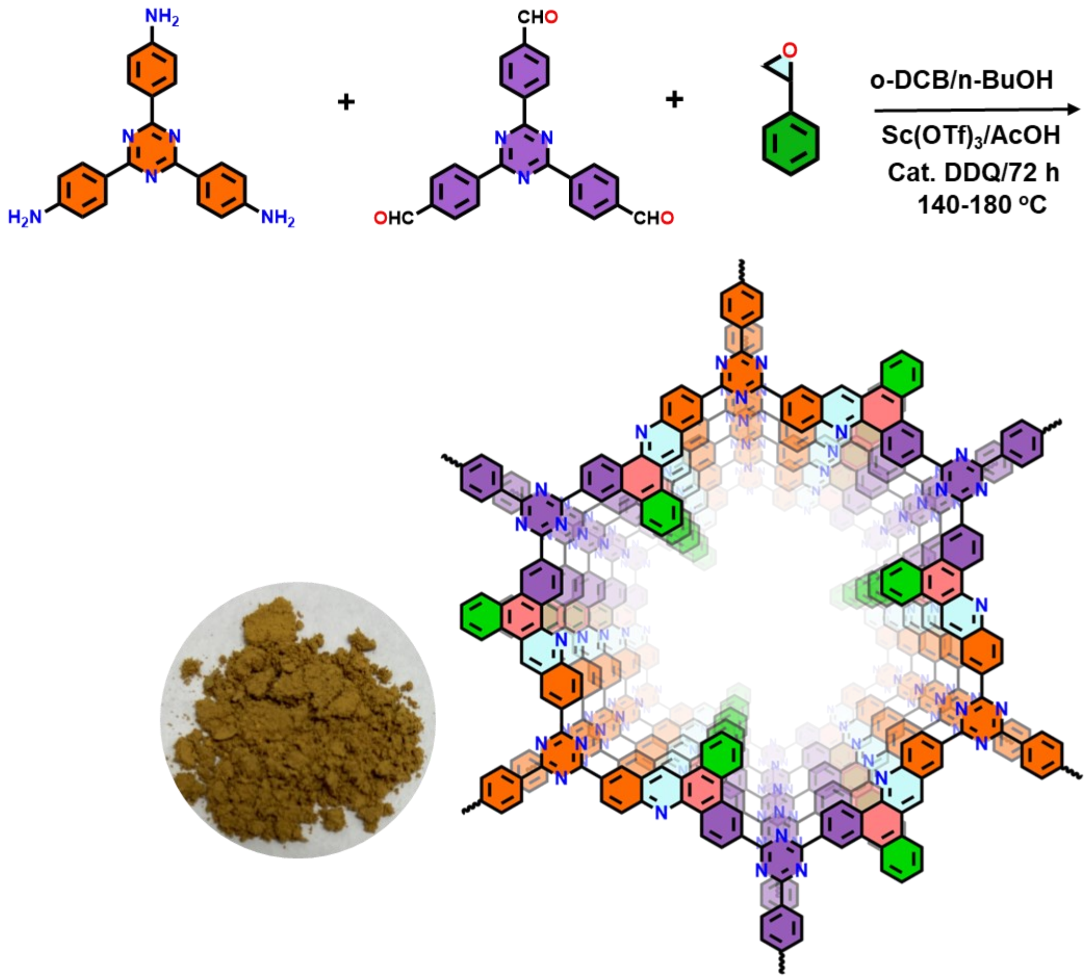

Scheme S8. Synthesis of **P3Qy-2RC.**

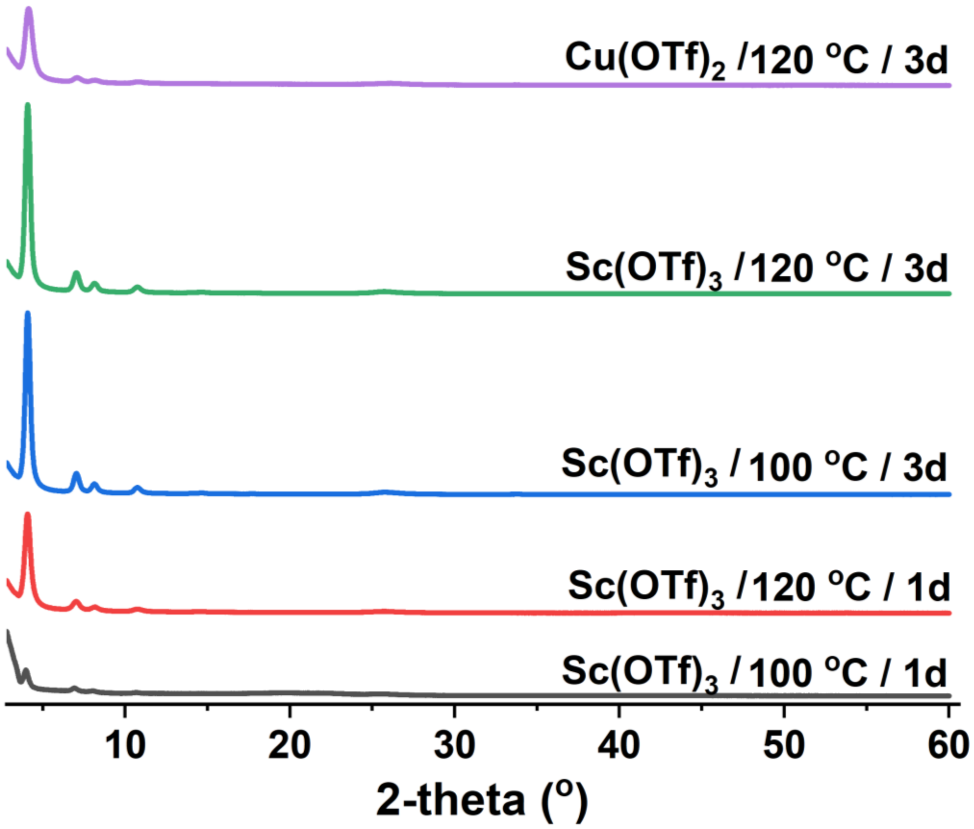

Figure S3. Optimized reaction condition for **P3Qy** COF monitored by PXRD.

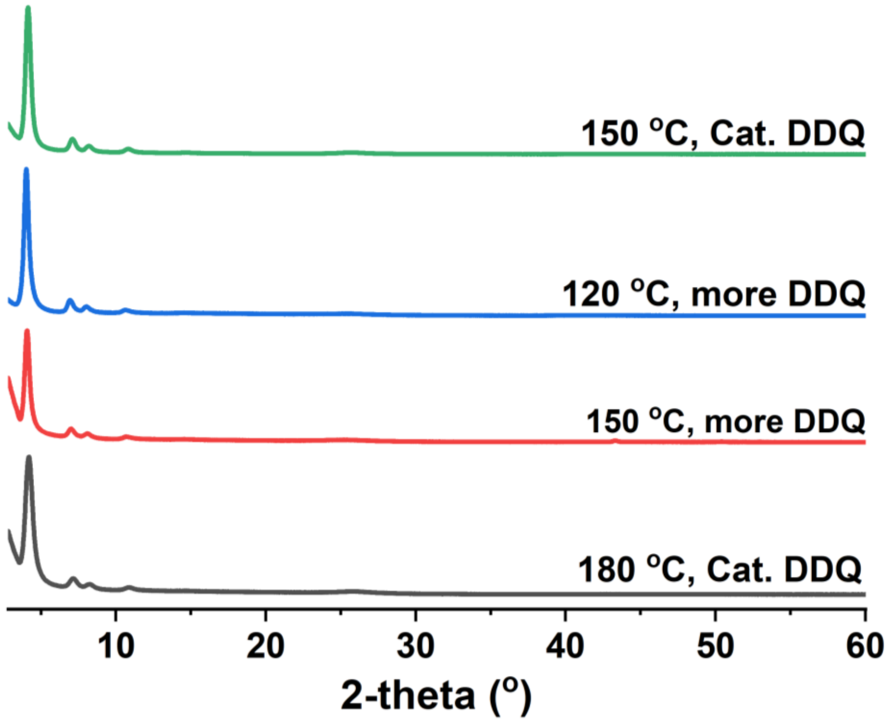

Figure S4. Optimized reaction condition for **P3QyRC** COF monitored by PXRD.

Table S2. Optimized reaction condition screen for P3Qy

Entry	Compounds	Reaction condition			Crystallinity
		Solvent	Temperature	Catalyst	
1	**P3Qy**	DCB/n-BuOH	120 °C, 3 days	$Sc(OTf)_3$, Cat. DDQ	high
2		DCB/n-BuOH	120 °C, 1 days	$Sc(OTf)_3$, Cat. DDQ	Moderate high
3		DCB/n-BuOH	120 °C, 3 days	$Cu(OTf)_3$, Cat. DDQ	High
4		DCB/n-BuOH	100 °C, 3 days	$Sc(OTf)_3$, Cat. DDQ	High
5		DCB/n-BuOH	80 °C, 3 days	$Sc(OTf)_3$, Cat. DDQ	Moderate high
6		DCB/n-BuOH	100 °C, 1 days	$Sc(OTf)_3$, Cat. DDQ	Moderate
7		Mestylene/dioxane	120 °C, 3 days	$Sc(OTf)_3$, Cat. DDQ	Moderate high
8	**Im-1** to **P3Qy-1**	DCB/n-BuOH	120 °C, 3 days	$Sc(OTf)_3$, Cat. DDQ	Moderate high

Table S3. Optimized reaction condition screen for P3Qy-RC

Entry	Compounds	Reaction condition			Crystallinity
1	**P3QyRC**	DCB/n-BuOH	150-180 °C, 3 days	$Sc(OTf)_3$, Cat. DDQ	high
2		DCB/n-BuOH	120 °C, 3 days	$Sc(OTf)_3$, more DDQ	high
3		DCB/n-BuOH	150 °C, 3 days	$Cu(OTf)_3$, cat. DDQ	High
4		DCB/n-BuOH	150 °C, 3 days	$Sc(OTf)_3$, more DDQ	Moderate
5	**P3Qy** to **P3Qy-RC**	DCB/n-BuOH	120 °C, 2 days	$Sc(OTf)_3$, more DDQ	Moderate
6	**Im-1** to **P3Qy-1RC**	DCB/n-BuOH	150 °C, 2 days	$Sc(OTf)_3$, Cat. DDQ	Amorphous

Note: Amine (0.15 mmol), Aldehyde (0.15 mmol), styrene oxide (0.6 mmol), cat. DDQ (5 mg), more DDQ (25 mg), $Sc(OTf)_3$ (0.06-0.1 mmol) or $Cu(OTf)_2$ (0.06-0.1 mmol) and acetic acid (50 µL, 6 M) in *o*-DCB/*n*-BuOH (2/2 mL).

The reaction time is important to get high crystallinity because it allows better error correction to make multicomponent COF. If we use high concentration of DDQ and high temperature (>120 °C), the formation of Scholl reaction was observed, however, the crystallinity of the P3QyRC decreases, probably due to a too fast formation of the aromatic quinoline bridge as well as oxidation of two phenyl ring which forms an irreversible strong bond. Therefore, catalytic amount of DDQ is an optimal temperature to get highly crystalline COFs. Here, DDQ use as a versatile and recyclable oxidant.

Section S3. Structural Modelling and Atomic Coordinates of COFs

X-ray Powder Diffraction patterns were collected on a Bruker D8 Advance diffractometer in reflection geometry operating with a Cu Kα anode (λ = 1.54178 Å) operating at 40 kV and 40 mA. Samples were ground and mounted as loose powders onto a Si sample holder. PXRD patterns were collected from 2 to 60 2θ degrees with a step size of 0.01 degrees and an exposure time of 1 second per step.

The structural crystal models with **hcb** topology of all the COFs were initially constructed in hexagonal unit cell in the Materials Studio suite of programs by Accelrys. Geometry optimization of the structures with Universal Force Field (UFF) led to satisfactory models whose theoretical pattern matched well the experimentally obtained patterns in terms of reflection positions and relative intensities. The Pawley profile refinements were performed using a Pseudo-Voigt profile function. The observed diffraction patterns were subjected to a polynomial background subtraction and the refined parameters included the zero-point shift, the unit cell parameters, the FWHM parameters and the peak asymmetry (Berar-Baldizzoni function). For all the COFs, AA stacking and AB stacking models were constructed, and their corresponding PXRD patterns were calculated except. We chose to represent the AA structural model of the COFs in the fully eclipsed configuration (AA_e).

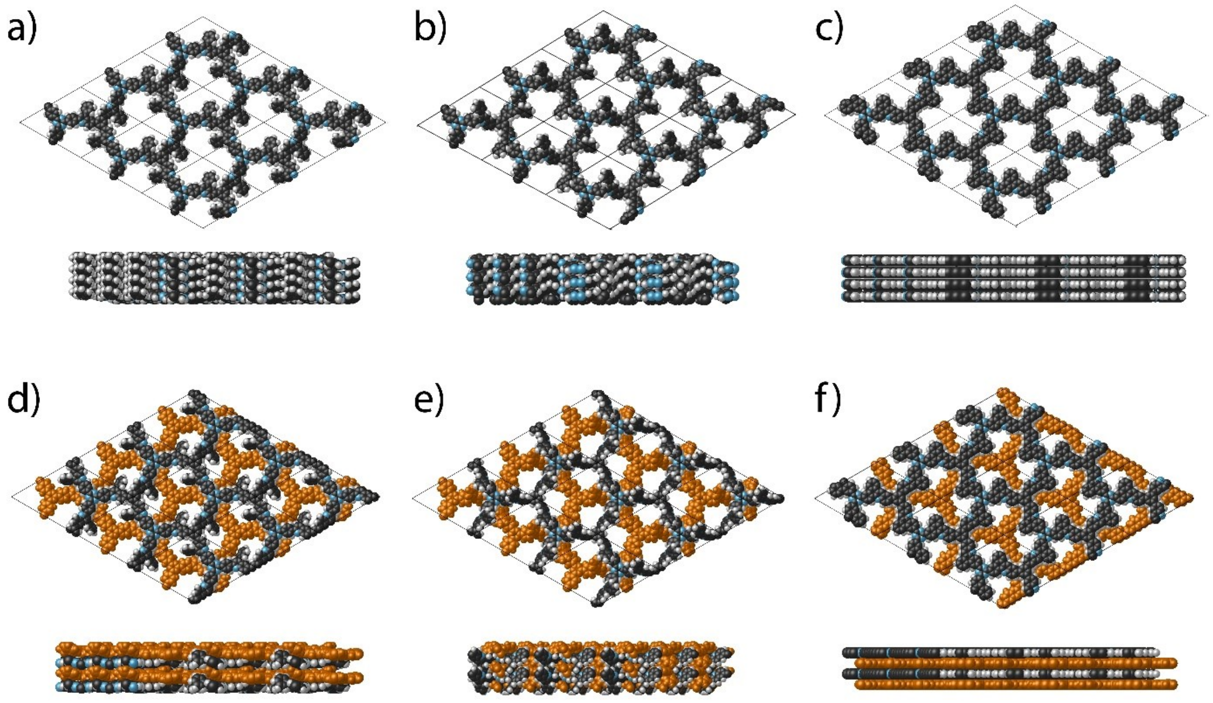

Figure S5. Simulated **hcb** 2D hexagonal layered model with eclipsed (AA, top row) and staggered (AB, bottom row) stacking arrangement of **P4QY-1** (a, d), **P3Qy-1** (b, e) and **P3Qy-1RC** (c, f).

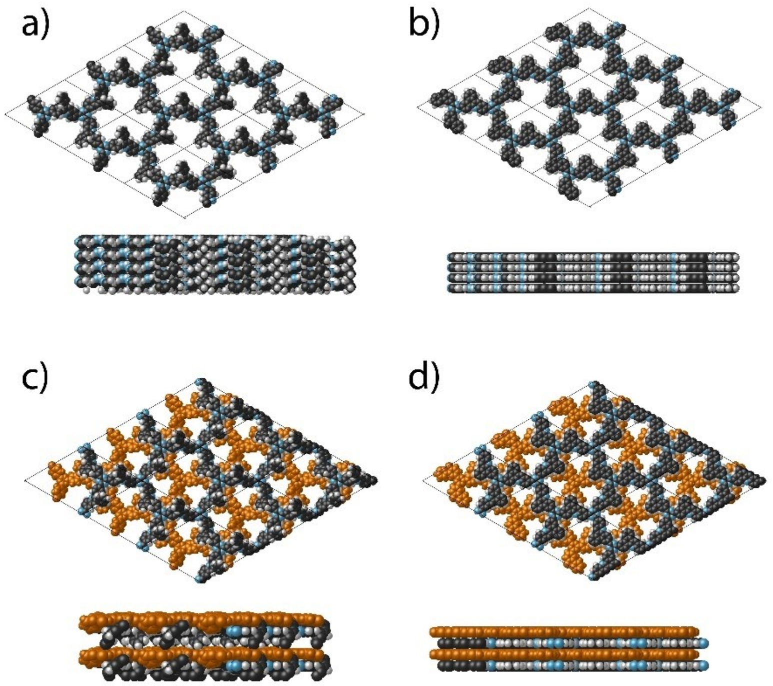

Figure S6. Simulated **hcb** 2D hexagonal layered model with eclipsed (AA, top row) and staggered (AB, bottom row) stacking arrangement of **P3Qy-2** (a, c) and **P3Qy-2RC** (b, d).

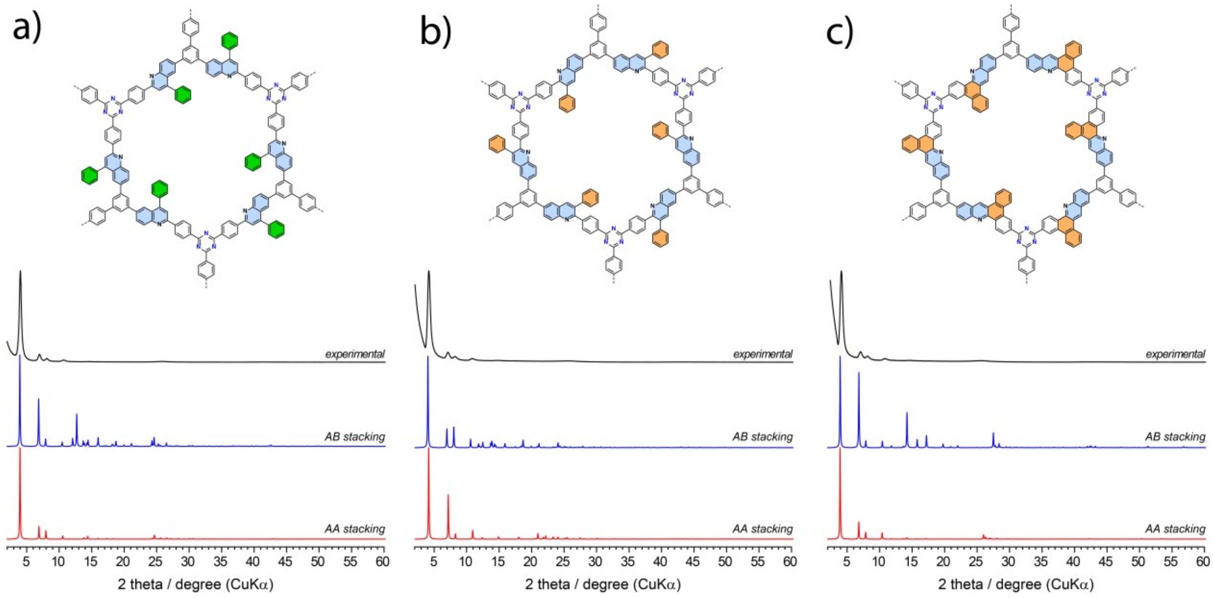

Figure S7. Simulated X-ray diffraction patterns for generated **hcb** hexagonal layered structures adopting fully eclipsed (red) and staggered (blue) stacking arrangement compared to the experimentally obtained patterns (black) of **P4Qy-1** (a) **P3Qy-1** (b) and **P3Qy-1RC** (c).

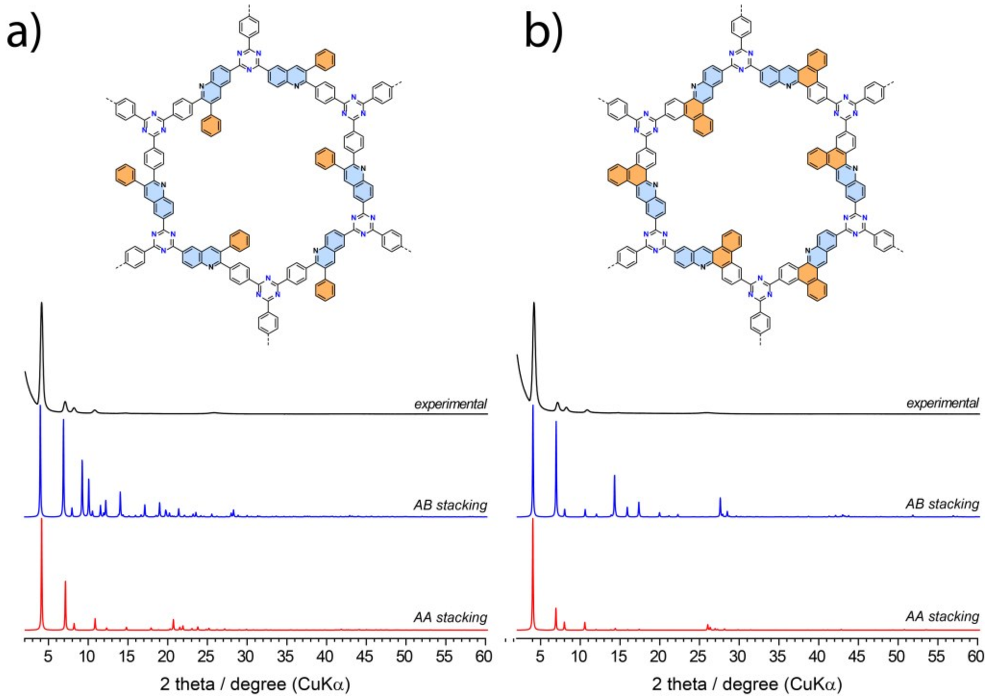

Figure S8. Simulated X-ray diffraction patterns for the generated **hcb** hexagonal layered structures adopting fully eclipsed (red) and staggered (blue) stacking arrangement compared to the experimentally obtained patterns (black) **P3Qy-2** (a) and **P3Qy-2RC** (b).

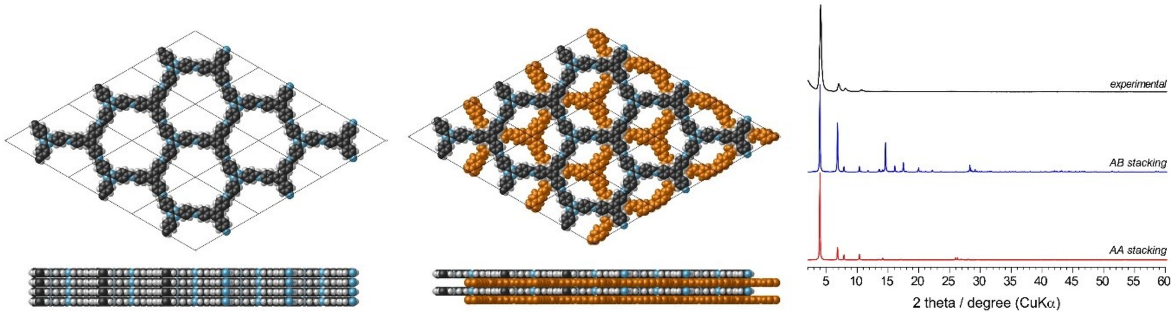

Figure S9. Simulated **hcb** 2D hexagonal layered model with eclipsed (AA), staggered (AB) stacking arrangement of **Im-1.** Simulated X-ray diffraction patterns for generated hcb hexagonal layered structures adopting fully eclipsed (red) and staggered (blue) stacking arrangement compared to the experimentally obtained pattern of **Im-1** (black).

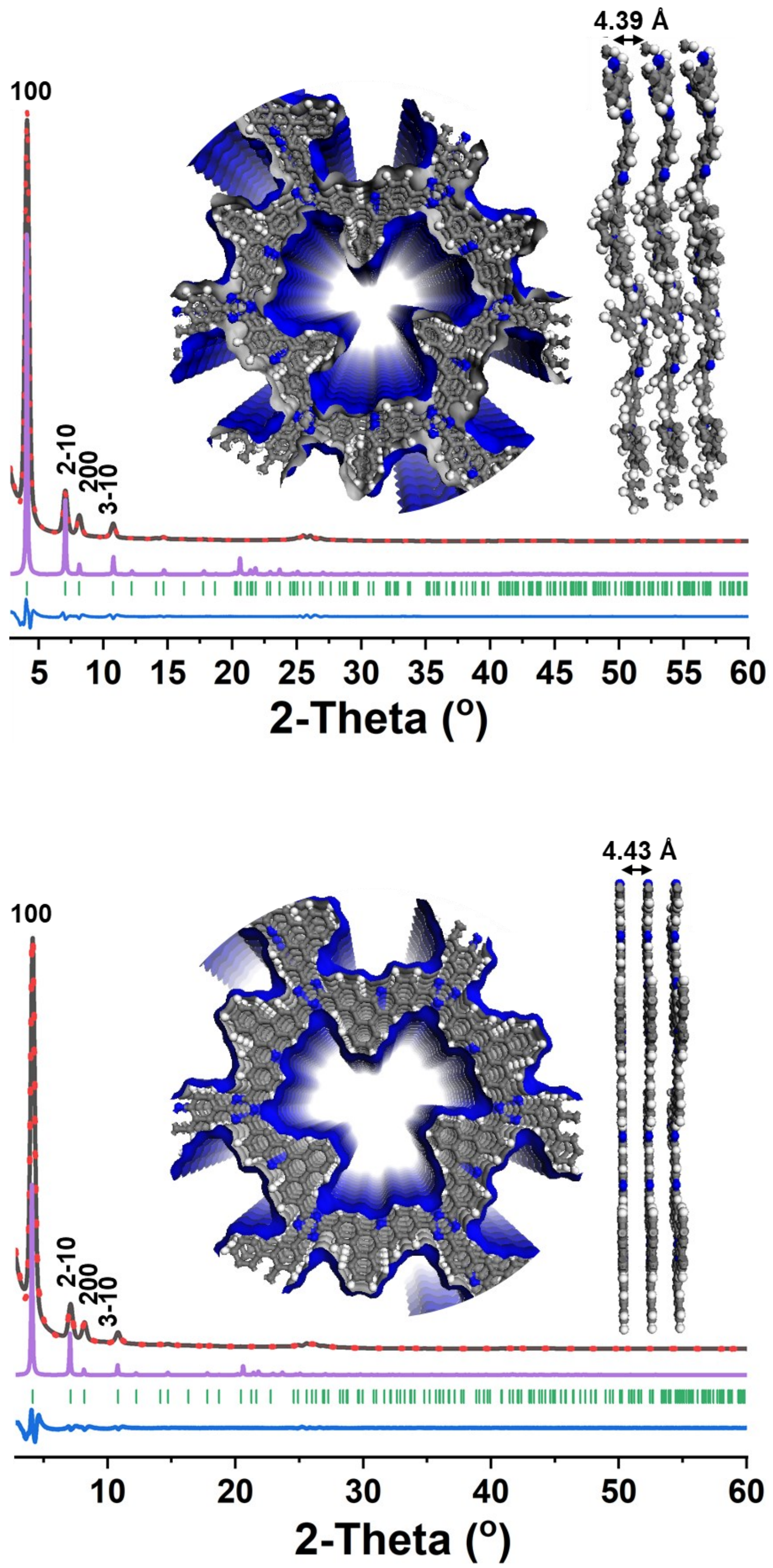

Figure S10. Pawley refined (red dotted line), experimental (black line) PXRD pattern with minimum difference (green line) for hexagonal AA stacking (blue) of **P3Qy-2 (top)** and **P3Qy-2RC (bottom)**. (Inset) Top and side view of AA stacking of **P3Qy-2**. (Color code: C, gray spheres; N, blue spheres, H, white spheres).

Table S4. Fractional Co-ordinates for COFs

P3Qy-1_Pawley				
Space Group: P*3* (143)<br>*a* = 25.2940 Å, *b* = 25.2940 Å, *c* = 4.3407 Å<br>*α* = 90.0000 °, *β* = 90.0000 °, *γ* = 120.0000 °				
**Atom label**	**Atom type**	***x***	***y***	***z***
H1	H	0.35470	0.52847	1.18696
H2	H	0.82883	0.76272	0.06269
C3	C	0.39508	0.55290	1.04091
C4	C	0.42917	0.52387	0.96790
C5	C	0.48236	0.55443	0.78555
C6	C	0.50264	0.61495	0.68342
C7	C	0.46719	0.64312	0.74569
C8	C	0.41253	0.61223	0.92318
C9	C	0.55832	0.64616	0.52077
C10	C	0.59388	0.61774	0.45847
C11	C	0.56759	0.55349	0.53576
N12	N	0.51389	0.52540	0.70183
C13	C	0.65793	0.65824	0.33636
C14	C	0.68297	0.63811	0.10553
C15	C	0.74482	0.67486	0.01324
C16	C	0.78128	0.73399	0.13742
C17	C	0.75614	0.75618	0.35608
C18	C	0.69506	0.71861	0.45612
H19	H	0.41413	0.47761	1.05349
H20	H	0.48149	0.68820	0.64652
H21	H	0.57478	0.69351	0.44734
H22	H	0.76438	0.65815	-0.15867
H23	H	0.78434	0.80204	0.45301
H24	H	0.67767	0.73607	0.63579
C25	C	0.59250	0.51258	0.44122
C26	C	0.65414	0.52745	0.49419
C27	C	0.67332	0.48458	0.43200
C28	C	0.63102	0.42518	0.32087
C29	C	0.56977	0.41055	0.26066
C30	C	0.55084	0.45375	0.32012
H31	H	0.72089	0.49735	0.48149
H32	H	0.53610	0.36528	0.17291
H33	H	0.50298	0.44074	0.27659
H34	H	0.65464	0.59335	0.00306
H35	H	0.68799	0.57198	0.58303
C36	C	0.37213	0.64021	0.96673
C37	C	0.30722	0.60226	0.97127
C38	C	0.64960	0.37753	-0.71037

N39	N	0.71055	0.39416	-0.70934
H40	H	0.44754	0.73455	0.96498

**P3Qy-1RC_Pawley**				
Space Group: P$\bar{6}$ (174)<br>a = 25.3131 Å, b = 25.3131 Å, c = 3.4351 Å<br>α = 90.0000 °, β = 90.0000 °, γ = 120.0000 °				
**Atom label**	**Atom type**	***x***	***y***	***z***
H1	H	0.31581	0.50808	0.00000
H2	H	0.81660	0.68563	0.00000
C3	C	0.36337	0.53420	0.00000
C4	C	0.39251	0.50145	0.00000
C5	C	0.45408	0.53006	0.00000
C6	C	0.48715	0.59196	0.00000
C7	C	0.45808	0.62514	0.00000
C8	C	0.39513	0.59725	0.00000
C9	C	0.54917	0.61971	0.00000
C10	C	0.57805	0.58592	0.00000
C11	C	0.54227	0.52361	0.00000
N12	N	0.48209	0.49804	0.00000
C13	C	0.64430	0.61358	0.00000
C14	C	0.67040	0.57678	0.00000
C15	C	0.73308	0.60417	0.00000
C16	C	0.76879	0.66561	0.00000
C17	C	0.74334	0.70134	0.00000
C18	C	0.68194	0.67593	0.00000
H19	H	0.36681	0.45344	0.00000
H20	H	0.48625	0.67251	0.00000
H21	H	0.57361	0.66745	0.00000
H22	H	0.75609	0.57950	0.00000
H23	H	0.77136	0.74911	0.00000
H24	H	0.66536	0.70638	0.00000
C25	C	0.56917	0.48525	0.00000
C26	C	0.63144	0.51040	0.00000
C27	C	0.65489	0.47167	0.00000
C28	C	0.61779	0.40973	0.00000
C29	C	0.55628	0.38602	0.00000
C30	C	0.53231	0.42322	0.00000
H31	H	0.70191	0.48849	0.00000
H32	H	0.52638	0.33851	0.00000
H33	H	0.48449	0.40303	0.00000
C36	C	0.36326	0.63311	0.00000
C37	C	0.30077	0.60506	0.00000
H40	H	0.44185	0.71789	0.00000

C38	C	0.64328	0.36994	0.00000
N39	N	0.70298	0.39284	0.00000

**P4Qy-1_Pawley**				
Space Group: P*3* (143)<br>*a* = 25.4598 Å, *b* = 25.4598 Å, *c* = 3.6646 Å<br>*α* = 90.0000 °, *β* = 90.0000 °, *γ* = 120.0000 °				
**Atom label**	**Atom type**	***x***	***y***	***z***
H	H	0.44416	0.71850	1.16835
C1	C	0.59224	0.69109	1.15701
C2	C	0.57717	0.73318	1.01864
C3	C	0.61490	0.79479	1.07518
C4	C	0.66985	0.81576	1.25266
C5	C	0.68660	0.77506	1.38025
C6	C	0.64802	0.71327	1.33613
H7	H	0.53705	0.71897	0.85791
H8	H	0.60232	0.82639	0.97075
H9	H	0.69949	0.86354	1.29084
H10	H	0.72918	0.79133	1.51880
H11	H	0.66183	0.68294	1.44537
C12	C	0.58114	0.59153	0.99371
C13	C	0.55310	0.62489	1.08806
H14	H	0.62948	0.61418	0.98161
C15	C	0.26950	0.63699	-0.83323
N16	C	0.29988	0.60405	-0.82778
C17	C	-0.20741	-0.62164	-1.27752
C18	C	-0.18534	-0.65861	-1.15323
C19	C	-0.12377	-0.63535	-1.10034
C20	C	-0.08229	-0.57454	-1.17793
C21	C	-0.10478	-0.53824	-1.31306
C22	C	-0.16645	-0.56136	-1.36015
N23	N	0.01884	-0.48630	-1.12621
C24	C	-0.01653	-0.54695	-1.10132
C25	C	0.10584	-0.48919	-0.91637
C26	C	0.07830	-0.45709	-1.04632
C27	C	0.11221	-0.39516	-1.10023
C28	C	0.17391	-0.36460	-1.03490
C29	C	0.20229	-0.39446	-0.88135
C30	C	0.16609	-0.45619	-0.79997
H31	H	-0.21589	-0.70514	-1.08272
H32	H	-0.10994	-0.66526	-0.98659
H33	H	-0.07478	-0.49140	-1.37865
H34	H	-0.18206	-0.53183	-1.45973

H35	H	0.09129	-0.37099	-1.21140
H36	H	0.19867	-0.31800	-1.11612
H37	H	0.18469	-0.47866	-0.64580
C38	C	0.64510	0.37220	-0.30262
N39	N	0.60663	0.31191	-0.30178

**Im-1_Pawley**				
Space Group: P$\bar{6}$ (174)<br>*a* = 25.3338 Å, *b* = 25.3338 Å, *c* = 3.4426 Å<br>*α* = 90.0000 °, *β* = 90.0000 °, *γ* = 120.0000 °				
**Atom label**	**Atom type**	***x***	***y***	***z***
H1	H	0.54064	0.62827	0.00000
H2	H	0.27848	0.71954	0.00000
H3	H	0.58031	0.57380	0.00000
C4	C	0.27037	0.63461	0.00000
C5	C	0.30332	0.60552	0.00000
C6	C	-0.20934	-0.61947	0.00000
C7	C	-0.18543	-0.65692	0.00000
C8	C	-0.12454	-0.63371	0.00000
C9	C	-0.08630	-0.57269	0.00000
C10	C	-0.11001	-0.53511	0.00000
C11	C	-0.17090	-0.55828	0.00000
N12	N	0.01422	-0.49289	0.00000
C13	C	-0.02215	-0.54929	0.00000
C14	C	0.10932	-0.49323	0.00000
C15	C	0.07745	-0.46342	0.00000
C16	C	0.10829	-0.40217	0.00000
C17	C	0.16971	-0.37069	0.00000
C18	C	0.20321	-0.39958	0.00000
C19	C	0.17079	-0.46202	0.00000
H20	H	-0.21369	-0.70425	0.00000
H21	H	-0.10727	-0.66353	0.00000
H22	H	-0.08160	-0.48780	0.00000
H23	H	-0.18776	-0.52813	0.00000
H24	H	0.08440	-0.37841	0.00000
H25	H	0.18904	-0.32368	0.00000
H26	H	0.19110	-0.48882	0.00000
C27	C	0.64405	0.37013	0.00000
N28	N	0.60771	0.31089	0.00000

P3Qy-2_Pawley				
Space Group: P*3* (143)<br>*a* = 25.1959 Å, *b* = 25.1959 Å, *c* = 4.3958 Å<br>*α* = 90.0000 °, *β* = 90.0000 °, *γ* = 120.0000 °				
**Atom label**	**Atom type**	***x***	***y***	***z***
H1	H	0.33222	0.51756	0.95908
H2	H	0.83225	0.75328	0.15175
C3	C	0.37946	0.54494	0.88257
C4	C	0.41331	0.51550	0.82499
C5	C	0.47379	0.54931	0.71778
C6	C	0.50137	0.61318	0.67769
C7	C	0.46767	0.64289	0.73846
C8	C	0.40596	0.60878	0.83542
C9	C	0.56182	0.64582	0.57020
C10	C	0.59506	0.61565	0.50104
C11	C	0.56413	0.54996	0.53494
N12	N	0.50531	0.52023	0.64413
C13	C	0.66009	0.65466	0.39559
C14	C	0.68390	0.63534	0.15938
C15	C	0.74615	0.66960	0.07872
C16	C	0.78435	0.72623	0.21800
C17	C	0.76044	0.74822	0.44010
C18	C	0.69885	0.71268	0.52961
H19	H	0.39197	0.46617	0.85766
H20	H	0.48885	0.69207	0.69985
H21	H	0.58269	0.69506	0.53447
H22	H	0.76445	0.65310	-0.09743
H23	H	0.78995	0.79220	0.54802
H24	H	0.68232	0.72969	0.71196
C25	C	0.58999	0.50949	0.45376
C26	C	0.64997	0.52328	0.53306
C27	C	0.66994	0.48121	0.47747
C28	C	0.63005	0.42363	0.34684
C29	C	0.57057	0.40995	0.26160
C30	C	0.55093	0.45244	0.31357
H31	H	0.71607	0.49305	0.54657
H32	H	0.53893	0.36607	0.15801
H33	H	0.50452	0.44025	0.24782
H34	H	0.65401	0.59306	0.04271
H35	H	0.68210	0.56644	0.63631
C36	C	0.36828	0.63892	0.86573
N37	N	0.30579	0.60444	0.86478
C38	C	0.64914	0.37679	-0.68162
N39	N	0.70985	0.39393	-0.68061

P3Qy-2RC_Pawley				
Space Group: P$\bar{6}$ (174)<br>*a* = 25.1808 Å, *b* = 25.1808 Å, *c* = 3.4361 Å<br>*α* = 90.0000 °, *β* = 90.0000 °, *γ* = 120.0000 °				
**Atom label**	**Atom type**	***x***	***y***	***z***
H1	H	0.81798	0.68913	0.00000
H2	H	0.31147	0.51219	0.00000
C3	C	0.35996	0.53592	0.00000
C4	C	0.38921	0.50263	0.00000
C5	C	0.45171	0.53176	0.00000
C6	C	0.48536	0.59454	0.00000
C7	C	0.45601	0.62799	0.00000
C8	C	0.39299	0.59896	0.00000
C9	C	0.54791	0.62256	0.00000
C10	C	0.57704	0.58838	0.00000
C11	C	0.54089	0.52541	0.00000
N12	N	0.48007	0.49950	0.00000
C13	C	0.64395	0.61632	0.00000
C14	C	0.67033	0.57917	0.00000
C15	C	0.73363	0.60684	0.00000
C16	C	0.76970	0.66890	0.00000
C17	C	0.74397	0.70497	0.00000
C18	C	0.68196	0.67929	0.00000
H19	H	0.36315	0.45416	0.00000
H20	H	0.48228	0.67648	0.00000
H21	H	0.57251	0.67079	0.00000
H22	H	0.75689	0.58194	0.00000
H23	H	0.77226	0.75323	0.00000
H24	H	0.66519	0.71004	0.00000
C25	C	0.56809	0.48669	0.00000
C26	C	0.63100	0.51212	0.00000
C27	C	0.65470	0.47301	0.00000
C28	C	0.61725	0.41045	0.00000
C29	C	0.55512	0.38648	0.00000
C30	C	0.53089	0.42404	0.00000
H31	H	0.70219	0.49002	0.00000
H32	H	0.52493	0.33850	0.00000
H33	H	0.48259	0.40362	0.00000
C34	C	0.36193	0.63422	0.00000
N35	N	0.30114	0.60610	0.00000
C36	C	0.64302	0.37029	0.00000
N37	N	0.70332	0.39344	0.00000

Section S4. Structure Characterization of MCR-COFs

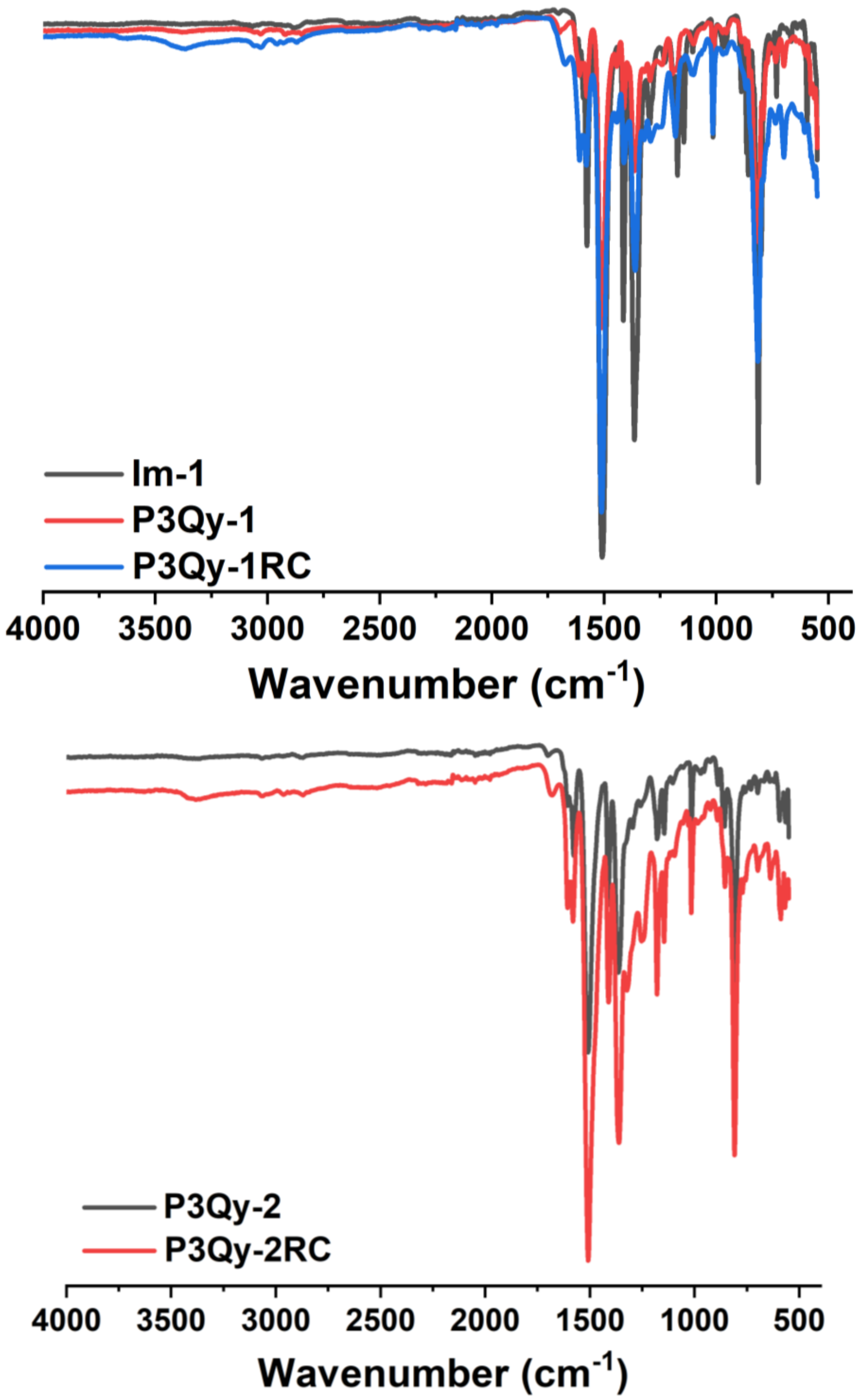

Figure S11. FTIR spectra of **Im-1**, **P3Qy-1, P3Qy-1RC**, **P3Qy-2** and **P3Qy-2RC**. The appearance of characteristic bands at 1579 cm^{-1} associated with pyridyl stretching band demonstrated the successful linkage formation.

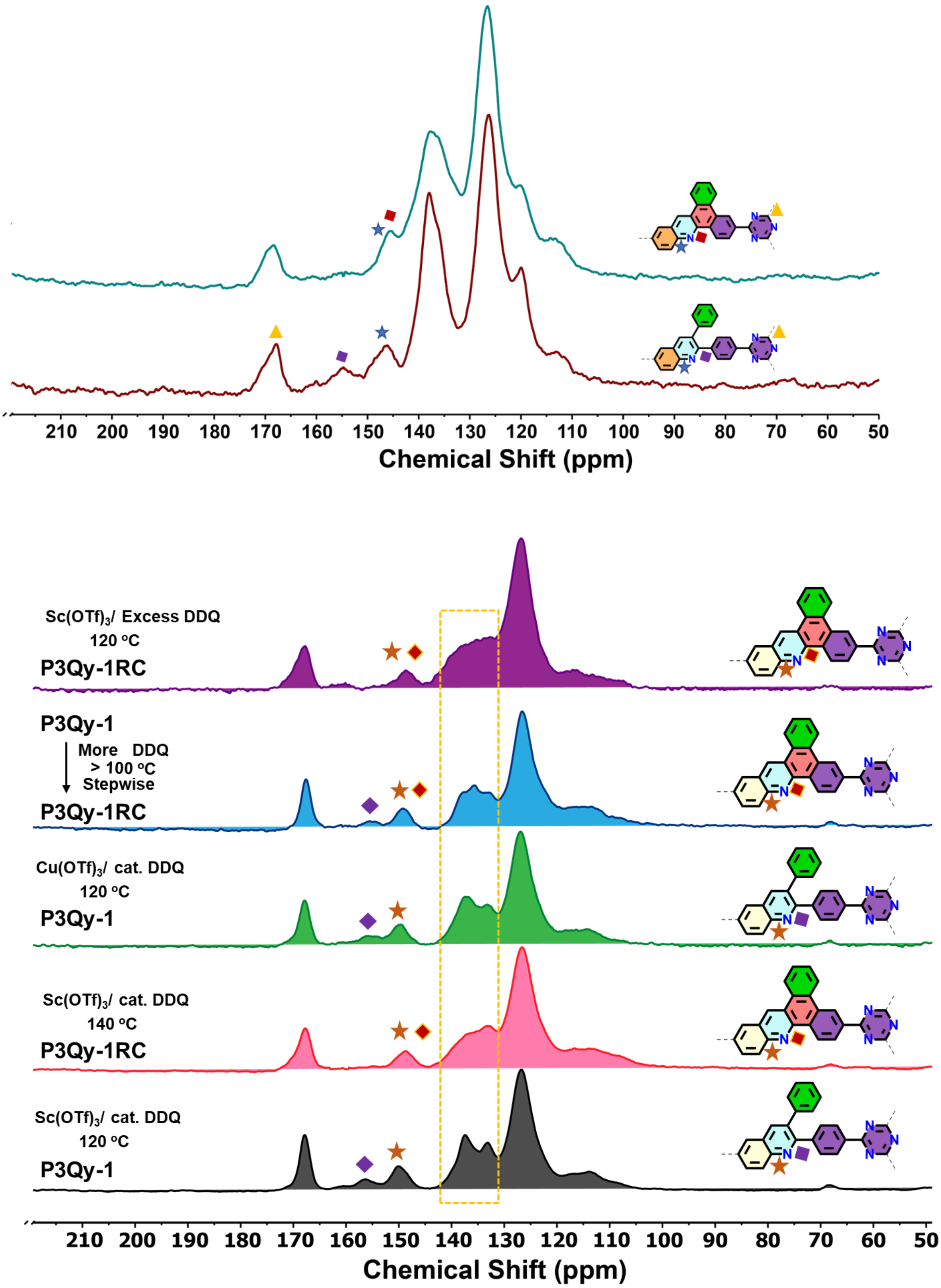

Figure S12. (Top) ^{13}C (CP/MAS) NMR spectra measured at 10 kHz for **P3Qy-2** and **P3Qy-2RC**. (Bottom) ^{13}C (CP/MAS) NMR spectra measured at 10 kHz for **P3Qy-1** and **P3Qy-1RC** in different reaction condition. The brown dotted box reflecting the broadening of NMR spectra in **P3Qy-1RC** compared to **P3Qy-1** due to restrict of rotation.

(a)

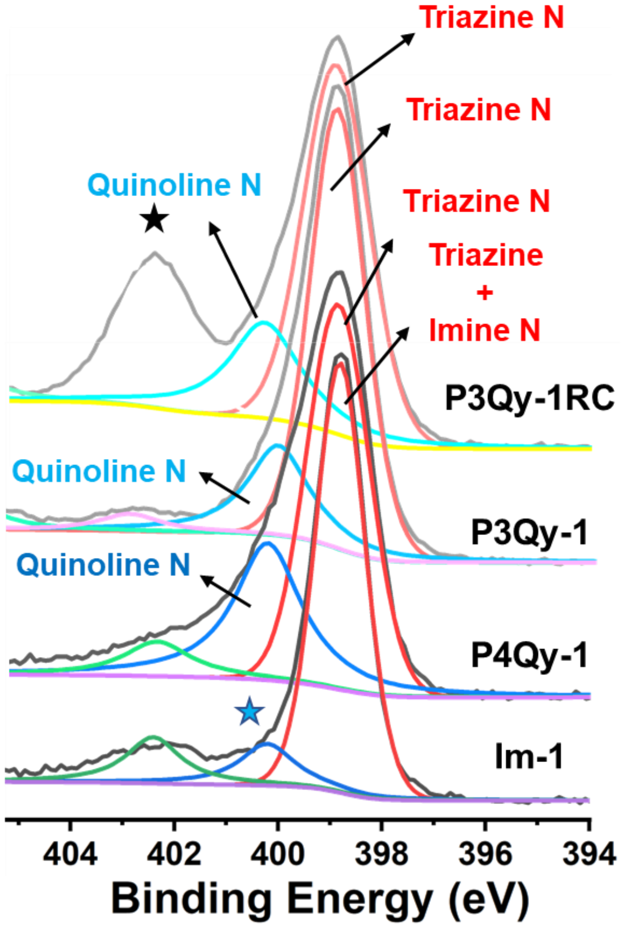

(b)

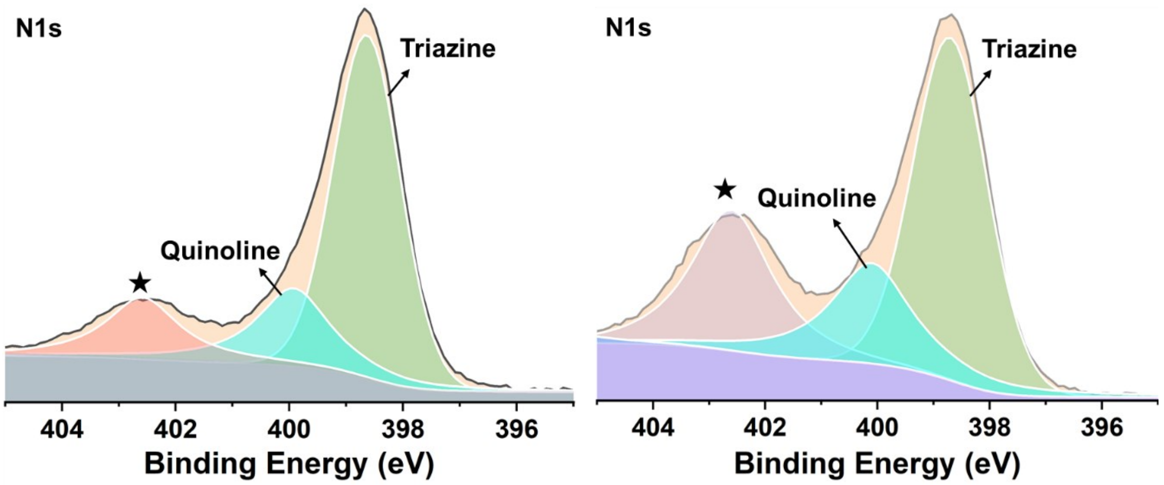

Figure S13. (a) N(1s) XPS spectra of **Im-1**, **P4Qy-1**, **P3Qy-1**, and **P3Qy-1RC** (b) N(1s) XPS spectra of **P3Qy-2 (left)** and **P3Qy-2RC (right)** (N.B. the blue star in **Im-1** corresponds to surface free amines, and the black star at 402.5 eV corresponds to surface oxidation of N which increased at higher temperature (**P3Qy-1RC**)).

Section S5. FESEM and HRTEM Images of COFs

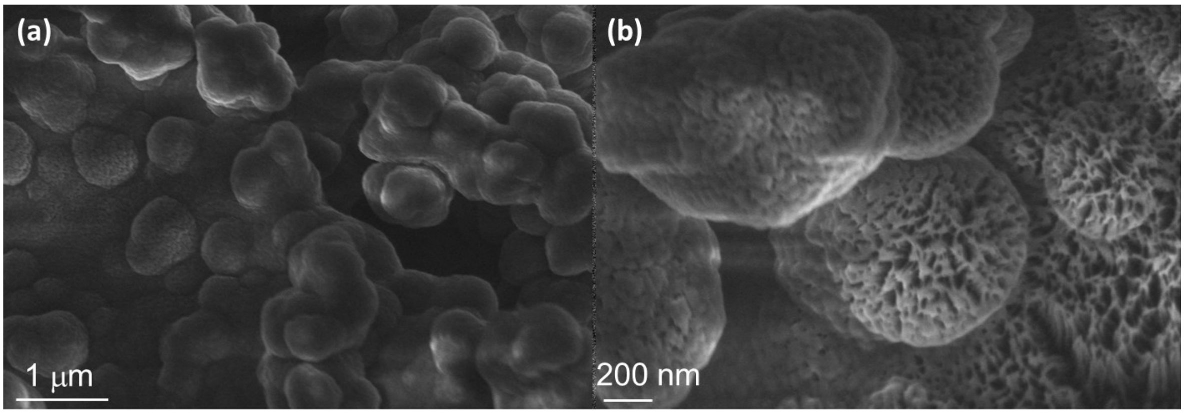

Figure S14. FESEM image of **Im-1**.

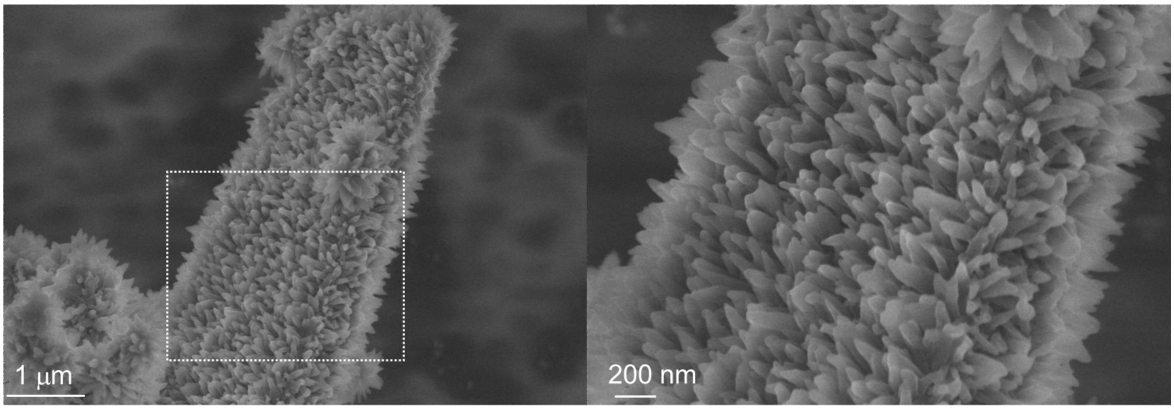

Figure S15. FESEM image of **P3Qy-1**.

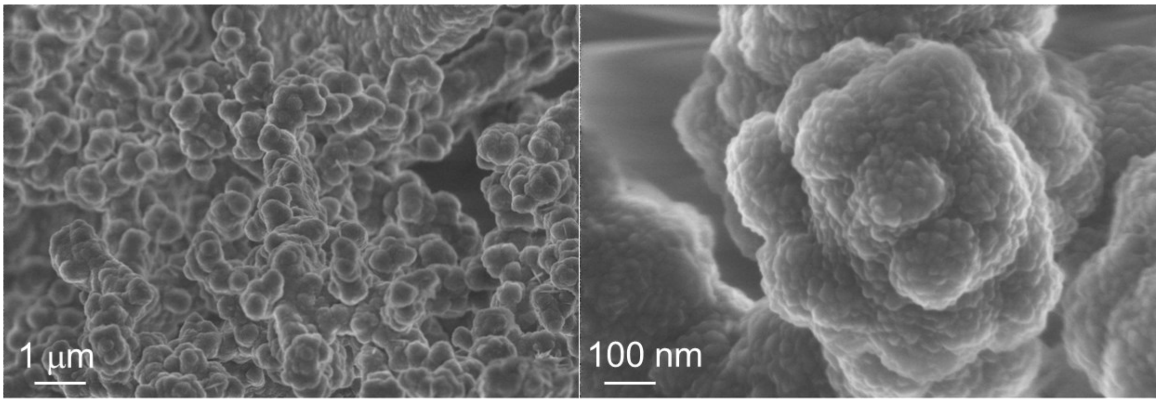

Figure S16. FESEM image of **P3Qy-1RC**.

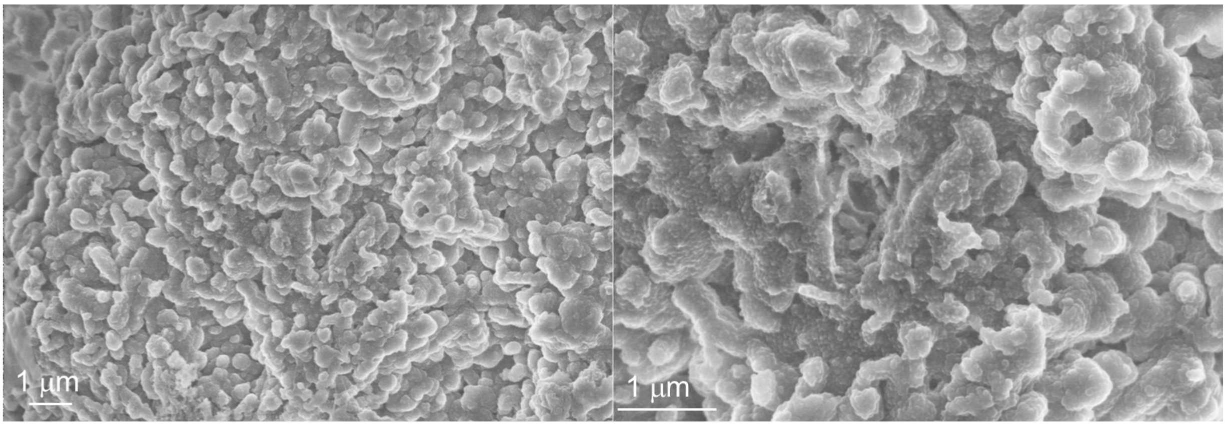

Figure S17. FESEM image of **P4Qy-1**.

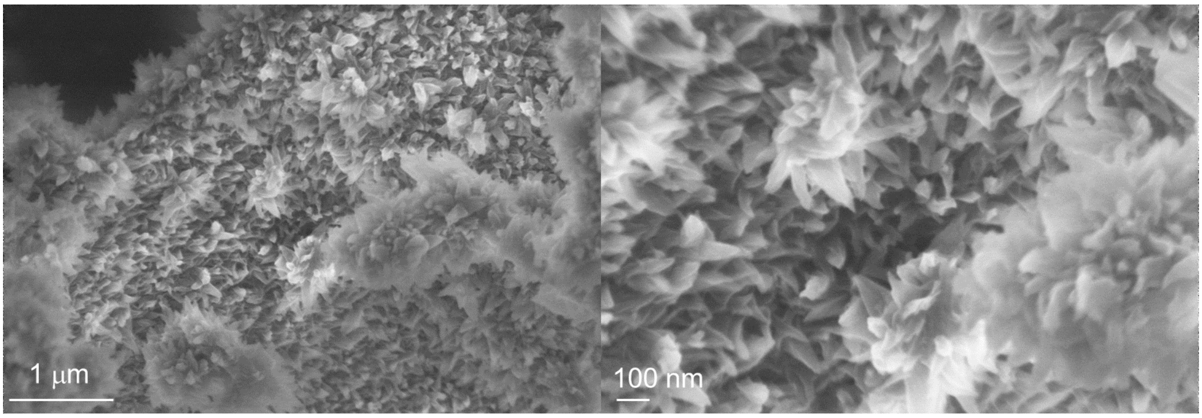

Figure S18. FESEM image of **P3Qy-2**.

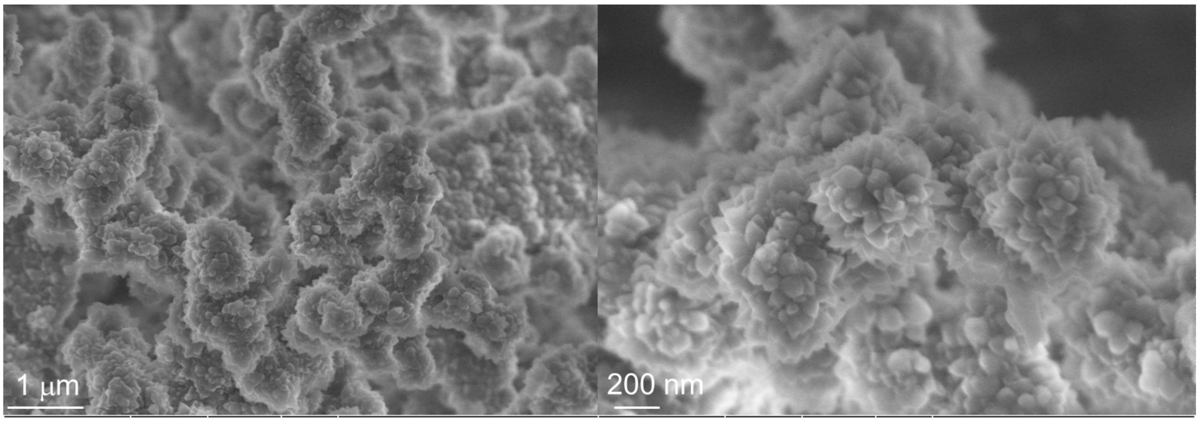

Figure S19. FESEM image of **P3Qy-2RC**.

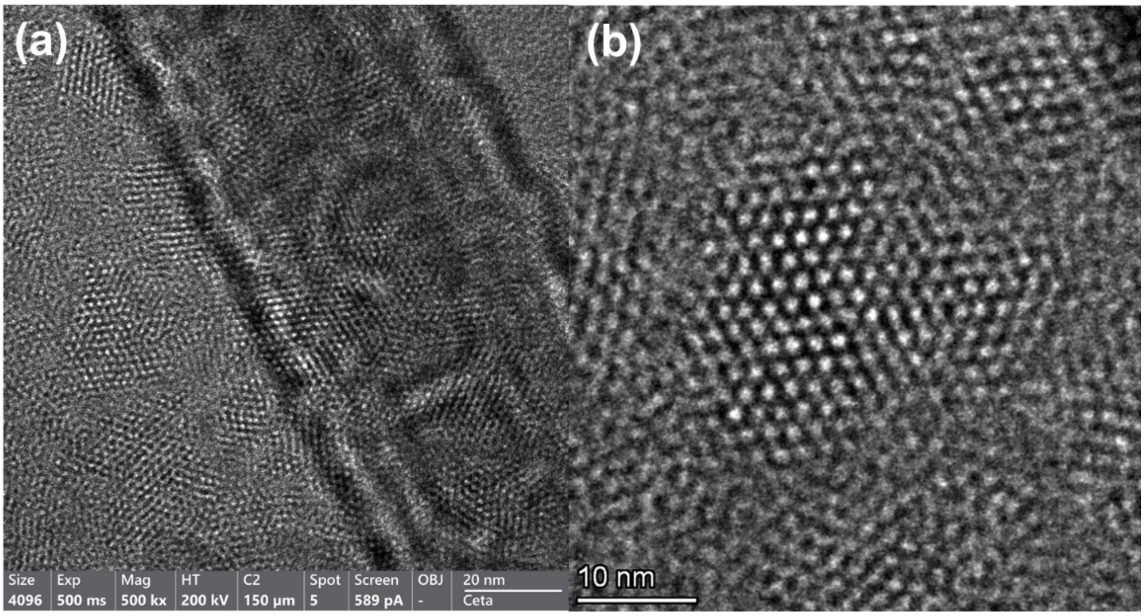

Figure S20. HRTEM image of **Im-1** and its magnified view.

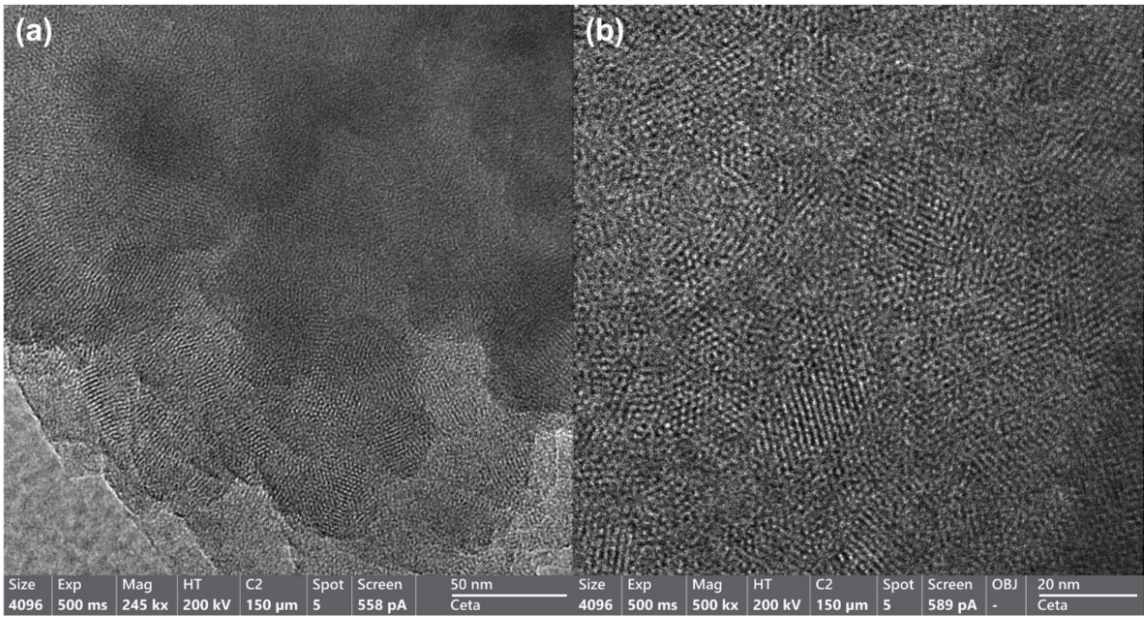

Figure S21. HRTEM image of **P4Qy-1** and its magnified view.

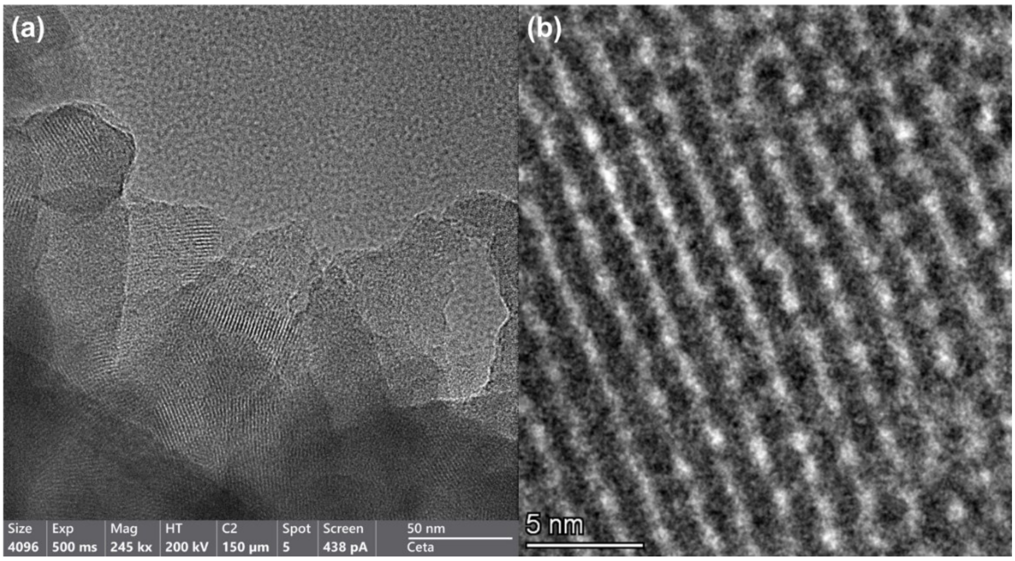

Figure S22. HRTEM image of **P3Qy-2** and its magnified view.

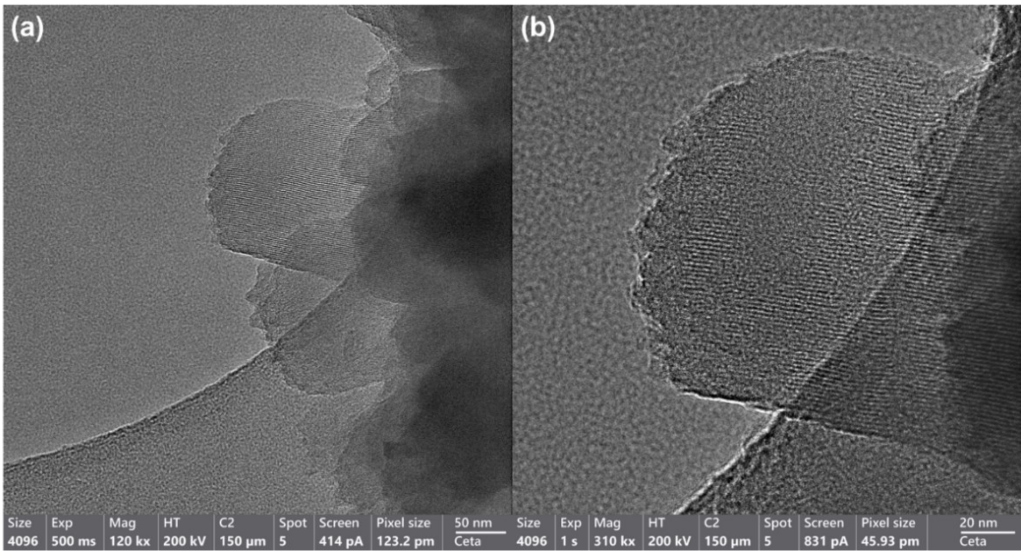

Figure S23. HRTEM image of **P3Qy-2RC** and its magnified view.

Section S6. Chemical and Thermal Stability of MCR-COFs

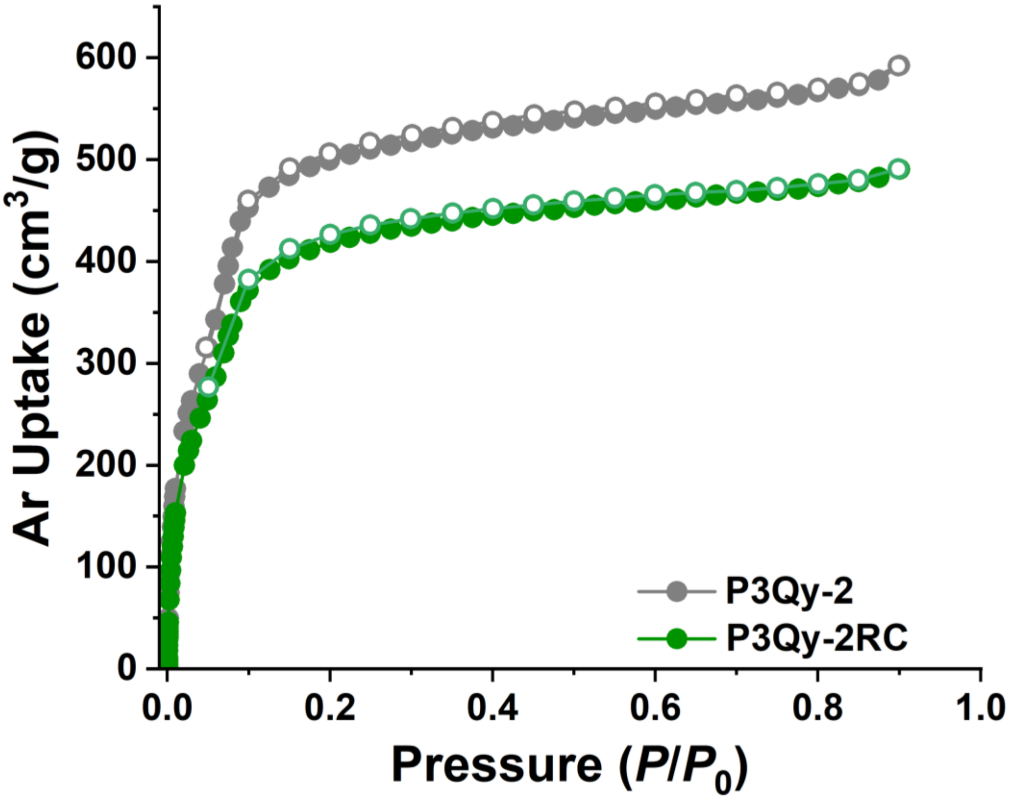

Figure S24. Ar sorption isotherm of **P3Qy-2**, **P3Qy-2RC** at 87 K.

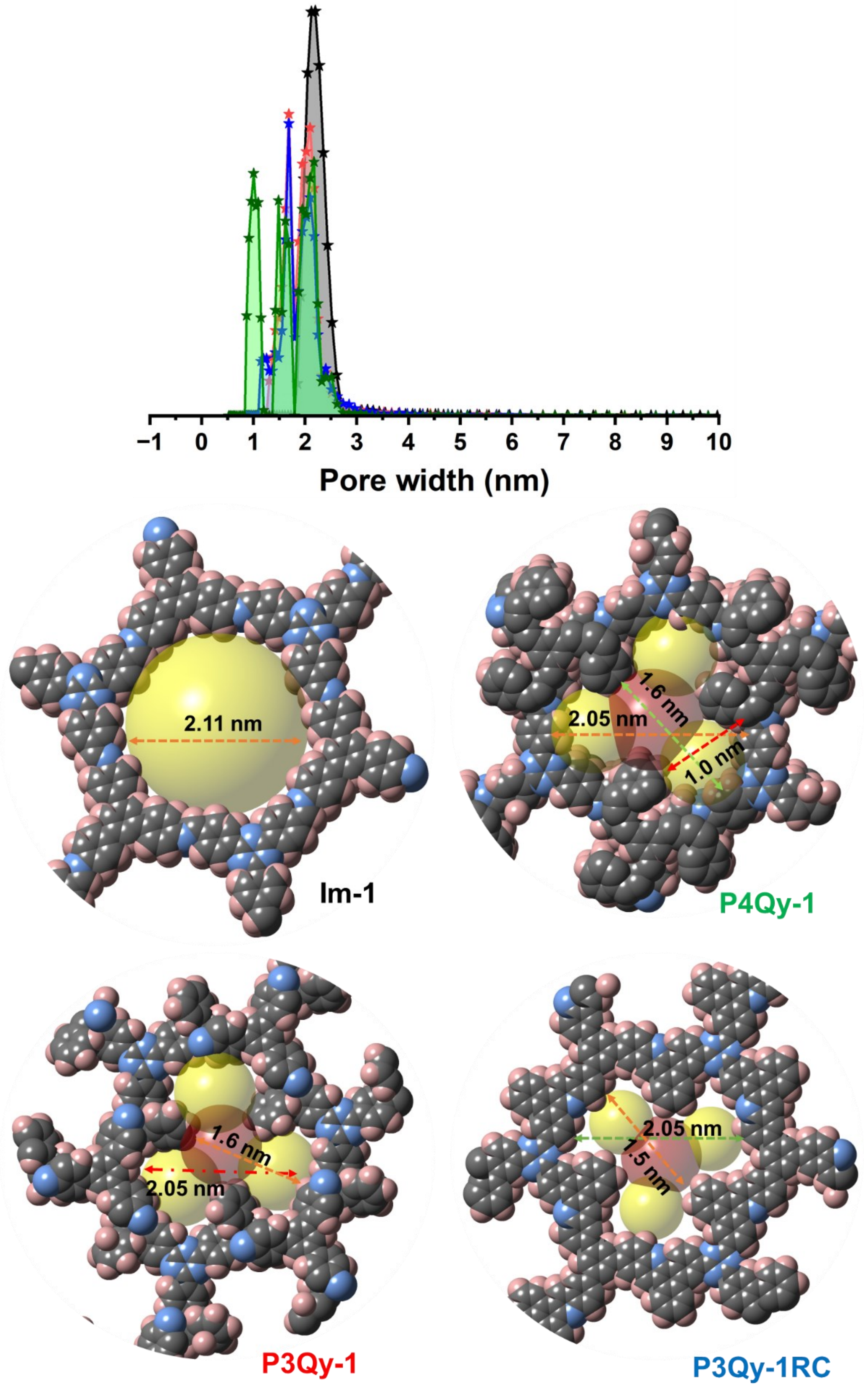

Figure S25. Ar sorption and pore size distribution of MCR-COFs and compared with **Im-1** using NLDFT method (Distance follow van der Wall radii).

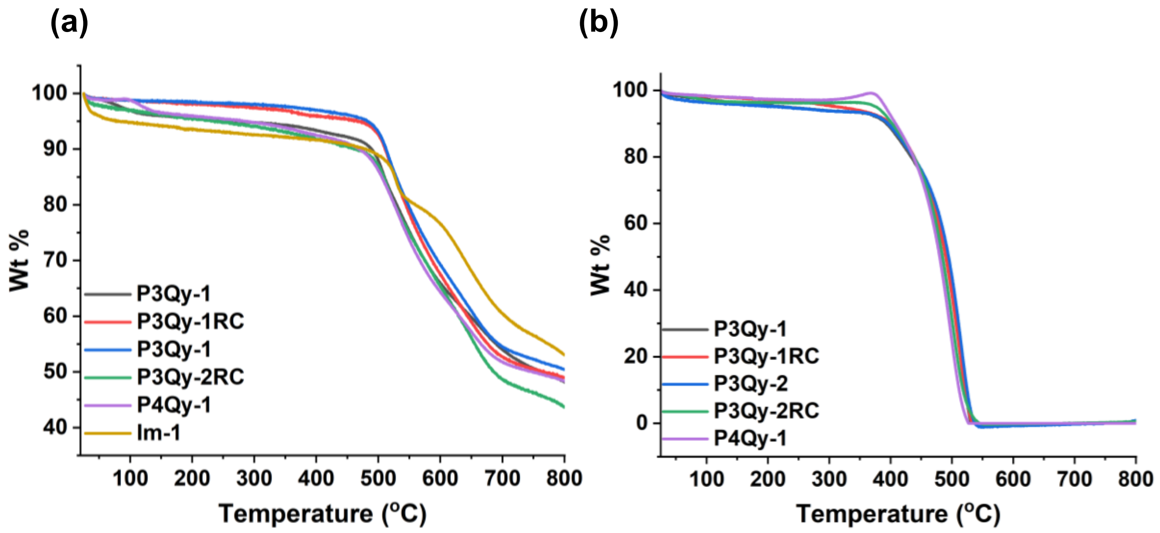

Figure S26. TGA profiles of COFs under (a) N_2 and (b) air atmosphere showing high thermal stability up to 500 and 400 °C, respectively.

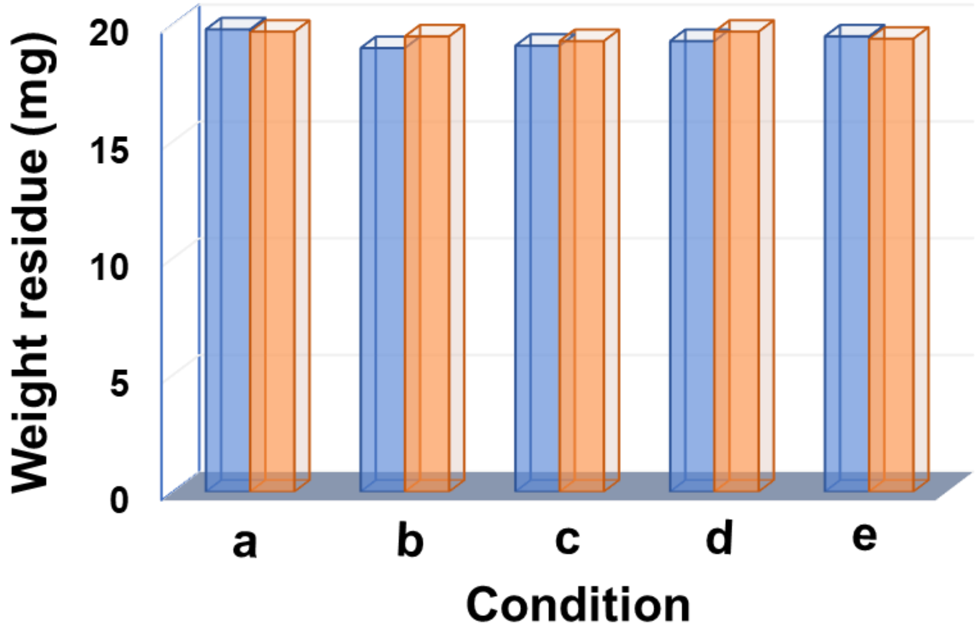

Figure S27. Residue weight of **P3Qy-2 (blue)** and **P3Qy-2RC (brown)** after 7-day treatment with (a) 9 M HCl (aq.), (b) 6 M NaOH (aq.), and (c) 3 M $Na_2S_2O_5$ (aq.), (d) 0.5 M H_2O_2 and (e) boiling water 1 day, respectively.

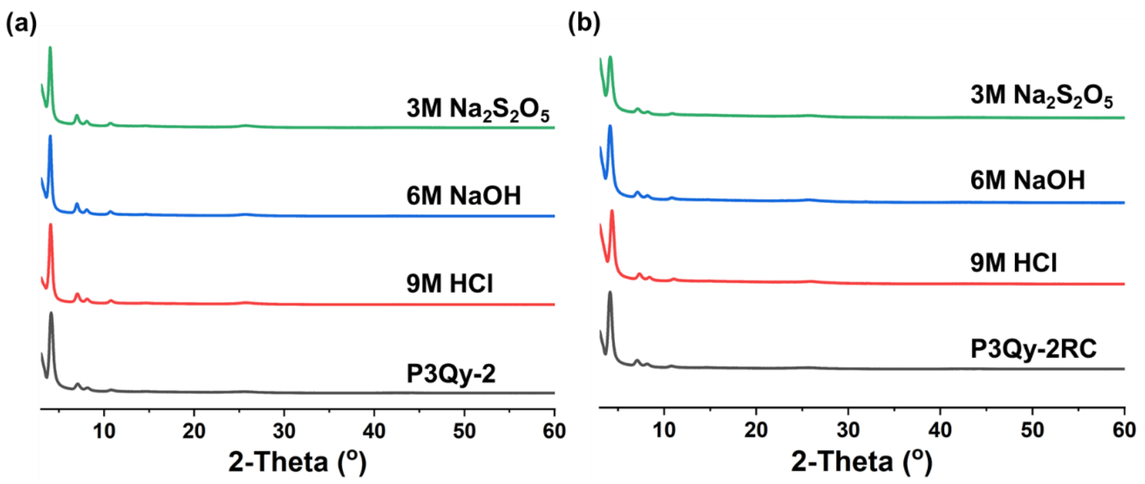

Figure S28. Stability of (a) **P3Qy-2** and (b) **P3Qy-2RC** after 7-day treatment with 9 M HCl (aq.), 6 M NaOH (aq.), and 3 M $Na_2S_2O_5$ (aq.) monitored by PXRD.

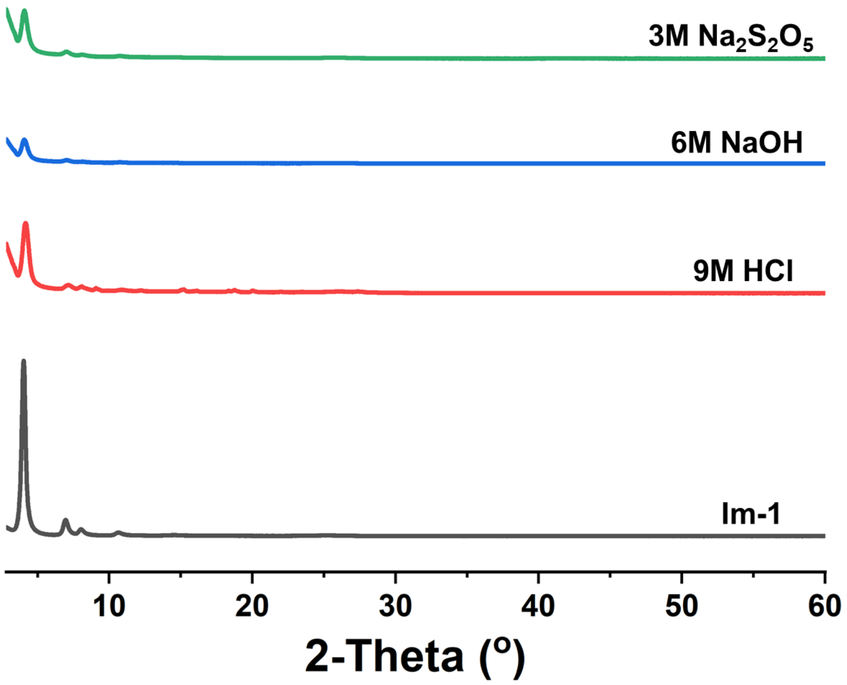

Figure S29. Stability of **Im-1** after 7-day treatment with 9 M HCl (aq.), 6 M NaOH (aq.), and 3 M $Na_2S_2O_5$ (aq.) monitored by PXRD.

Section S7. Stepwise Synthesis of Novel MCR-COF

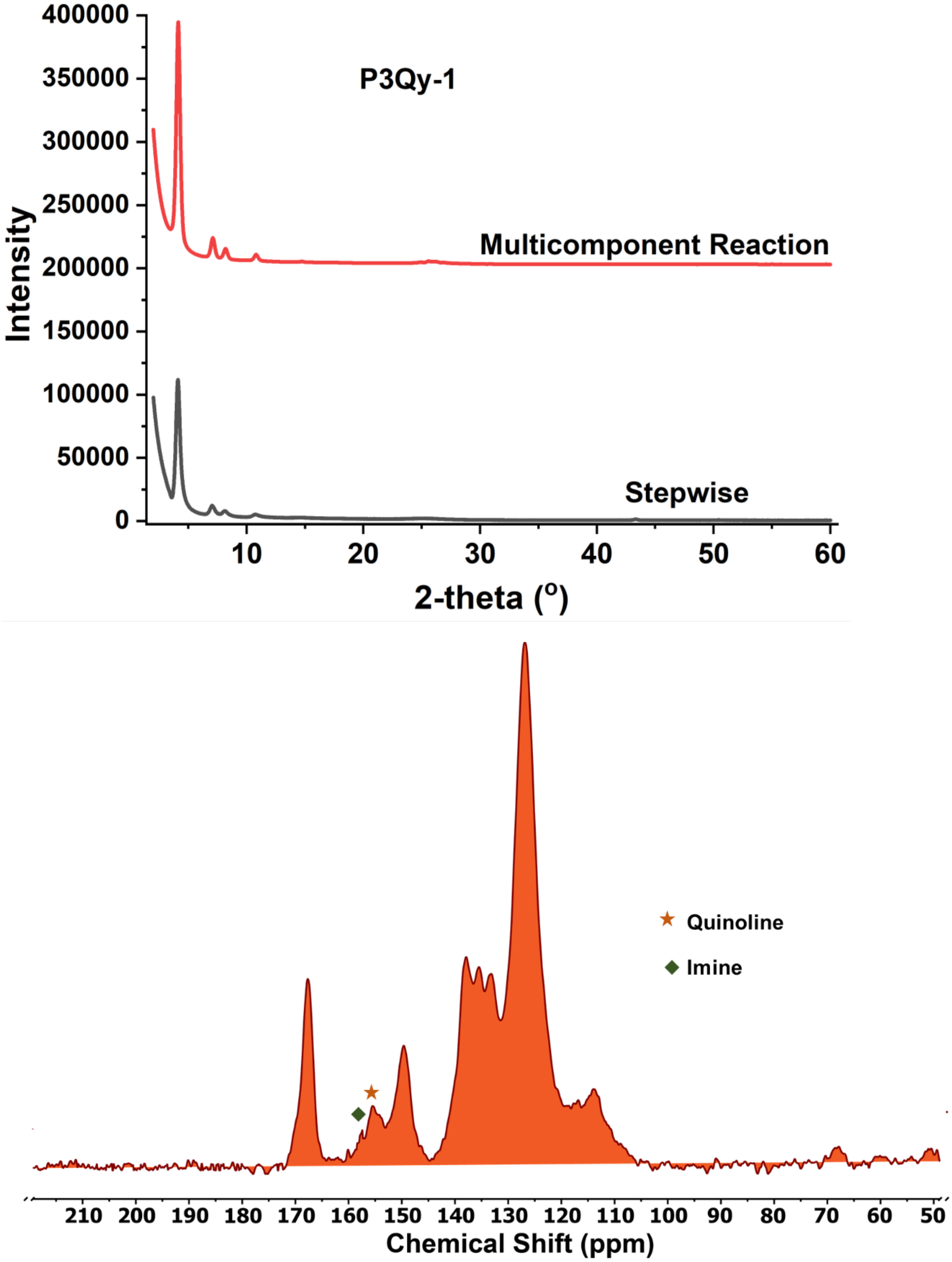

Figure S30. PXRD pattern and ^{13}C (CP/MAS) NMR spectrum of **P3Qy-1** via stepwise synthesis compared to multicomponent synthesis (peak corresponding to imine was left after PSM near 158 ppm).

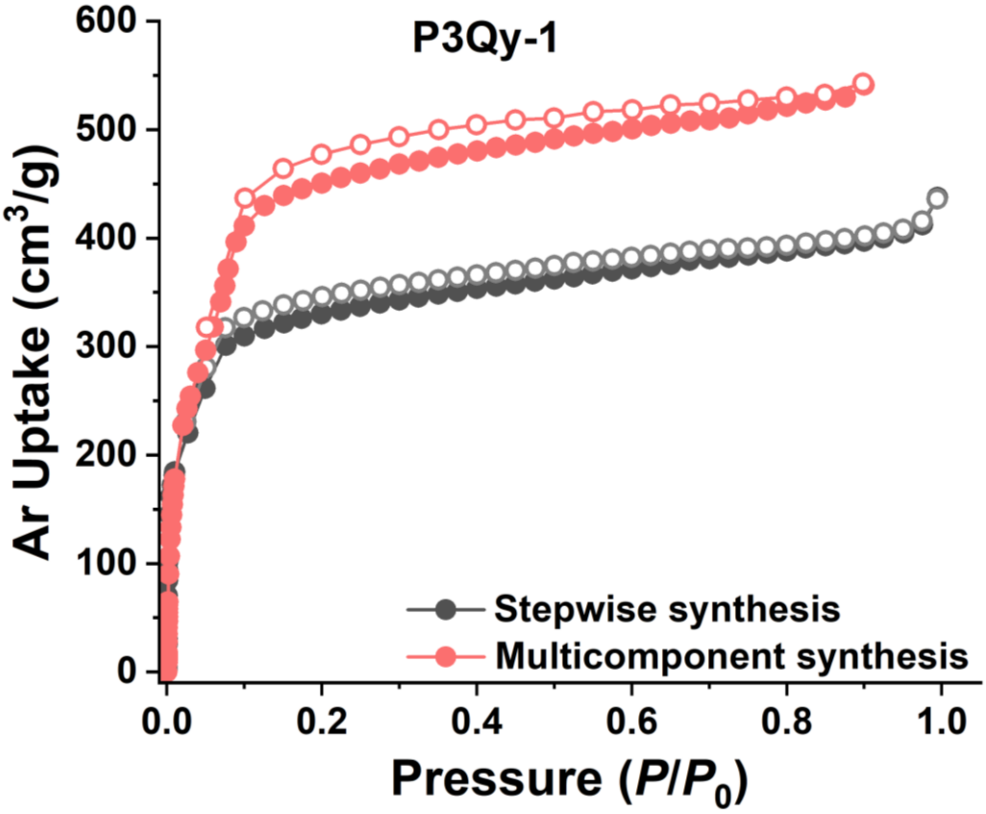

Figure S31. Ar sorption of **P3Qy-1** via stepwise synthesis compared to multicomponent synthesis.

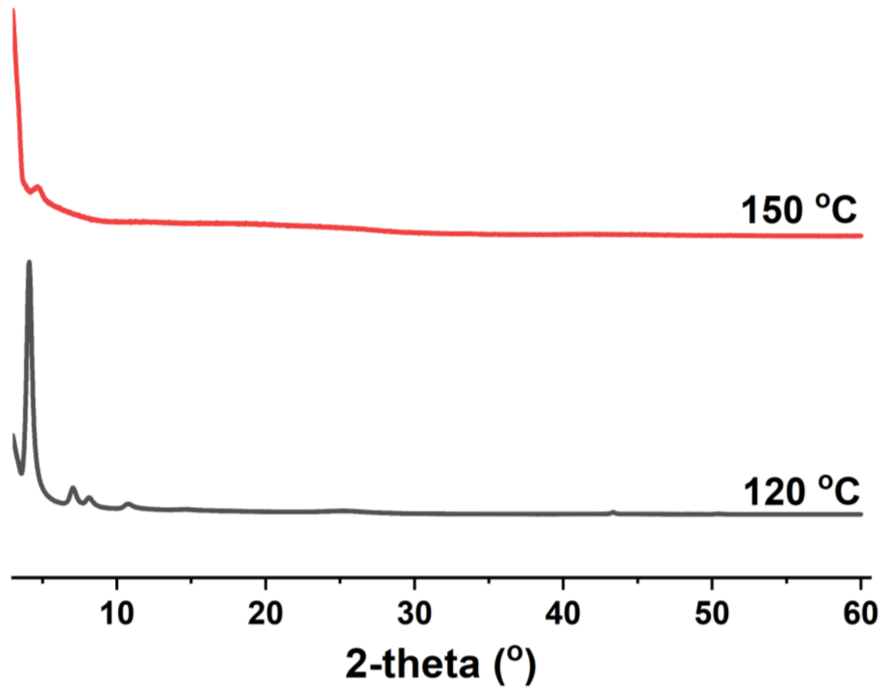

Figure S32. PXRD patterns of **Im-1** after PSM at 120 °C and 150 °C.

Section S8. UV-vis Spectra and Band Structure of MCR-COFs

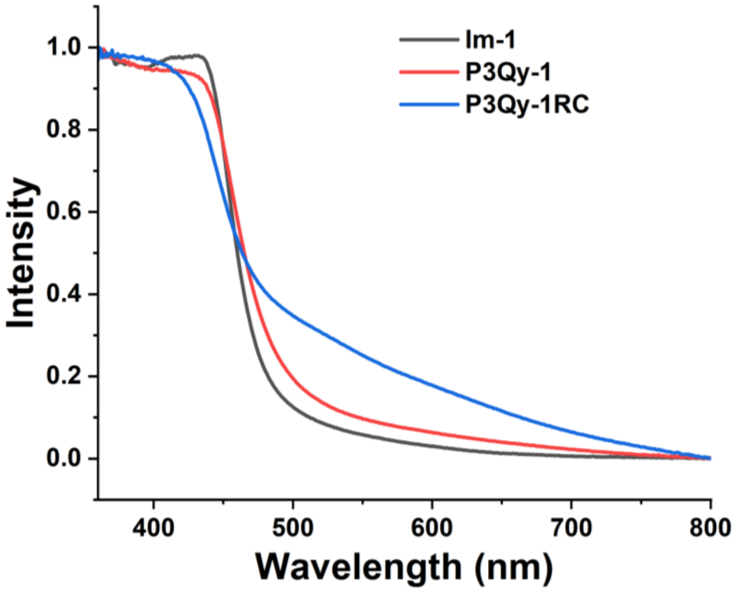

Figure S33. Solid-state UV-vis spectra of **Im-1**, **P3Qy-1** and **P3Qy-1RC**.

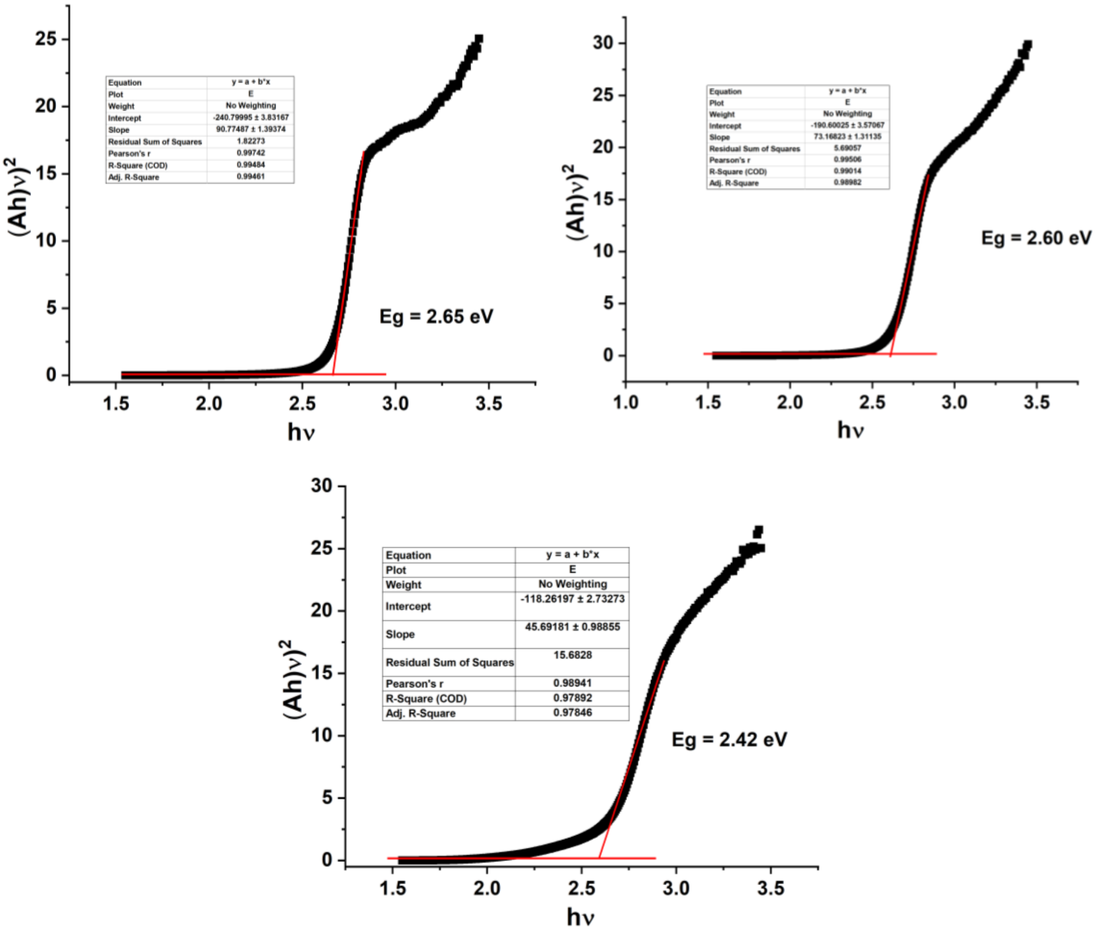

Figure S34. Optical band gap calculation from the intersection of $(\alpha h\nu)^2$ vs hν curve of **Im-1**, **P3Qy-1** and **P3Qy-1RC**.

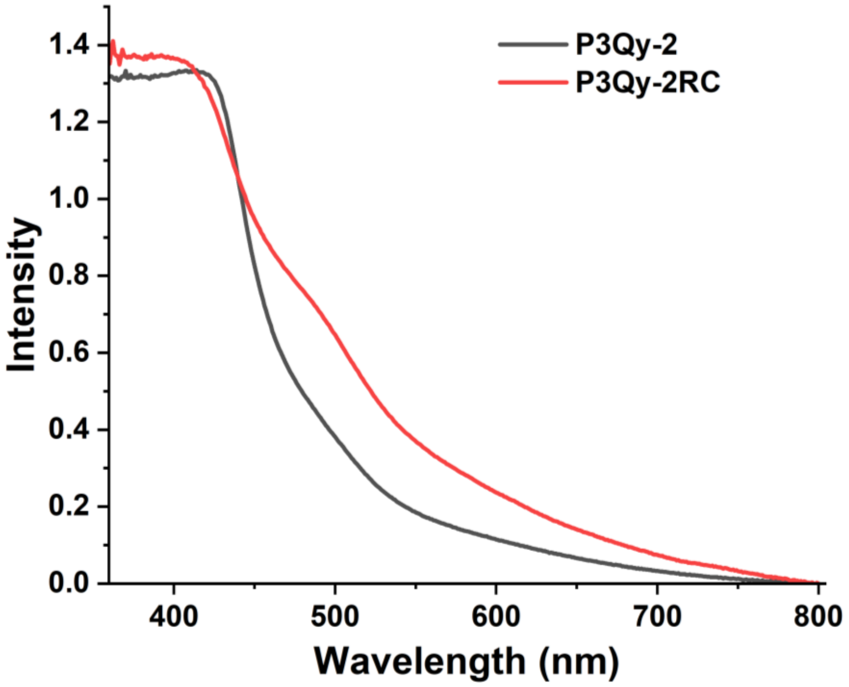

Figure S35. Solid-state UV-vis spectra of **P3Qy-2** and **P3Qy-2RC**.

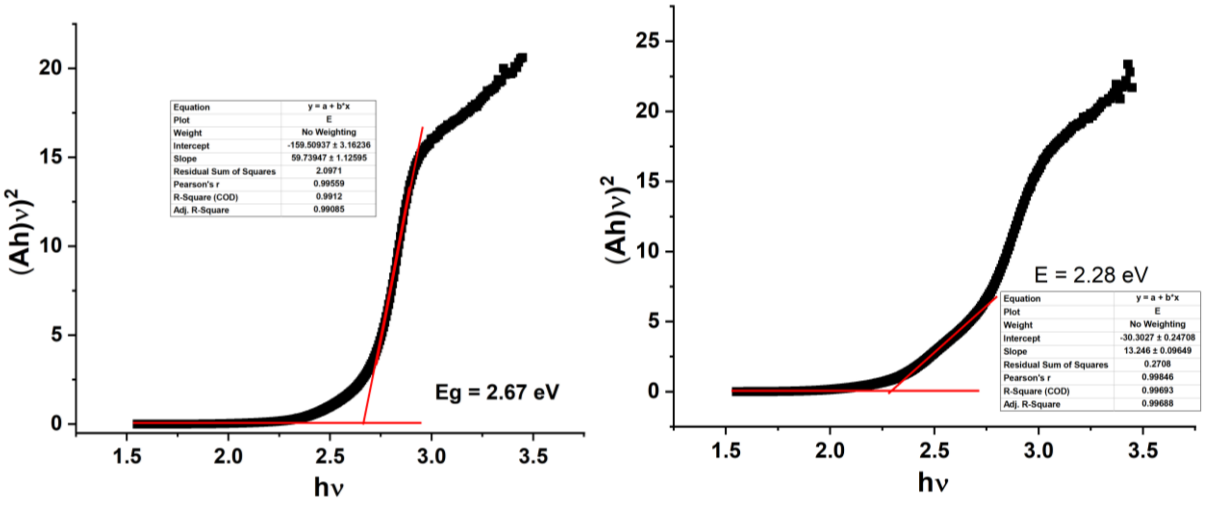

Figure S36. Optical band gap calculation from the intersection of $(\alpha h\nu)^2$ vs hν curve of **P3Qy-2** and **P3Qy-2RC**.

Section S4. Benzene (Bz) and Cyclohexane (Cy) Sorption and Separation by COFs

Benzene and cyclohexane sorption measurement: The benzene and cyclohexane adsorption isotherms were measured at 298 K under continuous adsorption conditions with the DVS Carbon vapor sorption. Prior to the measurement, the samples were activated at 393 K under a high vacuum (10^{-6} Torr) for 12 h. High-purity (>99%) benzene and cyclohexane were used as sources of the vapors. To ensure the absence of any dissolved gas, three freeze–thaw cycles were applied to the two solvents prior to the measurement.

Fluorescence and lifetime measurement: Photoluminescence experiments were performed using Horiba Scientific Fluorolog 3 spectrofluorometer with the stirring mode; time resolved lifetime measurements were performed on Time Correlated Single Photon Counter with a 320 nm picosecond diode laser while the acquire data were analyzed by DAS analysis.

Compared the uptake and selectivity of **P3Qy-1** and **Im-1** from an equimolar Bz/Cy Mixture: An open 1 mL GC vial containing 5 mg of activated **P3Qy-1** and **Im-1** adsorbent was placed in a sealed 20 mL vial containing 2 mL of an equimolar Bz/Cy mixture. Uptake by **P3Qy-1** and **Im-1** was measured over time by socking the COF in $CDCl_3$ and measuring the ratio of Bz or Cy to **P3Qy-1** and **Im-1** by 1H NMR after filtration using 8 mmol mesitylene as an internal standard. Additionally, we measure TGA after individual Bz and Cy sorption.

Recyclability Test: For Recyclability assessment, 20 mg of each COFs was subjected to vapor sorption with an equimolar Bz/Cy mixture upto 10 cycles for 3h. In every cycle, the COFs were regenerates by washing with acetone and heating at 80 ^{o}C. After 10 cycles, both COFs were evaluated for their sorption ability by ^{1}H NMR. After that, COFs was washed and dried for measurement of PXRD and Ar sorption at 87 K.

Configurational Bias Monte Carlo (CBMC) molecular simulation:[2] The structures of **P3Qy-1**, **P3Qy-1RC**, **P4Qy-1** and **Im-1** was assumed to be rigid in the crystallographic position, which is obtained from Pawley refinement PXRD data. The simulation boxes representing **P3Qy-1**, **P3Qy-1RC**, **P4Qy-1** and **Im-1** adsorbent consist of unit cells for Bz and Cy. All the calculations were performed at 298 K. For mixture of vapor, equimolar mixture of Bz and Cy was used in a simulation box of COF. The structure of Bz and Cy was optimized using DFT method. Interatomic interactions were modeled with standard Lennard-Jones potential and Columbic potentials. Lennard-Jones parameters between unlike atom types were computed using the Lorentz-Berthelot mixing rules. The pairwise interactions between host guest atoms of the particular force field, nonbonding parameter has been utilized. The long-range part of electrostatic interactions was handled using the Ewald summation technique with a relative precision of 10^{-6}. Periodic boundary conditions were applied in all three dimensions. For each state point, the CBMC simulation consists of 1 x 10^7 steps to guarantee equilibration, followed by 1 x 10^7 steps to sample the desired thermodynamic properties.

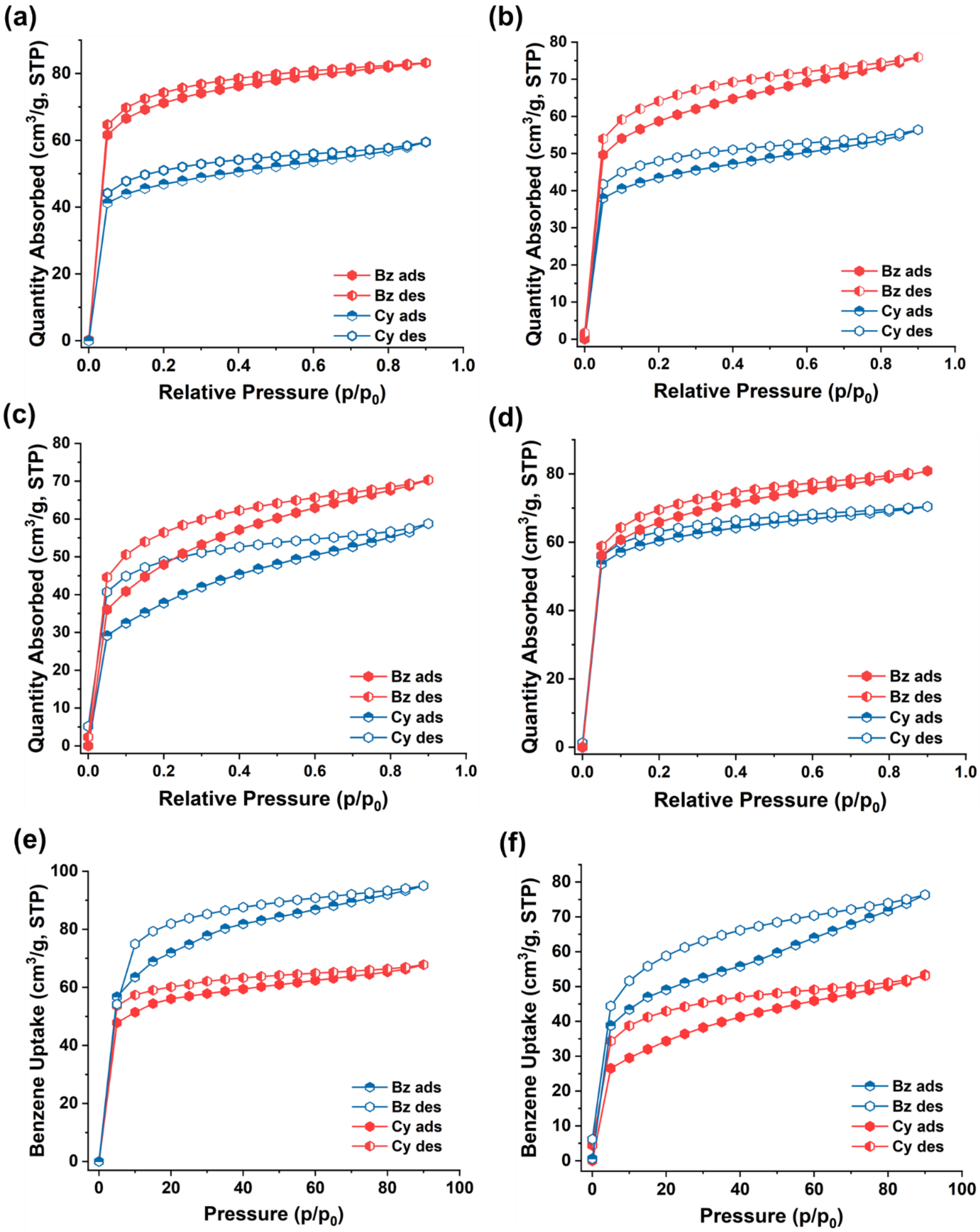

Figure S37. Sorption of Benzene and cyclohexane by (a) **P3Qy-1**, (b) **P3Qy-1RC**, (c) **P4Qy-1**, (d) **Im-1**, (e) **P3Qy-2** and (f) **P3Qy-2RC**.

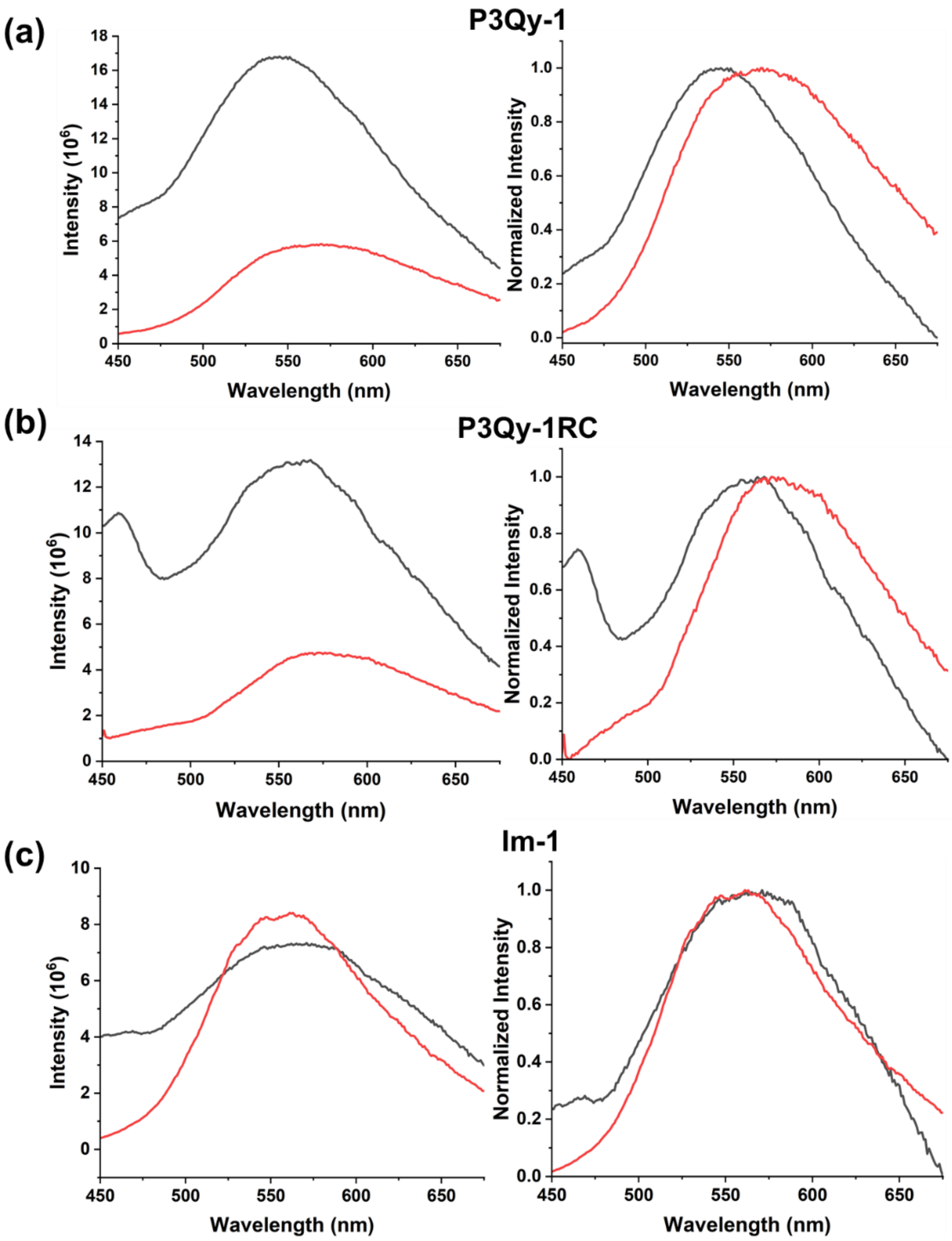

Figure S38. Fluorescence spectra of (a) **P3Qy-1**, (b) **P3Qy-1RC**, and (c) **Im-1** in presence of Bz (red) and Cy (black) (λexc: 370 nm) [Right: Intensity and Left: Normalized Intensity].

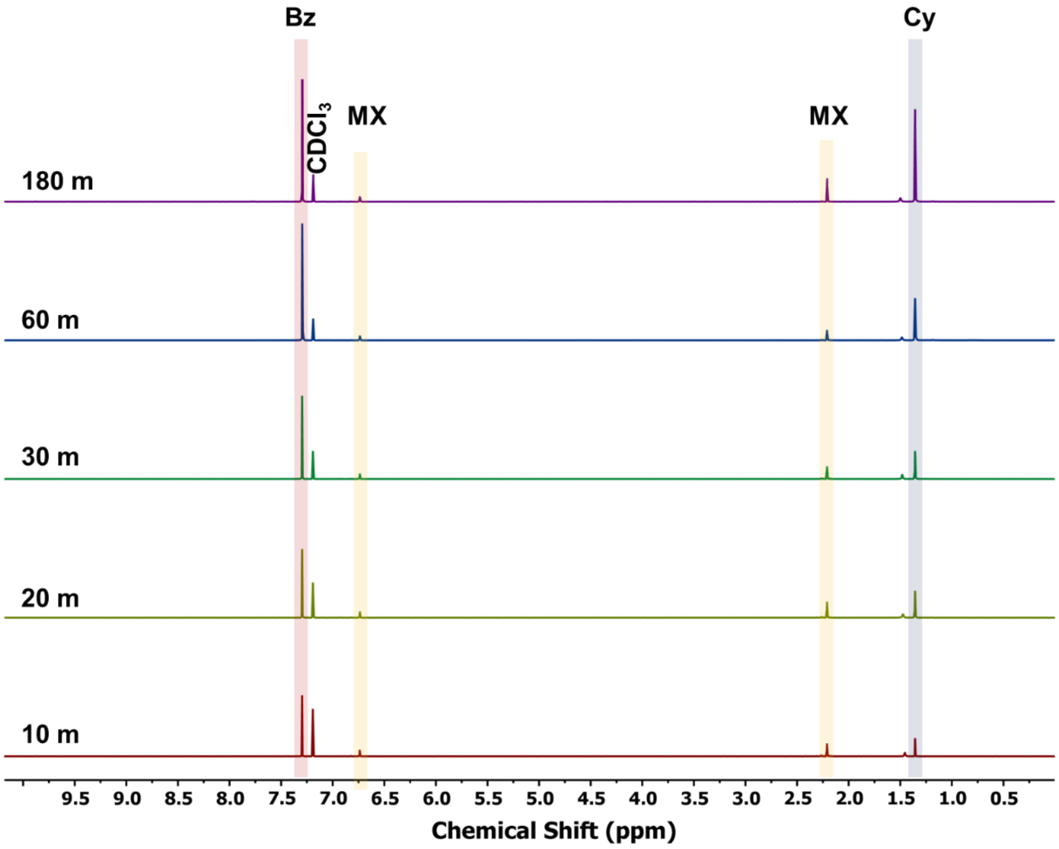

(b)

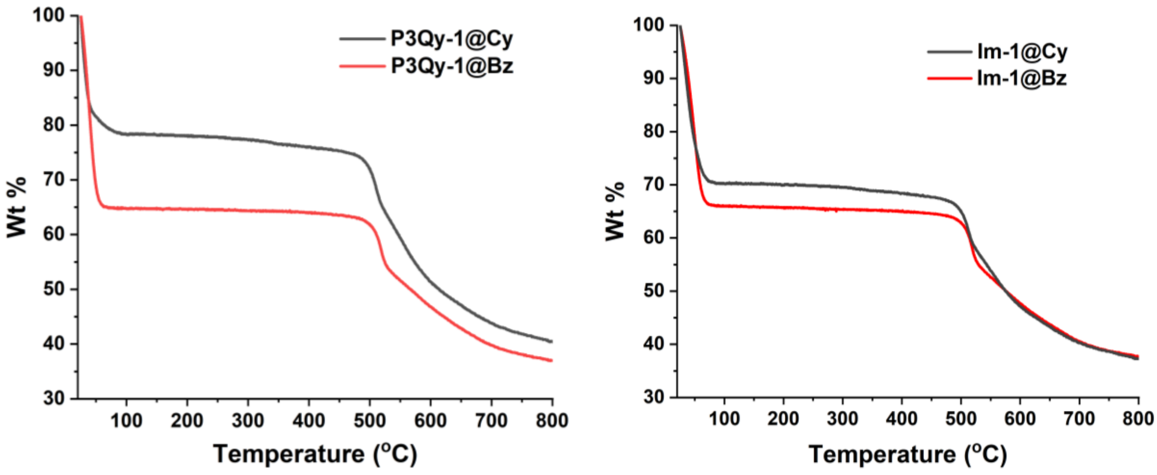

Figure S39. Vapor Phase Bz/Cy (1:1 mixture) sorption: (a) ^{1}H NMR spectra capturing the progress of solvent capture in **P3Qy-1** (Red mark: Bz; Blue mark: Cy; Yellow mark: Mesitylene (MX) as an internal standard); (b) TGA profile of **P3Qy-1**, and **Im-1** in presence of Bz and Cy after 1h vapor sorption.

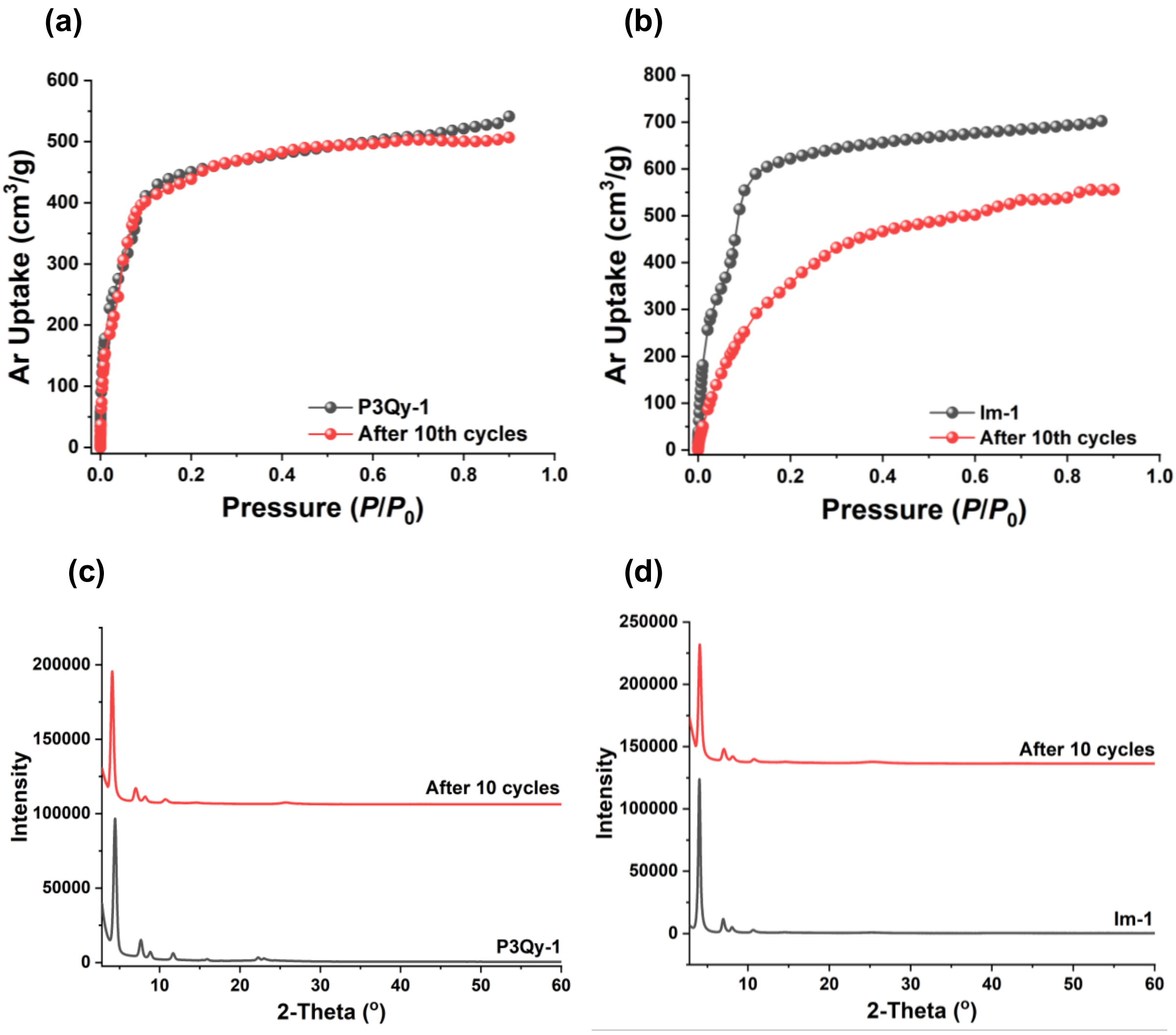

Figure S40. Recyclability test after 10 cycles: Ar adsorption of (a) **P3Qy-1**, and (b) **Im-1**; PXRD of (c) **P3Qy-1**, and (d) **Im-1**.

Table S5. Comparison of Benzene uptake in COF-based systems

COFs	T, K	Ads. Bz, cm^3/g STP (0.9 bar)	Ads. Bz, cm^3/g STP (0.05 bar)	Ads. Cy, cm^3/g STP (0.9 bar)	Reference
**P3Qy-1**	298	83.1	60	59	**This work**
**P3Qy-2**	298	90.1	64	67.7	**This work**
**P4Qy-1**	298	70.3	38	58.7	**This work**
**P3Qy-1RC**	298	75.9	46	53.6	**This work**
**P3Qy-2RC**	298	77	49	53.4	**This work**
**Im-1**	298	80.4	58	70.4	**This work**
COF-300-rt	298	251	85	175	*ACS Appl. Mater. Interfaces* ***2022****, 14, 36, 40890–40901.*
COF-300-st	298	221	70	133	*ACS Appl. Mater. Interfaces* ***2022****, 14, 36, 40890–40901.*
LZU-111	298	214	150	164	*ACS Appl. Mater. Interfaces* ***2022****, 14, 36, 40890–40901.*
COF-1	298	220	25	87	*J. Mater. Chem. A* **2018**, *6*, 16246-16256
COF-IP-10	298	280	40	Negligible	*Chem. Eur. J.* **2016**, *22*, 4931– 4937
TBICOF	298	641.9	82	186.2	*Chem. Mater.* **2019**, *31*, 1584–1596
TTPE-COF	293	131.6	11	NA	*CrystEngComm* **2021**, *23*, 5569– 5574

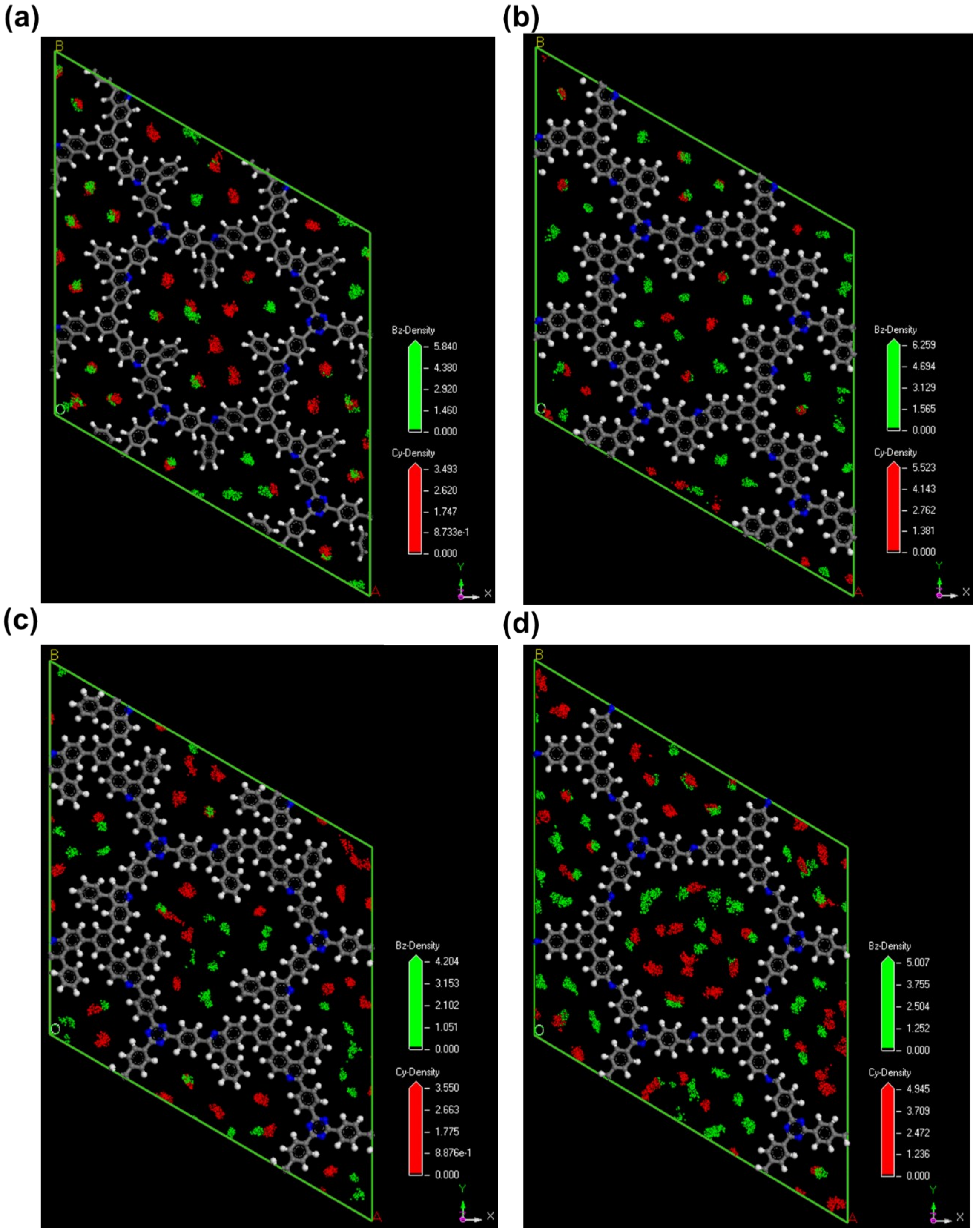

Figure S41. Configurational-Bias Monte Carlo (CBMC) simulation for sorption and separation of equimolar mixture of Bz/Cy in (a) **P3Qy-1**, (b) **P3Qy-1RC**, (c) **P4Qy-1** and (d) **Im-1**.

(a)

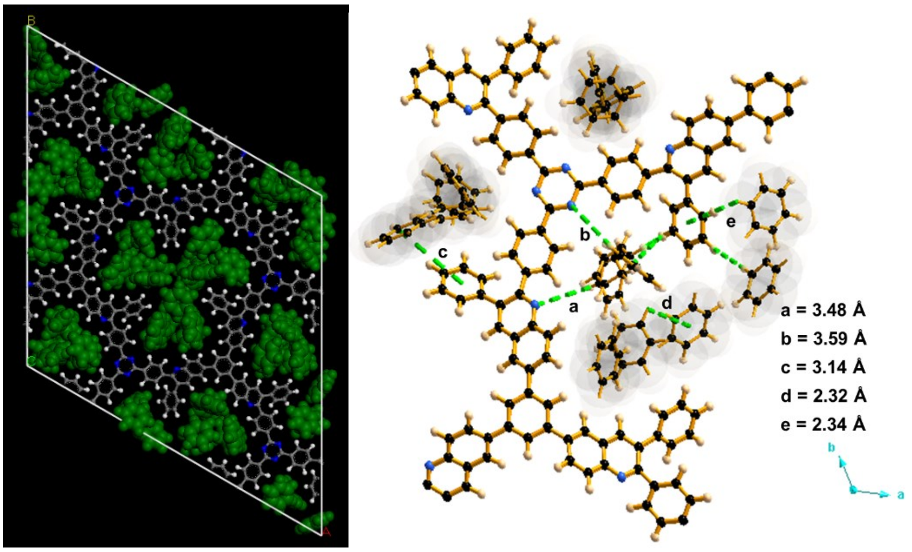
a = 3.48 Å
b = 3.59 Å
c = 3.14 Å
d = 2.32 Å
e = 2.34 Å

(b)

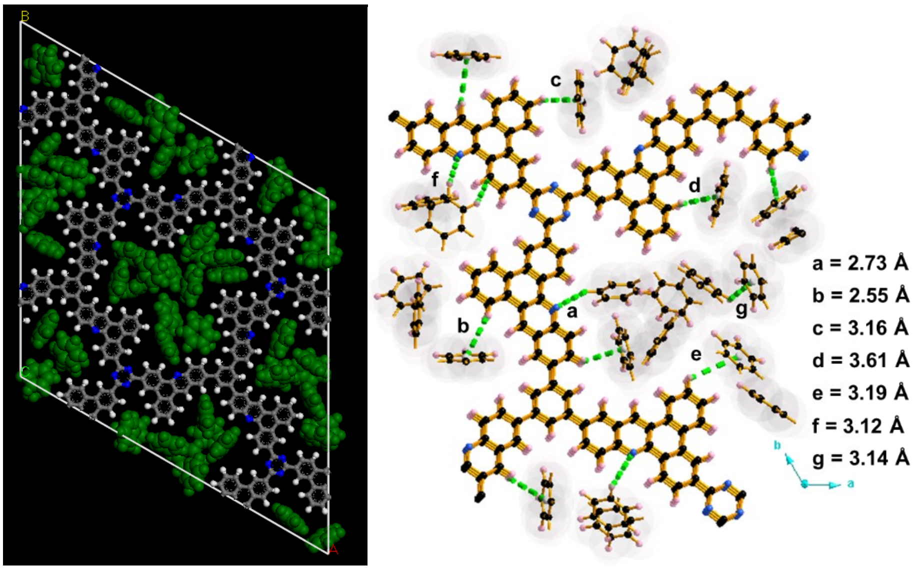
a = 2.73 Å
b = 2.55 Å
c = 3.16 Å
d = 3.61 Å
e = 3.19 Å
f = 3.12 Å
g = 3.14 Å

(c)

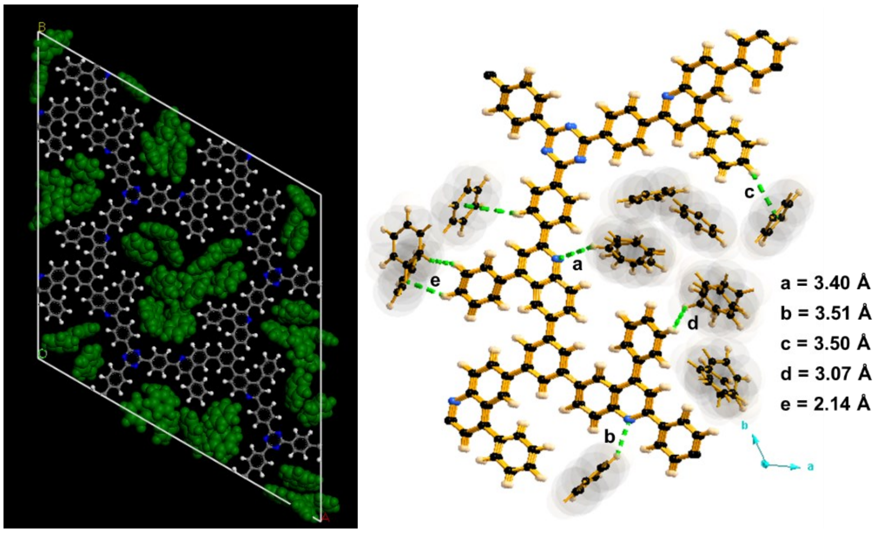
a = 3.40 Å
b = 3.51 Å
c = 3.50 Å
d = 3.07 Å
e = 2.14 Å

(d)

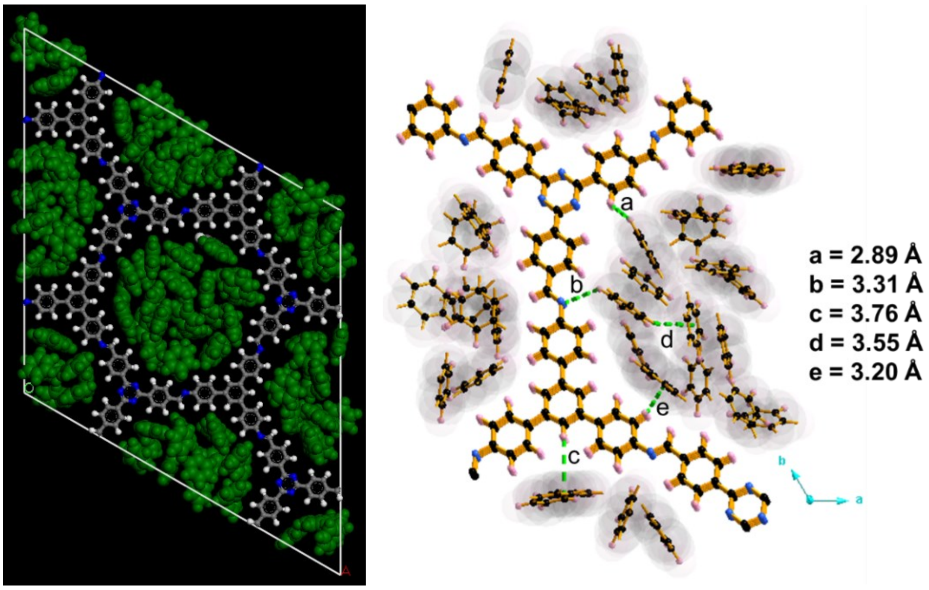
a
b
c
d
e
a = 2.89 Å
b = 3.31 Å
c = 3.76 Å
d = 3.55 Å
e = 3.20 Å
b
a

(e)

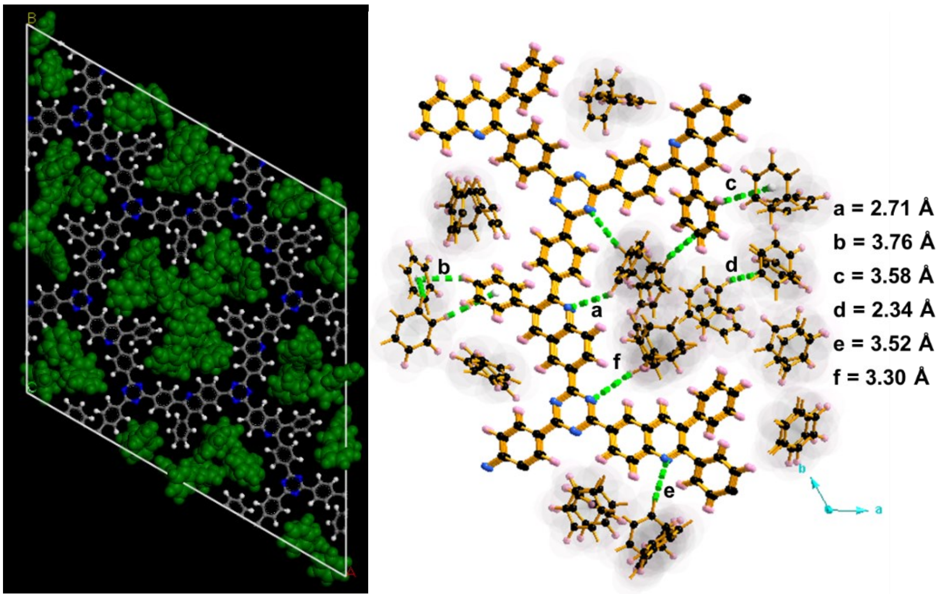
B
A
a
b
c
d
e
f
a = 2.71 Å
b = 3.76 Å
c = 3.58 Å
d = 2.34 Å
e = 3.52 Å
f = 3.30 Å
b
a

(f)

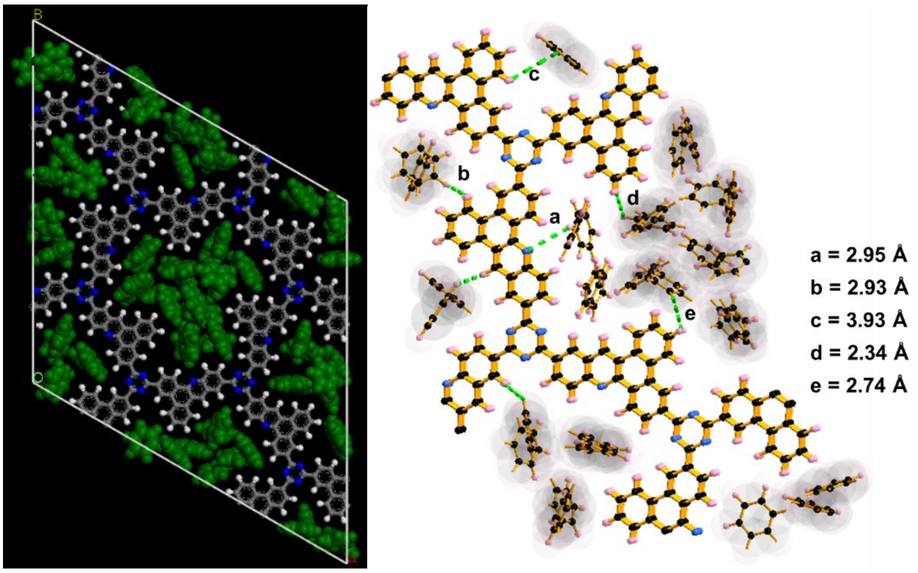

Figure S42. Configurational-Bias Monte Carlo (CBMC) simulation for sorption and interaction of Bz in (a) **P3Qy-1**, (b) **P3Qy-1RC**, (c) **P4Qy-1** (d) **Im-1**, (e) **P3Qy-2** and (f) **P3Qy-2RC**.

References:

(S1) Zhang, Y.; Položij, M.; Heine, T. Statistical Representation of Stacking Disorder in Layered Covalent Organic Frameworks, *Chem. Mater.* **2022**, *34* (5), 2376-2381.

(S2) Bernini, M. C.; Fairen-Jimenez, D.; Pasinetti, M.; Ramirez- Pastor, A. J.; Snurr, R. Q. Screening of bio-compatible metal–organic frameworks as potential drug carriers using Monte Carlo simulations. *J. Mater. Chem. B* **2014**, *2*, 766– 774.
